# Effect of human milk-based fortification in extremely preterm infants fed exclusively with breast milk: a randomised controlled trial

**DOI:** 10.1016/j.eclinm.2023.102375

**Published:** 2024-01-02

**Authors:** Georg Bach Jensen, Magnus Domellöf, Fredrik Ahlsson, Anders Elfvin, Lars Navér, Thomas Abrahamsson

**Affiliations:** a Department of Biomedical and Clinical Sciences, Linköping University and Crown Princess Victoria Children´s Hospital, Linköping, Sweden; b Department of Clinical Sciences, Paediatrics, Umeå University, Umeå, Sweden; c Department of Women´s and Children´s Health, Uppsala University, Uppsala, Sweden; d Department of Paediatrics, Institute of Clinical Sciences, Sahlgrenska Academy, University of Gothenburg, Gothenburg, Sweden; e Department of Paediatrics, Region Västra Götaland, Sahlgrenska University Hospital, Queen Silvia Children's Hospital, Gothenburg, Sweden; f Division of Paediatrics, Department of Clinical Science, Intervention, and Technology (CLINTEC), Karolinska Institutet, Stockholm, Sweden; g Department of Neonatology, Karolinska University Hospital, Stockholm, Sweden

**Keywords:** Preterm infant, Nutrition, Breast milk, Nutrient fortifier, Necrotising enterocolitis, Sepsis

## Abstract

**Background:**

Mortality and severe morbidity remain high in extremely preterm infants. Human milk-based nutrient fortifiers may prevent serious complications and death. We aimed to investigate whether supplementation with human milk-based fortifier (HMBF), as compared to bovine milk-based fortifier (BMBF), reduced the incidence of the composite outcome of necrotising enterocolitis (NEC), sepsis, and mortality in extremely preterm infants exclusively fed human milk.

**Methods:**

In this multicentre, randomised controlled trial at 24 neonatal units in Sweden, extremely preterm infants born between gestational week 22 + 0 and 27 + 6 fed exclusively human breast milk (mother's own and/or donor milk), were randomly assigned (1:1) to receive targeted fortification with either HMBF or BMBF. Randomisation was conducted before the enteral feeds reached 100 mL/kg/day, and was stratified by enrolment site, gestational age, singleton/twin, and sex. The allocation was concealed before inclusion, but after randomisation the study was not blinded for the clinical staff. For the NEC diagnosis, the study group was masked to an independent radiologist, and the final assessment of NEC and culture-proven sepsis was done by a blinded consensus panel review. The primary outcome was the composite of NEC stage II–III, culture-proven sepsis, and mortality from inclusion to discharge, no longer than postmenstrual week 44 + 0, in the intention-to-treat population (ClinicalTrials.gov, NCT03797157).

**Findings:**

Between February 21st, 2019, and May 21st, 2021, 229 neonates were randomly assigned (115 HMBF, 114 BMBF). After exclusion of one infant due to parents’ withdrawal of consent, 228 infants were included in the intention-to-treat analysis. Of the 115 infants assigned to HMBF, 41 (35.7%) fulfilled the criteria of either NEC, sepsis, or death, compared with 39 (34.5%) of 113 infants assigned to BMBF (OR 1.05, 95% CI 0.61–1.81, p = 0.86). Adverse events did not differ significantly between groups.

**Interpretation:**

Supplementation with HMBF, as compared with BMBF, did not reduce the incidence of the composite outcome of NEC, sepsis, or death. Our results do not support routine supplementation with HMBF as a nutritional strategy to prevent NEC, sepsis, or death in extremely preterm infants exclusively fed human milk.

**Funding:**

ALF grant, Prolacta Bioscience, 10.13039/501100004359Swedish Research Council, and Research Council for Southeast Sweden.

**Research in context**

**Evidence before this study**

A diet with exclusive breast milk needs nutritional fortification to meet the requirements of extremely preterm infants. Traditionally, these fortifiers have been based on cow's milk, which has been hypothesised to increase the risk of NEC and sepsis. Human milk-based fortifiers have become available, but the most recent Cochrane analysis stated, that there is insufficient evidence evaluating human milk-based with bovine milk-based fortifier in exclusively breast milk-fed preterm infants, and that randomised trials are needed.

**Added value of this study**

To the best of our knowledge, this is the first prospective randomised controlled trial, powered to evaluate severe outcomes in preterm infants, that compares the effect of human milk-based with bovine milk-based nutrient fortifier, in which both study groups exclusively receive human breast milk. Hence, our finding, that supplementation with human milk-based fortifier did not affect the primary composite outcome of NEC, sepsis, or mortality in extremely preterm infants, is novel and important information for clinicians in the field of paediatrics.

**Implications of all the available evidence**

Routine supplementation with human milk-based fortifier is not superior to bovine milk-based fortifier as a nutritional strategy to prevent NEC, sepsis, or death in extremely preterm infants already receiving formula-free enteral feeding with own mother's milk or donated breast milk.

## Introduction

Complications secondary to preterm birth are the leading cause of death among children under 5 years of age, causing almost one million deaths annually worldwide.^1^ At highest risk are extremely preterm infants (born before 28 weeks of completed gestation), constituting an estimated four percent of all preterm births.^2^ Rates of extremely preterm birth seem to be stable over the last decades in high income countries, and though prenatal and neonatal care have improved considerably, mortality and severe morbidity remain high, even in affluent countries, with a 23% 1-year mortality for infants born before 27 weeks of gestation in the latest Swedish national survey.^3^^,^^4^

Necrotising enterocolitis (NEC), a severe inflammatory condition leading to ischaemia and necrosis of the intestine, with an incidence between 4% and 15% in very low birth weight (<1500 g) infants,^4^, ^5^, ^6^ and late-onset sepsis, with an incidence between 18% and 34% in extremely preterm infants,^7^^,^^8^ remain common and life-threatening conditions in modern neonatal care, and are major risk factors for future neurological impairment.^9^^,^^10^

Nutrition is a key factor for survival, clinical course, and later outcome of high-risk preterm neonates. When feeding extremely preterm infants, the primary choice is the infant's mother's own milk (MOM).^11^ Donor breast milk or preterm formula may be used when mothers provide insufficient amounts of breast milk. A growing literature supports the use of human milk for feeding preterm infants, and there is convincing evidence that the use of human milk reduces the risk of NEC and possibly late-onset sepsis.^12^^,^^13^ Conversely, formula feeding has been shown to increase the risk of NEC.^14^ However, despite the health benefits of human milk, it does not meet the nutritional requirements of extremely preterm infants.^13^ Therefore, protein-containing fortifiers are generally recommended.^15^ Individualised targeted fortification, based on analysed breast milk macronutrient content, has been shown to increase growth velocity of weight, length, and head circumference.^16^ Traditionally, bovine milk-based fortifiers (BMBF) are used, yet there is some evidence, indicating that these fortifiers may have negative effects, such as increasing the risk of NEC, sepsis, and death, compared to human milk-based fortifiers (HMBF).^17^, ^18^, ^19^, ^20^ However, evidence for the use of HMBF in extremely preterm infants is sparse, mostly observational, and with trials including formula-fed infants in the control group, and therefore not truly evaluating only the human milk-based fortifier.^18^^,^^19^^,^^21^^,^^22^

A recent Cochrane analysis was only able to include one randomised controlled trial, that was truly designed to evaluate the impact of a human milk-based fortifier.^22^^,^^23^ It stated that there is insufficient evidence evaluating the effect of HMBF in exclusively breast milk-fed preterm infants, and that randomised trials are needed.

Given this, we conducted the Nordic study on human milk fortification in extremely preterm infants (N-Forte), which aimed to investigate the effect of human milk-based nutrient fortifier on NEC, sepsis, and mortality as a composite measure of severe outcome in extremely preterm infants fed exclusively with breast milk, in a setting where individualised targeted fortification is routinely used.

## Methods

### Study design and participants

The N-Forte was an investigator-initiated, prospective, multicentre, randomised controlled superiority trial. A comprehensive description of the study protocol adhering to SPIRIT 2013 guidelines^24^ has previously been published.^25^ The completion and reporting of the trial is in line with CONSORT 2010 guidelines.^26^

Extremely preterm infants were enrolled at 7 level III neonatal intensive care units (NICUs) in Sweden. Another 17 level I–II neonatal units in the catchment area regions participated ensuring continuation in the event of a transfer from one hospital to another during the study period. Infants were eligible if they were born between 22 + 0 and 27 + 6 weeks of gestation, had survived the first three days of life, and the home clinic of the infant had the logistics of maintaining the study intervention until postmenstrual week (PMW) 34 + 0. Gestational age was based on ultrasonographic screening during pregnancy. To be included, enteral feeds needed to be below 100 mL/kg/day at the day of randomisation.

Infants were excluded if any of the following were known at the time of enrolment: lethal or complicated malformation, chromosomal anomaly, no realistic hope for survival (based on the discretion of the responsible neonatologist), gastrointestinal malformation, abdominal surgery, participation in another intervention trial aiming at influencing growth, nutrition, feeding intolerance, NEC, and sepsis. In addition, infants having nutrient fortifier or formula prior to enrolment were excluded.

The trial was conducted according to ICH/GCP guidelines and was approved by the regional ethical review board in Linköping, Sweden (Dnr 2018/193-31, Dnr 2018/384-32). Oral and written informed consent was obtained from legal guardians of all eligible infants before randomisation.

### Randomisation

Enrolment was done by clinicians at each level III NICU. A secure, web-based randomisation service centre was used by the attendant physician, randomize.net (Interrand, Ottawa, Ontario, Canada). Participants were randomly assigned (1:1) to receive either HMBF or a standard BMBF before oral feeds had reached 100 mL/kg/day. An adaptive randomisation scheme, based on the method of minimisation, was used. This included a biased-coin randomisation scheme as needed in the adaptive scheme. Randomisation was stratified by primary enrolment site, gestational week (22 + 0 – 24 + 6 or 25 + 0 – 27 + 6), singleton/twin, and sex. Twins were randomised together thus allocated to the same study group. Recruitment was ended when the target sample size had been reached.

The study group was not masked to the caregivers, clinical staff, or study nurses. Targeted fortification of breast milk and the difference in nutrient content between the study product and the standard fortification made it unfeasible to blind the study after randomisation.^25^ The criteria for the primary outcomes of culture-proven sepsis and NEC were therefore objectively assessed. For the NEC diagnosis, the study group was masked to an independent radiologist, and the final assessments of both NEC and culture-proven sepsis were done by a blinded consensus panel review consisting of the investigators. The secondary outcome retinopathy of prematurity (ROP) was diagnosed by an independent ophthalmologist.

### Procedures

A detailed description has previously been published.^25^ In brief, the active group received HMBF (Humavant +6, supplied by Prolacta Bioscience, California, USA) and the control group received the standard BMBF of the responsible NICU. Individualised targeted fortification was applied at all study centres. The attending physician and/or dietitian prescribed the enteral nutrition daily during the NICU stay, including the source of breast milk (MOM and/or donor milk), total volume, and the desired level of fortification based on individual analyses of the true protein content in the breast milk, when such analyses had been done. The level of fortification was prescribed stepwise, according to local guidelines, to achieve appropriate protein intake and to ensure that intakes of all nutrients were within recommended ranges. The daily level of fortification for each infant was based on protein intake and the target was initially 4.0–4.5 g/kg/day with a gradual decrease in intake with approaching term equivalent age. The intervention period ended at PMW 34 + 0. If protein fortification was still needed hereafter, there was a transition period in the HMBF group where the fortification of the breast milk was gradually substituted with standard bovine-based fortifier during a 5-day period. Macronutrient analyses of MOM were performed weekly using an infrared breast milk analyser (Miris, Uppsala, Sweden). Breast milk analyses of donor breast milk were performed once for each batch. To assist in calculating the individual nutritional needs the computer-aided nutrition calculation programme Nutrium (Nutrium AB, Umeå, Sweden) was used. This was also used in the prescription of other important supplements (*e.g.,* vitamins, iron, calcium and phosphorous) when needed. When fat supplementation was needed, the infants receiving the HMBF were supplemented with the human milk-based caloric fortifier Humavant CR (Prolacta Bioscience, California, USA) while the infants receiving BMBF were supplemented with the standard lipid products used at the unit. The infants should not be fed with formula during the intervention period, which ended at PMW 34 + 0.

The enrolled infants were characterised with clinical data including growth, feeding intolerance, use of enteral and parenteral nutrition, treatments, antibiotics, and complications, collected daily in a study specific case report form (CRF) from birth until discharge from the hospital (not longer than PMW 44 + 0). Background characteristics are displayed in Table 1.

**Table 1:** Baseline characteristics of the intention-to-treat population.

	HMBF (n = 115)	BMBF (n = 113)
**Mother**		
Caesarean section	70 (61%)	67 (59%)
Maternal smoking during pregnancy	5/103 (5%)	7/102 (7%)
Preterm premature rupture of membranes^a^	35 (30%)	31 (27%)
Chorioamnionitis	27 (24%)	16 (14%)
Preeclampsia	10 (9%)	17 (15%)
Maternal antibiotics	76/115 (66%)	72/112 (64%)
Antenatal betamethasone^b^	80 (70%)	76 (67%)
**Infant**		
Gestational age (weeks)	25.6 (24.6–26.7)	26.0 (24.5–27.1)
Birth weight (g)	793 (212)	787 (207)
Birth weight (z-score)	−0.75 (−1.29–0.05)	−0.74 (−1.61–0.15)
Small for gestational age	13 (11%)	18 (16%)
Female sex	54 (47%)	52 (46%)
5-min Apgar score^c^	7 (5–8)	7 (5–8)
Multiple birth	20 (17%)	20 (18%)
Respiratory distress syndrome	105 (91%)	108 (96%)
Surfactant treatment	94 (82%)	93 (82%)
Mechanical ventilation before inclusion	88 (77%)	85 (75%)
Born at NICU level I–II	28 (24%)	27 (24%)
Major congenital malformation	1/114 (1%)	3/113 (3%)
Inclusion site		
Göteborg	28 (48%)	30 (52%)
Linköping	17 (50%)	17 (50%)
Stockholm, Huddinge	6 (67%)	3 (33%)
Stockholm, Solna	15 (54%)	13 (46%)
Umeå	18 (44%)	23 (56%)
Uppsala	26 (54%)	22 (46%)
Örebro	5 (50%)	5 (50%)

Data are n (%), n/N (%), mean (SD), or median (IQR). HMBF = human milk-based fortifier. BMBF = bovine milk-based fortifier. There were no significant differences between the treatment groups for any characteristic.

a Rupture of membranes >1 h before contractions stated.

b At least one dose of betamethasone given at least 24 h before delivery.

c Two infants did not get a 5-min Apgar score and were excluded from the analysis.

### Outcomes

The primary outcome was a composite of NEC stage II–III (according to Bell's criteria),^27^ culture-proven sepsis and mortality during the study period, from inclusion to discharge no longer than to PMW 44 + 0. For culture-proven sepsis, a positive blood, urine, or cerebrospinal fluid culture, together with both clinical deterioration and a laboratory inflammatory response (white blood cell count <5 or >20 × 10^9^ cells/L or total platelet count <100 × 10^9^ cells/L or C-reactive protein >15 mg/L), were required to fulfil the criteria. Secondary outcomes and covariates are displayed in Table 2. Bronchopulmonary dysplasia (BPD) was defined as the need of extra oxygen, high flow nasal cannula, CPAP or mechanical ventilation at PMW 36 + 0. ROP was diagnosed after PMW 42 + 0 according to international classification into stage I–V. Intraventricular haemorrhage, assessed with ultrasound, was classified into grade I–IV. Periventricular leukomalacia was assessed with ultrasound and magnetic resonance imaging. For both primary and secondary outcomes of morbidity, only events occurring after the infant had been randomly assignment were taken into consideration. Classification of causes of death was done as described by Patel et al.^28^

**Table 2:** Primary and secondary outcomes of the intention-to-treat population.

	HMBF (n = 115)	BMBF (n = 113)	p value^a^
**Primary outcome**			
Composite of necrotising enterocolitis (NEC), culture-proven sepsis, and mortality	41 (35.7%)	39 (34.5%)	0.86
**Secondary outcomes**			
*Clinical variables for morbidity*			
NEC II–III	8 (7.0%)	9 (8.0%)	0.77
NEC II–III onset (day of life)	25 (16–32)	14 (9–47)	0.42
NEC III	5 (4.3%)	7 (6.2%)	0.53
NEC, surgical	4 (3.5%)	4 (3.5%)	1.00
Death	7 (6.1%)	13 (11.5%)	0.15
Culture-proven sepsis	33 (28.7%)	28 (24.8%)	0.50
Culture-proven sepsis (day of life)	12 (9–24)	18.5 (12–29)	0.079
Composite of NEC and culture-proven sepsis	38 (33.0%)	35 (31.0%)	0.74
Suspected sepsis, not culture-proven	32 (27.8%)	36 (31.9%)	0.51
Suspected sepsis, not culture-proven (day of life)	36.5 (21–51)	27 (17–45)	0.31
Culture-proven or suspected sepsis	55 (47.8%)	51 (45.1%)	0.68
Bronchopulmonary dysplasia	60/108 (55.6%)	66/102 (64.7%)	0.18
Retinopathy of prematurity	50/113 (44.2%)	47/110 (42.7%)	0.82
Retinopathy of prematurity, stage III–V	29/113 (25.7%)	25/110 (22.7%)	0.61
Mortality and morbidity index^b^	78 (67.8%)	85 (75.2%)	0.22
Periventricular leukomalacia	7 (6.1%)	5 (4.4%)	0.57
Intensive care (days)^c^	49 (29–73)	44 (27–63)	0.41
Mechanical ventilation (days)^d^	7 (2–28)	4 (1–21)	0.22
Weight at PMW 34 + 0 (g)	1965 (1777–2190)	1910 (1751–2121)	0.13
Postmenstrual age at discharge (weeks)^e^	41.9 (39.0–44.0)	42.2 (39.0–44.0)	0.98
**Feeding intolerance**			
Feeding interruption ≥12 h	36 (31.3%)	41 (36.3%)	0.43
Feeding reduced >50%	50 (43.5%)	41 (36.3%)	0.27
Feeding interrupted or reduced	52 (45.2%)	48 (42.5%)	0.68
Gastric aspirates ≥100% of prefeed volume	50 (43.5%)	51 (45.1%)	0.80
Stool frequency (stools/day)	3.4 (1.1)	3.4 (1.0)	0.61
Time to reach full enteral feeds, 150 mL/kg/d (days)^f^	10 (8–15)	10 (7–13)	0.24
Time to reach full enteral feeds, first of three days (days)^g^	10 (8–17)	10 (8–13)	0.27
**Covariates (until PMA 34 + 0)**			
Start of fortification (day of life)^h^	6 (5–8)	7 (5–8)	0.26
Enteral intake at start of fortification (ml/kg/d)^i^	83 (68–102)	92 (75–114)	0.010
Donor milk proportion (%)^j^	2.2 (0.5–49.6)	7.3 (0.5–75.7)	0.15
Intraventricular haemorrhage	36 (31.3%)	40 (35.4%)	0.51
Intraventricular haemorrhage (grade 3–4)	11 (9.6%)	6 (5.3%)	0.22
Postnatal betamethasone	26 (22.6%)	25 (22.1%)	0.93
Inotropic agents	21 (18.3%)	21 (18.6%)	0.95
Antibiotics (days)	16 (8–23)	12 (7–20)	0.37
Insulin	17 (14.8%)	19 (16.8%)	0.67
Probiotics	5 (4.3%)	8 (7.1%)	0.37
Proton pump inhibitors	18 (15.7%)	15 (13.3%)	0.61
Treatment with opioids	69 (60.0%)	62 (54.9%)	0.43
Central venous line (days)^k^	14 (9–30)	12 (8–21)	0.26
PICC line (days)	13 (8–22)	13 (7–23)	0.75

Data are n (%), n/N (%), mean (SD), or median (IQR). Intention-to-treat. For NEC, death, and sepsis (any kind) only events after time of inclusion are presented. HMBF = human milk-based fortifier. BMBF = bovine milk-based fortifier. NEC = necrotising enterocolitis. PMW = postmenstrual week. PICC = peripherally inserted central catheter.

a The two-tailed Student's t-test to compare means, the chi-square test (or Fisher's exact test if the expected count was less than five) to compare frequencies, and the non-parametric Mann–Whitney test to compare skewed distributions.

b Composite requiring any of the following: death, NEC stage II–III, culture-proven sepsis, bronchopulmonary dysplasia or retinopathy of prematurity stage III–V.

c Number of days with intensive care: need of respirator or CPAP/NIPPV until discharge (not later than PMW 44 + 0).

d Until PMW 34 + 0.

e PMW 44 + 0 at latest; survivors only.

f Only if full enteral was reached (HMBF, n = 110; BMBF n = 107).

g Only if full enteral was reached (HMBF, n = 107; BMBF, n = 105).

h Infants receiving no fortification were excluded.

i Enteral intake at the day leading up to start of fortification. Infants receiving no fortification were excluded.

j Median proportion of donor milk volume (not including HMBF) to the total human milk (*i.e*. donor milk and mother's own milk) volume received by the infants during the study period until PMW 34 + 0.

k Including PICC line.

Study monitoring was performed by Fravil Clinical Consulting, Stockholm, Sweden, which was independent from the sponsor and had no competing interests. Moderate and severe adverse events (SAE) until discharge were recorded and reported. In addition, the investigator or the attending physicians at the study sites were required to report any suspected unexpected severe adverse reaction (SUSAR) to the coordinating principal investigator within 24 h. SUSARs were then reported to the manufacturer and the data safety monitoring board (DSMB).

### Statistical analysis

Based on the Swedish neonatal quality register (www.snq.se) and available literature,^18^, ^19^, ^20^ power calculations showed that at least 101 infants in each group were required to detect a reduction in the primary composite outcome from 47% in the BMBF group to 28% in the HMBF group at a 5% level of significance and 80% power. Hence, the target sample size was calculated at 222 infants allowing for a 10% dropout rate. Due to uncertainties in the pre-estimated effect size, an interim analysis was performed by an independent statistician when 150 infants had fulfilled the study period.^25^ This potentially allowed for an increase (never decrease) in sample size, with 322 infants as a predetermined upper limit, based on the primary outcome. However, the analyses did not result in an increase in sample size.

The two-tailed Student's t-test was used to compare means, the chi-square test (or Fisher's exact test if the expected count was less than five) to compare frequencies. The non-parametric Mann–Whitney test was used to compare skewed distributions. Logistic regression was performed for primary and secondary categorical outcomes to incorporate adjustment variables and interactions.

Adjustments were made for gestational age for its known association to the primary outcome and some of the measures of morbidity. For the outcome of BPD, testing for a possible interaction effect between treatment group and gestational age was conducted with logistic regression. For this analysis the variable of gestational age was dichotomised. For the categorical variables gestational age at birth (week) and inclusion site, the Cochran-Mantel-Haenszel (CMH) test was used as test of conditional independence between groups. Adverse events were adjusted for multiple comparison with the Benjamini and Hochberg method allowing for a 5% false discovery rate. No adjustment for multiple comparison was done for outcomes for which a specific hypothesis existed.

Primary and secondary outcomes were analysed for an intention-to-treat population. A per-protocol analysis was also performed. The per-protocol population included only events with an onset from the first day of fortification, excluding infants with protocol violation (withdrawal of consent before PMW 34 + 0, formula before PMW 34 + 0, lost to follow-up), or infants that did not receive the study product.

All analyses were performed in SPSS v27.0 for Mac (IBM Corp, Armonk, NY, USA). The trial was strictly monitored by the DSMB, which was notified of any SAE. The incidence of SAE was assessed by the DSMB to make interim safety analyses after 50, 100, and 150 completed CRFs until discharge. The trial was registered with ClinicalTrials.gov NCT03797157.

### Role of funding source

The funder of the study had no role in study design, data collection, data analysis, data interpretation, or writing of the report.

## Results

**Fig. 1:**
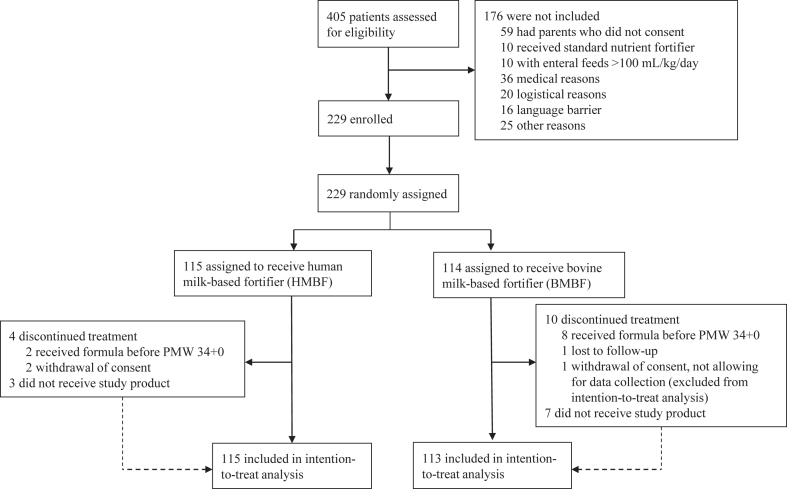
Flow chart of the trial. HMBF = human milk-based fortifier. BMBF = bovine milk-based fortifier.

Between February 21st, 2019, and May 21st, 2021, with a suspension in screening between March 23rd, 2020, and July 27th, 2020, for logistical reasons due to the COVID-19 pandemic, 405 extremely preterm infants were screened of which 229 patients were randomly assigned to the study groups (Fig. 1). After secondary exclusion of one infant (after parents withdrew consent, not allowing for data collection), 228 infants were included in the intention-to-treat analysis. There were no significant differences in baseline characteristics of either mothers or infants between the groups (Table 1).

The primary outcome, the composite of NEC stage II–III, culture-proven sepsis, and mortality during the study period, did not significantly differ between the two treatment groups (p = 0.86). Of the 115 infants assigned to HMBF, 41 (35.7%) infants fulfilled the criteria of either NEC stage II–III, culture-proven sepsis, or death, compared with 39 (34.5%) of 113 infants assigned to BMBF (Table 2). No significant confounding effects could be found for the stratified categorical variables of study inclusion site or gestational age at birth (week), when investigated with the CMH test (Supplementary Table S1). This applied both to the primary outcome and when testing for the intrinsic variables, NEC stage II–III, culture-proven sepsis, and mortality, separately. There were no significant unadjusted differences in secondary outcomes such as mortality, NEC stage II–III, culture-proven sepsis, moderate to severe BPD, ROP stage III–IV, the mortality and morbidity index, number of days with intensive care, or PMW at discharge (Table 2). Details of causes of death are shown in Table 3. Neither were there any significant differences in measures of feeding intolerance with equal median time of 10 days to reach full enteral feeds in both groups (Table 2). Adverse events did not differ between groups after correction for multiple comparisons (Table 4). Out of the 228 infants included in the intention-to-trat analysis, only 10 infants received formula prior to PMW 34 (1 infant at PMW 29, 4 infants at PMW 32, and 5 infants at PMW 33).

**Table 3:** Causes of death.

Study group	Primary cause of death^a^	Subcause (when applicable)	Age of death (days)
HMBF	NEC		18
	Infection	Culture-proven sepsis	39
	RDS	RDS with massive pulmonary haemorrhage	2
	RDS	RDS with severe intracranial haemorrhage	6
	BPD	BPD with infection (sepsis/meningitis)	31
	Other	Cardiac tamponade	24
	Unknown		67
BMBF	NEC		5
	NEC		11
	NEC		15
	NEC	NEC with sepsis	18
	NEC		110
	Infection	Meningitis	29
	Infection	Culture-proven sepsis	37
	BPD	BPD with infection (sepsis)	60
	BPD		103
	Central nervous system (CNS) injury	Severe intracranial haemorrhage with post haemorrhagic hydrocephalus	12
	Other	PPHN	7
	Other	Volvolus	15
	Other	Volvolus	84

a Classification according to Patel et al.^28^ HMBF = human milk-based fortifier. BMBF = bovine milk-based fortifier. NEC = necrotising enterocolitis. RDS = respiratory distress syndrome. BPD = bronchopulmonary dysplasia. PPHN=Persistent pulmonary hypertension of the newborn.

**Table 4:** Adverse events.

	HMBF (n = 115)	BMBF (n = 113)	p value^a^
Abdominal surgery, all diagnoses	10 (9%)	10 (9%)	0.98
Spontaneous perforation	2 (2%)	0 (0%)	0.50
Volvolus	1 (1%)	4 (4%)	0.21
Meconium ileus	2 (2%)	1 (1%)	1.00
Other^b^	1 (1%)	1 (1%)	1.00
Pulmonary haemorrhage	4/114 (4%)	3/113 (3%)	1.00
Pneumothorax	3/114 (3%)	3/113 (3%)	1.00
Pleural effusion	1/114 (1%)	0/113 (0%)	1.00
Apnoea, moderate to severe	24/86 (28%)	30/90 (33%)	0.44
Pulmonary hypertension	13/114 (11%)	21/113 (19%)	0.13
Significant symptomatic haemorrhage	0/114 (0%)	3/113 (3%)	0.12
Bleeding tendency	0/114 (0%)	5/113 (4%)	0.029
Arterial hypotension	4/114 (4%)	9/113 (8%)	0.16
Persistent ductus arteriosus, receiving treatment	44 (38%)	46 (41%)	0.71
Circulatory arrest	5/114 (4%)	7/113 (6%)	0.54
CMV infection	2/114 (2%)	3/113 (3%)	0.68
Other infections^c^	20/114 (18%)	19/113 (17%)	0.88
Periventricular echodensities	7/114 (6%)	8/113 (7%)	0.78
Posthaemorrhagic hydrocephalus	1 (1%)	8 (7%)	0.018
Hypernatremia	29 (25%)	23 (20%)	0.38
Hyperbilirubinemia	102/114 (90%)	99/113 (88%)	0.68
Nephrocalcinosis	5/113 (4%)	3/113 (3%)	0.72
Iatrogenic complication	5/114 (4%)	4/113 (4%)	1.00

Data are n (%), or n/N (%). HMBF = human milk-based fortifier. BMBF = bovine milk-based fortifier. NEC = necrotising enterocolitis.

a The chi-square test (or Fisher's exact test if the expected count was less than five) was used to compare frequencies. There were no significant differences between the treatment groups for any event when correcting for multiple comparison with the Benjamini and Hochberg method allowing for a 5% false discovery rate.

b Not including NEC; one infant in the HMBF group was diagnosed with malrotation and one infant in the BMBF group was diagnosed with an incarcerated inguinal hernia.

c Not including NEC, sepsis (culture-positive or suspected), pneumonia, or meningitis.

Although gestational age did not differ between groups, we performed logistic regression analysis with adjustment for gestational age due to its known inverse association to the primary outcome and some of the measures of morbidity (Supplementary Table S2). This did not change the primary outcome (cOR 1.05, 95% CI 0.61–1.81, p = 0.86, aOR 0.97, 95% CI 0.54–1.73, p = 0.92). After adjustment for gestational age the HMBF group had decreased odds of having BPD at PMW 36 (aOR 0.54, 95% CI 0.29–1.00, p = 0.049). We therefore further investigated a possible interaction effect between treatment group and the dichotomised variable of gestational age (weeks 22–25 [n = 117] by weeks 26–27 [n = 111]) but found no evidence of such an interaction (p = 0.19) on the outcome of BPD.

The per-protocol population comprised 205 patients (108 in the HMBF group and 97 in the BMBF group) (Fig. 1). The per-protocol analysis, only considering events with an onset from the first day of fortification, and excluding infants with protocol violation, or infants that did not receive breast milk fortification anytime until PMW 34 + 0, revealed similar results (Supplementary Table S3). Adjustments for gestational age did not change this (Supplementary Table S4).

The day of life when supplementation of the study product was started did not significantly differ between groups, however the enteral intake at the day leading up to start of fortification was higher in the BMBF group (Table 2). To examine the potential interaction on primary and secondary outcomes we continued with logistic regression analysis adjusted for enteral intake at start of fortification. This analysis did not reveal an effect of enteral intake volume at start of fortification on primary or main secondary outcomes (Supplementary Table S5).

## Discussion

The results of this multicentre trial did not show any effect on the composite outcome (NEC, sepsis, or mortality) in extremely preterm infants, comparing a group of infants supplemented with human milk-based fortifier with a group of infants supplemented with bovine milk-based fortifier. Furthermore, no significant effects were observed on secondary outcomes. To our knowledge, this is the largest prospective randomised controlled trial evaluating HMBF in a formula-free population, and the first with power to evaluate severe outcomes in preterm infants. Four previous randomised trials have examined the impact of human milk-based vs bovine-based diets, of which only one study, by O'Connor et al., was truly designed to evaluate the impact of a human milk-based fortifier, since the infants received formula in the control group in the other three.^18^^,^^19^^,^^21^^,^^23^ The former, a Canadian RCT on HMBF vs BMBF, was not powered to demonstrate a significant effect of severe complications such as NEC and late-onset sepsis or mortality. Moreover, the sample size was smaller (n = 127), and the infants were of higher gestational age (mean 27.7 weeks) than in the present study. In another trial by Sullivan et al., there was no effect of HMBF on the primary outcome feeding intolerance, while the secondary outcome NEC was lower in the HMBF than in the BMBF group.^18^ Some of the infants in the control group were given formula which is well known to increase the risk of NEC.^14^ In a subsequent post hoc analyses of this trial,^17^ only including infants receiving MOM prefortification, the NEC incidence in the HMBF group was in line with previous trials with similar case-mix of very low birth weight infants exclusively fed human breast milk receiving BMBF, 4%,^29^ while the NEC incidence in the BMBF group was unexpectedly high, 16%, which makes the interpretation of this post hoc analysis difficult. A recent meta-analysis found that use of HMBF compared with BMBF reduced the risk of NEC.^30^ However, the authors included the study by Sullivan et al.,^18^ where some of the infants in the control group were given formula, and therefore the meta-analysis did not truly evaluate the effect of the fortifier *per se*. This raises the question, whether a supposed decrease in NEC incidence can be attributed to protective properties of HMBF or detrimental effects of BMBF. A Cochrane review, evaluating multi-nutrient fortified human milk, compared with unfortified human milk, with low-certainty evidence, found no association between BMBF and NEC.^31^

A Cochrane review in 2019, comparing HMBF with BMBF in exclusively breast milk-fed preterm infants, was only able to include one randomised controlled trial that was truly designed to evaluate the impact of a human milk-based fortifier, namely the trial by O'Connor et al.,^23^ and therefore stated, that there is insufficient evidence.^22^ O'Connor et al. did find a significant (p = 0.04) reduction in the secondary outcome of severe ROP in the HMBF group, a finding that we were not able to reproduce in the present trial.

We chose a composite of NEC, culture-proven sepsis, and death as the primary endpoint. The rationale being, that NEC and sepsis share many pathogenic mechanisms, and that the diagnosis of NEC and sepsis often is a continuum, and with previous results indicating a positive effect of HMBF on both NEC and sepsis.^20^ Furthermore, mortality constitutes an intrinsic censoring effect in infants at high risk of developing severe sepsis or NEC. Our trial did not have power to study NEC as a separate outcome. For high-level evidence, a randomised trial on HMBF vs. BMBF in infants exclusively fed breast milk, powered to evaluate effects on NEC, would be ideal. With a NEC incidence rate of 7.46% (17/228) in our data, a sample size of 1190 infants born before 28 weeks of gestation would be required to observe a 50% reduction in NEC with 80% power and α level of 0.05, not accounting for any drop-out. However, the actual NEC incidence in the present study did not differ much, 7.0% and 8.0% in the HMBF and BMBF group, respectively, which indicates that a much larger trial would be needed to prove any possible effect on NEC. We recognise that the incidence of the primary outcome in the present trial was lower than anticipated based on power calculations. However, as the differences in the primary outcome were minimal between the study groups, our result regarding the primary composite outcome is unlikely to be attributed to insufficient statistical power. However, we cannot exclude that a substantially larger trial potentially could find clinically relevant differences in major neonatal morbidities or mortality.

One strength of this study is the adherence to current consensus recommendations stating, also in accordance with clinical Swedish practise, that MOM is the first choice in the feeding of preterm infants and when mother's milk is not available, pasteurised donor human milk should be used.^11^ For this study, the latter was made realisable owing to a unique 100% coverage of donor milk banks in the Nordic countries. Furthermore, in line with current recommendations, individualised targeted fortification, utilising bedside human milk analysers, was used.^13^^,^^16^ For this reason the study group could not be masked, which is a limitation. However, “hard” outcomes like NEC, sepsis and mortality were less likely to be influenced by this, especially when study group was masked to an independent radiologist and a blinded consensus panel review.^25^ Still, there are outcomes, that potentially could have been affected by the unmasked design of the trial, such as feeding intolerance. The results of these outcomes should therefore be interpreted with caution. However, for measures of feeding intolerance, we observed no significant difference between groups which is in accordance with previous results.^18^^,^^23^ In the present study, supplementation with HMBF was started on median postnatal day 6 which is considerably earlier than in the trials by O'Connor et al. and Sullivan et al., where HMBF was introduced on mean postnatal day 17^23^ and 14,^17^^,^^18^ respectively. Hence, a lack of effect on primary and secondary outcomes could not be attributed to a delay in start of fortification. Neither could protocol violation or drop-out explain this, as per-protocol analysis did not change the results. Only very few infants (n = 10) received formula during the intervention period and at late PMW, thus, an influence on the results from this is unlikely. Fortification with BMBF in the HMBF group after the end of the intervention period could potentially influence outcomes with late onset after PMW 34 + 0. However, with no late events in the HMBF group (NEC, first sepsis episode, or death) this could not have influenced the result. Conventional fortifiers are powders mixed into the breast milk, while HMBF are in liquid form, substituting a part of the breastmilk and thereby potentially reducing the protective effects of MOM.

For a treatment course of one infant up to a PMW of 32 weeks, supplementation with HMBF has an estimated cost of €10,000 to €12,000 on the European market (Prolacta Bioscience, March 2023). A recent comparison of NICU costs found the overall cost of level three NICU care to be 1.55-fold higher with BMBF than HMBF.^32^ Nevertheless, based on previous studies, HMBF have been suggested to be cost-effective in preventing NEC.^33^ A future cost-effectiveness analysis was planned in the present trial, but it is not likely that HMBF will be shown cost-effective, as there was no effect on the primary or secondary outcomes.

We acknowledge that the results of the present trial may need to be verified by other studies. Further, potential effects on growth and neurodevelopmental outcomes still remain to be evaluated, and such follow-up analyses from this study are ongoing.^25^ Only extremely preterm infants were included in the present trial. Hence, we cannot exclude that the intervention may have an effect in less preterm infants. Our finding, that the HMBF group, when adjusted for gestational age, had decreased odds of having the secondary outcome BPD at PMW 36, should be interpreted with caution, although an effect on BPD has been reported previously in a retrospective observational study.^20^ Because efficacy and safety data are still limited,^13^ further studies on possible positive effects of HMBF are warranted.

In summary, we showed that supplementation with HMBF, as compared to BMBF, did not reduce the combined incidence of NEC, sepsis, or mortality in extremely preterm infants exclusively fed breast milk. Based on this and previous lack of evidence together with economical concerns with human milk-based fortifiers,^13^ we find no evidence to support the routine use of HMBF as a nutritional strategy to prevent NEC, sepsis, or death in extremely preterm infants who are fed own mother's milk or donor breast milk, and not preterm formula.

## Contributors

Coordinating principal investigator: TA. Conceptualisation: TA, FA, MD, AE. Formal analysis: TA, GBJ. Funding acquisition: TA. Methodology: TA, FA, MD, AE. Project administration (steering committee): TA, FA, GBJ, MD, AE, LN. Resources: TA, FA, MD, AE, LN. Supervision: TA. Validation: TA, FA, MD, AE, LN. Recruitment of patients: TA, FA, GBJ, MD, AE, LN. Statistical analysis and data tables: GBJ, TA. Writing—original draft: GBJ. Writing—interpretation of results, review, and editing: TA, FA, GBJ, MD, AE, LN. All authors had full access to all the data in the study and had final responsibility for the decision to submit for publication.

## Data sharing statement

Personal information about enrolled participants will be collected, shared, and maintained in accordance with the EU General Data Protection Regulation. Crown Princess Victoria Children's Hospital, County of Östergötland, Linköping, Sweden, is the sponsor and owns all the information obtained in the trial together with the coordinating principal investigator.

The study protocol (protocol version 2020/vers.4, March 25, 2020), statistical analysis plan, and informed consent form is available at the institutional webpage (URL: liu.se/en/research/n-forte). The information obtained during this study may be made available to other researchers who are conducting similar studies and to international or national medical authority, with due respect to the scientific priority of the investigation and after consulting the coordinating principal investigator.

## Declaration of interests

TA received a grant for the present study by Prolacta Bioscience, CA, USA. All other authors declare no competing interests. None of the investigators have any financial interest in Prolacta Bioscience.

## Appendix A Supplementary data

Supplementary Tables

Protocol
