# Supplementary material for: Effect of human milk-based fortification in extremely preterm infants fed exclusively with breast milk: a randomised controlled trial
Source: eClinicalMedicine. 2024 Jan 2;68:102375. doi: 10.1016/j.eclinm.2023.102375 (PMC10965410; doi:10.1016/j.eclinm.2023.102375)
Supplement: Supplementary Tables [file mmc1.pdf]

Supplementary tables

Table of contents

Table S1: Primary and selected secondary outcomes of the intention-to-treat population stratified by gestational age at birth 2

Table S2: Primary and selected secondary outcomes adjusted for gestational age 3

Table S3: Per-protocol analyses of primary and selected secondary outcomes 4

Table S4: Per-protocol analyses with adjustment for gestational age 5

Table S5: Primary and secondary outcomes adjusted for enteral intake at start of fortification 6

Table S1: Primary and selected secondary outcomes of the intention-to-treat population stratified by gestational age at birth

	HMBF (n=115)	BMBF (n=113)	p value*	p value†
Composite of necrotising enterocolitis (NEC), culture-proven sepsis and mortality	41/115 (35·7%)	39/113 (34·5%)	0·86	
GA 22	3/5 (60·0%)	5/6 (83·3%)	0·54	
GA 23	8/17 (47·1%)	7/10 (70·0%)	0·42	0·93
GA 24	10/16 (62·5%)	12/23 (52·2%)	0·52	
GA 25	6/23 (26·1%)	5/17 (29·4%)	1.00	
GA 26	11/32 (34·4%)	5/24 (20·8%)	0·27	
GA 27	3/22 (13·6%)	5/33 (15·2%)	1.00	
NEC II-III	8/115 (7·0%)	9/113 (8·0%)	0·77	
GA 22	2/5 (40·0%)	0/6 (0·0%)	0·18	
GA 23	3/17 (17·6%)	2/10 (20·0%)	1.00	
GA 24	2/16 (12·5%)	4/23 (17·4%)	1.00	0·96
GA 25	0/23 (0·0%)	1/17 (5·9%)	0·43	
GA 26	1/32 (3·1%)	0/24 (0·0%)	1.00	
GA 27	0/22 (0·0%)	2/33 (6·1%)	0·51	
Death	7/115 (6·1%)	13/113 (11·5%)	0·15	
GA 22	0/5 (0·0%)	4/6 (66·7%)	0·061	
GA 23	4/17 (23·5%)	1/10 (10·0%)	0·62	
GA 24	1/16 (6·3%)	6/23 (26·1%)	0·21	0·19
GA 25	1/23 (4,3%)	0/17 (0·0%)	1.00	
GA 26	1/32 (3·1%)	2/24 (8·3%)	0·57	
GA 27	0/22 (0·0%)	0/33 (0·0%)	NA	
Culture-proven sepsis	33/115 (28·7%)	28/113 (24·8%)	0·50	
GA 22	2/5 (40·0%)	4/6 (66·7%)	0·57	
GA 23	4/17 (23·5%)	6/10 (60·0%)	0·10	
GA 24	8/16 (50·0%)	7/23 (30·4%)	0·22	0·72
GA 25	6/23 (26·1%)	5/17 (29·4%)	1.00	
GA 26	10/32 (31·3%)	3/24 (12·5%)	0·10	
GA 27	3/22 (13·6%)	3/33 (9·1%)	0·67	
Bronchopulmonary dysplasia	60/108 (55·6%)	66/102 (64·7%)	0·18	
GA 22	4/5 (80·0%)	2/2 (100·0%)	1.00	
GA 23	12/13 (92·3%)	8/9 (88·9%)	1.00	
GA 24	13/15 (86·7%)	15/19 (78·9%)	0·67	0·070
GA 25	14/22 (63·6%)	12/17 (70·6%)	0·65	
GA 26	13/31 (41·9%)	14/23 (60·9%)	0·17	
GA 27	4/22 (18·2%)	15/32 (46·9%)	0·030	
Retinopathy of prematurity, stage III-V	29/113 (25·7%)	25/110 (22·7%)	0·61	
GA 22	3/5 (60·0%)	2/6 (33·3%)	0·57	
GA 23	8/16 (50·0%)	5/9 (55·6%)	1.00	
GA 24	10/16 (62·5%)	10/22 (45·5%)	0·34	0·76
GA 25	4/22 (18·2%)	3/17 (17·6%)	1.00	
GA 26	4/32 (12·5%)	2/24 (8·3%)	0·69	
GA 27	0/22 (0·0%)	3/32 (9·4%)	0·26	

Data are n/N (%). Outcome numbers are stratified by gestational age (week) at birth. For each gestational week the numbers within that particular gestational age group are presented. Only events after time of inclusion. HMBF=human milk-based fortifier. BMBF=bovine milk-based fortifier. NEC=necrotising enterocolitis. GA=gestational age (week). *The chi-square test (or Fisher's exact test if the expected count was less than five) was used to compare frequencies. †The Cochran-Mantel-Haenszel test was used for overall comparison considering the stratification by gestational age.

Table S2: Primary and selected secondary outcomes adjusted for gestational age

	Crude OR	p value	Adjusted OR*	p value
Composite of necrotising enterocolitis (NEC), culture-proven sepsis, and mortality	1·05 (0·61-1·81)	0·86	0·97 (0·54-1·73)	0·92
NEC II-III	0·86 (0·32-2·33)	0·77	0·78 (0·29-2·16)	0·63
NEC, surgical	0·98 (0·24-4·03)	0·98	0·90 (0·22-3·75)	0·89
Death	0·50 (0·19-1·30)	0·16	0·41 (0·15-1·12)	0·081
Culture-proven sepsis	1·22 (0·68-2·20)	0·50	1·16 (0·63-2·13)	0·63
Bronchopulmonary dysplasia	0·68 (0·39-1·19)	0·18	0·54 (0·29-1·00)	0·049
Retinopathy of prematurity stage III-V	1·17 (0·64-2·17)	0·61	1·07 (0·55-2·10)	0·84
Mortality and morbidity index†	0·69 (0·39-1·24)	0·22	0·59 (0·31-1·10)	0·10

Data are crude or adjusted OR with 95% CI for human milk-based fortifier (HMBF) group. Intention-to-treat. Only events after time of inclusion are presented. NEC=necrotising enterocolitis. *Logistic regression. Adjustment was done for gestational age. †Composite requiring any of the following: death, NEC stage II–III, culture-proven sepsis, bronchopulmonary dysplasia or retinopathy of prematurity stage III–V.

Table S3: Per-protocol analyses of primary and selected secondary outcomes

	HMBF (n=108)	BMBF (n=97)	p value*
**Primary outcome**			
Composite of necrotising enterocolitis (NEC), culture-proven sepsis, and mortality	29 (26·1%)	29 (27·9%)	0·63
**Secondary outcomes**			
Clinical variables for morbidity			
NEC II-III	8 (7·2%)	6 (5·8%)	0·73
NEC, surgical	4 (3·6%)	2 (2·1%)	0·49
Death	4 (3·7%)	8 (8·2%)	0·17
Culture-proven sepsis	24 (22·2%)	22 (22·7%)	0·94
Composite of NEC and culture-proven sepsis	29 (26·1%)	27 (26·0%)	0·88
Suspected sepsis, not culture-proven	30 (27·8%)	31 (32·0%)	0·51
Culture-proven or suspected sepsis	47 (42·3%)	43 (41·3%)	0·91
Bronchopulmonary dysplasia	59/104 (56·7%)	61/92 (66·3%)	0·17
Retinopathy of prematurity	49/106 (46·2%)	40/96 (41·7%)	0·51
Retinopathy of prematurity, stage III-V	28/106 (26·4%)	21/96 (21·9%)	0·45
Mortality and morbidity index†	71 (64·0%)	72 (71·2%)	0·19
Weight at PMW 34+0 (g)	2000 (1777-2190)	1895 (1719-2096)	0·055
Postmenstrual age at discharge (weeks)‡	41·4 (38·8-44·0)	41·4 (38·9-44·0)	0·75
Feeding intolerance			
Feeding interruption ≥12 hours	33 (30·6%)	35 (36·1%)	0·40
Feeding reduced >50%	46 (42·6%)	37 (38·1%)	0·52
Feeding interrupted or reduced	47 (43·5%)	42 (43·3%)	0·98
Gastric aspirates ≥100% of prefeed volume	47 (43·5%)	44 (45·4%)	0·79
Stool frequency (stools/day)	3·5 (1·0)	3·5 (0·80)	0·99
Time to reach full enteral feeds, 150 mL/kg/d (days)§	10 (8-15)	10 (8-13)	0·43
Time to reach full enteral feeds, first of three days (days)§	10 (8-16)	10 (8-14)	0·46

Data are n (%), n/N (%), or median (IQR). Per-protocol analyses only considering events with an onset from the first day of fortification and also excluding infants with protocol violation (withdrawal of consent before postmenstrual week (PMW) 34+0, formula before PMW 34+0, lost to follow-up) or infants that did not receive the study product. Feeding intolerance measures were registered until discharge (no longer than PMW 44+0). HMBF=human milk-based fortifier. BMBF=bovine milk-based fortifier. NEC=necrotising enterocolitis. *The chi-square test (or Fisher's exact test if the expected count was less than five) to compare frequencies, and the non-parametric Mann-Whitney test to compare distributions †Composite requiring any of the following: death, NEC stage II–III, culture-proven sepsis, bronchopulmonary dysplasia or retinopathy of prematurity stage III–V. ‡PMW 44+0 at latest; survivors only §Only if full enteral was reached.

Table S4: Per-protocol analyses with adjustment for gestational age

	Crude OR	p value	Adjusted OR*	p value
Composite of necrotising enterocolitis (NEC), culture-proven sepsis, and mortality	0·86 (0·47-1·58)	0·63	0·78 (0·41-1·46)	0·44
NEC II-III	1·21 (0·41-3·63)	0·73	1·09 (0·36-3·34)	0·88
NEC, surgical	1·83 (0·33-10·20)	0·49	1·69 (0·30-9·51)	0·55
Death	0·43 (0·13-1·47)	0·18	0·34 (0·094-1·21)	0·10
Culture-proven sepsis	0·97 (0·51-1·88)	0·94	0·91 (0·46-1·78)	0·78
Bronchopulmonary dysplasia	0·67 (0·37-1·19)	0·17	0·52 (0·27-0·98)	0·044
Retinopathy of prematurity, stage III-V	1·28 (0·67-2·45)	0·45	1·13 (0·55-2·35)	0·74
Mortality and morbidity index†	0·67 (0·36-1·22)	0·19	0·55 (0·28-1·05)	0·070

Data are crude or adjusted OR with 95% CI for human milk-based fortifier (HMBF) group. Per-protocol analyses only considering events with an onset from the first day of fortification and also excluding infants with protocol violation (withdrawal of consent before postmenstrual week (PMW) 34+0, formula before PMW 34+0) or infants that did not receive study product. NEC=necrotising enterocolitis. *Logistic regression. Adjustment was done for gestational age. †Composite requiring any of the following: death, NEC stage II–III, culture-proven sepsis, bronchopulmonary dysplasia or retinopathy of prematurity stage III–V.

Table S5: Primary and secondary outcomes adjusted for enteral intake at start of fortification

	Crude OR	p value	Adjusted OR*	p value
Composite of necrotising enterocolitis (NEC), culture-proven sepsis, and mortality	1·20 (0·68-2·13)	0·52	1·12 (0·62-2·01)	0·71
NEC II-III	1·29 (0·43-3·86)	0·64	1·23 (0·40-3·79)	0·72
NEC, surgical	1·94 (0·35-10·84)	0·45	1·84 (0·31-10·98)	0·50
Death	0·46 (0·13-1·57)	0·21	0·43 (0·12-1·50)	0·19
Culture-proven sepsis	1·37 (0·75-2·51)	0·31	1·28 (0·69-2·38)	0·44
Bronchopulmonary dysplasia	0·72 (0·41-1·26)	0·25	0·81 (0·46-1·44)	0·48
Retinopathy of prematurity, stage III-V	1·21 (0·65-2·25)	0·55	1·30 (0·69-2·48)	0·42
Mortality and morbidity index†	0·75 (0·42-1·35)	0·33	0·83 (0·45-1·50)	0·53

Data are crude or adjusted OR with 95% CI for human milk-based fortifier (HMBF) group. Only events after time of inclusion are presented. Per-protocol, infants receiving no fortification were excluded. NEC=necrotising enterocolitis. *Logistic regression. Adjustment was done for enteral intake (mL/kg/day) at start of fortification. †Composite requiring any of the following: death, NEC stage II–III, culture-proven sepsis, bronchopulmonary dysplasia or retinopathy of prematurity stage III–V.
