# Supplementary material for: Effect of human milk-based fortification in extremely preterm infants fed exclusively with breast milk: a randomised controlled trial
Source: eClinicalMedicine. 2024 Jan 2;68:102375. doi: 10.1016/j.eclinm.2023.102375 (PMC10965410; doi:10.1016/j.eclinm.2023.102375)
Supplement: Protocol [file mmc2.pdf]

| | |
|---|---|
| **STUDY TITLE:** | **Nordic study on human milk fortification in extremely preterm infants: a randomized controlled trial** |
| **ACRONYM:** | **N-forte** |
| **PROTOCOL N$^{o}$:** | **2020/vers.4** |
| **DATE OF PROTOCOL** | **2020-03-25** |
| **STUDY PRODUCT** | **Human milk-based human milk fortifier** ($H^2$ MF) |
| **PRINCIPAL INVESTIGATOR** | Associate Professor Thomas Abrahamsson, MD, PhD, Neonatologist. |
| **INVESTIGATORS** | Professor Magnus Domellöf. MD, PhD, Neonatologist<br>Associate Professor Anders Elfvin, MD, PhD, Neonatologist<br>Associate Professor Fredrik Ahlsson, MD, PhD, Neonatologist<br>Associate Professor Lars Naver, MD, PhD, Neonatologist |
| **CO-INVESTIGATORS** | Dr Ingela Danielsson, MD, Neonatologist<br>Georg Bach Jensen, MD, Pediatrician |
| **STUDY COORDINATOR** | Gisela Hellgren, Fravil Clinical Consulting, Sweden |
| **SPONSOR** | Thomas Abrahamsson, MD, PhD<br>H.K.H Kronprinsessan Victorias Barn- och ungdomssjukhus, plan 15<br>Universitetssjukhuset<br>Region Östergötland<br>581 85 Linköping<br>tel: 010 – 103 00 00 (vx)<br>mob: 0709 – 56 68 15<br>email: thomas.abrahamson@regionostergotland.se |

# Table of contents

Table of contents ........ 2
1 Summary ........ 6
1.1 Rationale ........ 6
1.2 Hypothesis ........ 6
1.3 Objective ........ 6
1.4 Study design ........ 7
2 Flow chart ........ 8
3 Background ........ 9
4. Objectives ........ 11
4.1 Hypothesis ........ 11
5. Research questions ........ 12
5.1 Primary research questions ........ 12
5.2 Secondary research questions ........ 12
5.3 Efficacy variables ........ 12
5.3.1 Primary variable ........ 12
5.3.2 Secondary variables ........ 12
5.3.2.1 Food intolerance and growth ........ 12
5.3.2.2 Clinical variables for morbidity ........ 13
5.3.2.3 Laboratory variables ........ 15
5.3.2.4 Health economics ........ 16
5.3.3 Associated predictor variables (covariates) ........ 16
6 Study design ........ 17
6.1 Design ........ 17
6.2 Selection of study design ........ 18
6.3 Selection of treatment ........ 18
7. Study population ........ 19
7.1 Sample size ........ 19
7.2 Inclusion criteria ........ 20
7.3 Exclusion criteria ........ 20
7.4 Withdrawal criteria ........ 21
7.5 Study termination ........ 21
8 Study period ........ 22
9 Methods ........ 22
9.1 Practical arrangements ........ 22
9.1.1 Inclusion and randomisation ........ 22
9.1.2 Twins ........ 23
9.1.3 Investigator file ........ 23
9.1.4 Start of the intervention ........ 23
9.1.5 Prescription, preparation and administration of the study product ........ 24
9.1.6 Case report form (CRF) and Nutrium ........ 25
9.1.7 Samples at 7 ± 2 days of age ........ 25
9.1.8 Samples at 14 ± 2 days of age ........ 25

9.1.9 Samples at 21 ± 2 days of age ... 25
9.1.10 Samples at 28 ± 2 days of age ... 26
9.1.11 The end of the study intervention, gw 34+0 ... 26
9.1.12 Transfer to another neonatal unit ... 27
9.1.13 Sample at gestational week 33+0-33+6 ... 27
9.1.14 Magnetic resonance imaging (MRI) ... 27
9.1.15 The end of the neonatal period ... 27
9.1.16 Follow up at 2 years of age ... 28
9.1.17 Follow up at 5.5 years of age ... 28
9.1.18 Deaths ... 28
*9.2 Assessment of efficacy ... 28*
9.2.1 Clinical variables ... 28
9.2.2 Sample collection ... 28
Blood samples ... 28
Faecal samples ... 29
Urine samples ... 29
Breast milk samples ... 29
*9.3 Assessment of safety ... 29*
9.3.1 Adverse events ... 29

**10. Concomitant therapy ... 29**

**11. Concomitant diseases ... 29**

**12. Study requirements ... 29**
*12.1 Study product ... 29*
*12.2 Dose, administration, storage ... 30*
12.2.1 Active study group ... 30
12.2.2 Control study group ... 30
*12.3 Storage ... 30*
*12.4 Study product accountability ... 30*
*12.5 Randomisation ... 30*

**13. Adverse Events ... 31**
*13.1 Definitions ... 31*
13.1.1 Non-serious adverse events ... 31
13.1.2 Serious adverse events ... 31
13.1.3 Suspected unexpected serious adverse reaction (SUSAR) ... 32
*13.2 Reporting serious adverse events ... 32*
13.2.1 Reporting SAE ... 32
13.2.2 Reporting lethal cases and SUSAR ... 32
*13.3 Interim analysis ... 32*
*13.4 Data and safety monitoring board (DSMB) ... 33*

**14 Data collection ... 33**
*14.1 Case Report Form (CRF) ... 33*
*14.2 Data source ... 34*

**15 Data quality assurance ... 34**
*15.1. Study monitoring ... 34*
*15.2. Study monitoring ... 34*

**16 Ethics ... 34**

**17. Informed consent** ..... 35

**18. Approval from research ethical board and other authorities** ..... 36

**19. Study discontinuation** ..... 36

**20. Statistical methods** ..... 36

**21. Financing and insurance** ..... 37

**22. Final study report** ..... 37

**23. Retention of records** ..... 37

**24. Confidentiality and publication** ..... 38

**25. References** ..... 38

**25. Appendix** ..... 41

*Appendix 1 –Agreement between sponsor and investigator* ..... 41
*Appendix 2 –List of contributing neonatal units and their contact persons* ..... 42
*Appendix 3 –Agreement with the head of the clinic (this was included in the ethical application)* ..... 52
*Appendix 4 – Data and safety monitoring board (DSMB)* ..... 53
*Appendix 5 –Reporting of SUSAR* ..... 54
*Appendix 6 –Study information to the parents* ..... 55
*Appendix 7 –Informed consent form* ..... 58
*Appendix 8 –Case report form* ..... 59
*Appendix 9 –Prescription and preparation of the study product* ..... 60
*Appendix 10 – Transition to discharge feeding regimen* ..... 63
*Appendix 11 –Classification of level of care* ..... 65
*Appendix 12 –Blood sample: instructions* ..... 66
*Appendix 13 –Stool sample: instructions* ..... 67
*Appendix 14 –Urine sample: instructions* ..... 68
*Appendix 15 –Breast milk sample: instructions* ..... 69
*Appendix 16 –Nutritional content of $H^2$ MF and Prolact CR* ..... 70
*Appendix 17 –Product description and safety information* ..... 71

# List of abbreviations:

AE= Adverse Event

BPD=Bronchopulmonary dysplasia

CRF= Case Report Form

CRO=Contract research organisation

CPAP=Continuous positive airway pressure

DSMB=Data and Safety Monitoring Board

EHM=Exclusively human milk

ELBW=Extremely low birth weight

ELGA=Extremely low gestational age

GCP= Good Clinical Practice

ICH= International Conference on Harmonisation

IVH= Intraventricular haemorrhage

ITT= Intention-to-treat

NEC=Necrotizing enterocolitis

NICU=Neonatal Intensive Care Unit

PDA=Patent ductus arteriosus

PPA= Per Protocol analysis

PPROM=Preterm premature rupture of the membrane

PVL= Periventricular leucomalacia

REB=Research Ethic Board

SAE= Serious Adverse Event

SCFA=Short Chain Fatty Acids

SIP=Spontaneous intestinal perforation

SUSAR= Suspected Unexpected Serious Adverse Reaction

# 1 Summary

## 1.1 Rationale

Although the care of premature infants has improved dramatically during the last decades, still about 30% of the extremely low gestational age (ELGA born < gestational week 28+0) infants die – and severe infections and necrotizing enterocolitis (NEC) are common causes of death. There are clear links between nutrition and the risk of NEC, infection, neurological sequelae and mortality. A common cause of poor nutrition in this patient group is poor enteral feed tolerance due to immaturity and dysfunction of gastrointestinal tract – and thus ELGA infants usually require parenteral feeding though intravenous catheters that increase the risk of sepsis. Nutritional problems and their consequences are a major problem in neonatal care.

However, choice of diet has a major impact on morbidity and clinical course. Preterm infants receiving human milk have better feeding tolerance and lower incidence of NEC than those fed cow milk based preterm formula. Fortunately, because of the widespread availability of donor milk banks in Nordic countries, ELGA infants are generally receiving a base diet of human milk without the need for cow's milk-based formulas. Despite this, the incidence of NEC in ELGA infants is still as high as 12% according to the Swedish National Quality Register (www.snq.se). We hypothesise that a key reason for this is that even fully human milk fed ELGA infants have a significant intake of cow's milk protein. This is because human milk alone does not fully meet the nutritional requirements in ELGA infants, but needs to be supplemented with a nutrient fortifier – and such fortifiers available in Nordic Countries are based on cow's milk. Indeed, because breast milk protein levels are so low in relation to the needs of ELGA infants, such fortifiers often provide more cow's milk protein than the infant receives from an all-human milk diet.

$H^2MF^®$ (Prolacta Bioscience, CA, USA) is a human milk-based nutrient fortifier for preterm infants. Previous trials evaluating Prolacta fortifiers in extremely preterm infants receiving exclusive human milk diet suggest that exclusive human milk diet, devoid of cow milk-containing products, is associated with lower mortality and morbidity in these infants without compromising growth. Thus, such an exclusive human milk diet has been reported to reduce the incidence of NEC, sepsis, bronchopulmonary dysplasia (BPD) and retinopathy of the prematurity (ROP). No side effects have been reported. Still, there is no well powered trial in ELGA infants comparing a human milk-based fortifier with bovine protein-based fortifier in which both study groups receive an all-human breast milk-based diet. Such a trial is needed to provide evidence that a human milk-based fortifier is superior to bovine-based one in ELBW infants also in centres with 100% coverage of breast milk donor banks. Moreover, due to the introduction of active intervention for infants also born before gestational week 25 during the last decade, a trial run in Nordic centres will have a predominance of infants born in gestational week 23-25, a patient population that could be expected to gain the most of a diet free from bovine protein. A significant positive result of this trial could easily be implemented in the NICUs, since Prolact $H^2MF^®$ already exists and has been used extensively - by around 1 in 4 level 3 NICUs in the US.

## 1.2 Hypothesis

Extremely preterm infants exclusively fed with human breast milk (mother´s own milk and/or donor milk) supplemented with the human milk-based nutrient fortifier will have a lower incidence of severe complications: NEC, sepsis and mortality (as a composite) than ELGA infants supplemented with a bovine protein-based one.

## 1.3 Objective

To evaluate if diet supplementation with the human milk-based nutrient fortifier $H^2MF^®$ reduces severe complications - NEC, sepsis, mortality - and improves enteral feeding tolerance as compared with bovine protein-based nutrient fortifier in extremely preterm infants receiving exclusive human breast milk (own mother´s milk

and/or donor milk). Possible mechanisms underlying the effect of H²MF® will be analysed in blood, urine and stool samples. The health economic impact of an implementation will also be evaluated.

## *1.4 Study design*

This is a randomised controlled multi-centre trial comparing the effect of diet supplementation of a human breast milk-based nutrient fortifier (H²MF®) with standard bovine protein-based nutrient fortifier in 222 extremely preterm infants (born before gestational week 28+0) exclusively fed with human breast milk (own mother´s milk and/or donor milk). The infants will be randomised to receive either the human breast-milk based H²MF® or the standard bovine protein-based nutrient fortifier when oral feeds have reached <100 ml/kg/day. If fortification with extra enteral lipids is needed during the intervention period, the infants receiving H²MF® will be supplemented with the human milk-based Prolact CR®, while the infants receiving standard bovine protein-based fortification will be supplemented with the standard lipid products used at the unit. The study subject will be enrolled at level III NICUs. Only infants with a home clinic with the logistics to maintain the intervention until gestational week 34+0 will be included.

The randomised intervention, stratified by centre, will continue until the target gestational week 34+0. The infant must not be fed with formula during the intervention period. The allocation will be concealed before inclusion, but after randomisation the study is not blinded. It would not be possible to prescribe the fortifier and prepare of the breast milk in a blinded fashion, since the fortifiers are not exactly equal in nutrient content and also look different. Instead the assessment of several of the outcomes will be made blinded, such as the assessment of X-ray images in NEC cases.

The enrolled infants are characterised with clinical data including growth, feeding intolerance, use of enteral and parenteral nutrition, treatment, antibiotics and complications collected daily in a study specific case report form from birth until discharge from the hospital (not longer than gestational week 44+0). A follow up focusing on neurological development, growth and feeding problems will be performed at 2 years of age (corrected) and 5.5 years of age.
Since it is often difficult to distinguish between the diagnoses of NEC and sepsis, and their clinical consequences, our primary endpoint of the intervention is the composite variable NEC, sepsis and mortality. Secondary endpoints are feeding intolerance and other severe complication such as BPD, ROP and neurological impairment. Stool, urine and blood samples are also collected for microbiology, metabolomic and immunology analysis in order to study underlying mechanisms. Health economic analyses will be made to evaluate the costs and benefits of an introduction of human milk-based fortifier in NICUs in the Nordic countries.

Analyses will be conducted using an intention to treat approach. An evaluation will be performed when 20 infants have been included to evaluate feasibility and make it possible to adjust the protocol for the remaining part of the study. Safety analyses will be performed by an independent data and safety monitoring board (DSMB) when 50, 100 and 150 infants have been included. A sample size re-estimation will be made by an independent statistician when 150 infants have been included. Thus, the definitive sample size might be increased (never decreased) based on this interim analysis. The study can be terminated before 222 infants have been enrolled based on a decision of the sponsor and the DSMB, if the primary outcome is significantly lower (with a significance level <0.001) in the H²MF® than in the standard fortification group in the interim analysis made after 150 infants have completed the neonatal period. The study subject will be enrolled at level III NICUs in the Nordic Countries. All study subjects will be followed during the neonatal period until discharge (not longer than gestational week 44+0) and also be included in a follow up at 2 and 5.5 years of age based on the national follow up program for extremely preterm infants.

# 2 Flow chart

| ***Time point*** | **Inclusion** | | | | | **Inter-vention ends** | | **Neonatal follow up ends** | **Follow up at age of** **2y** | **5.5y** |
|---|---|---|---|---|---|---|---|---|---|---|
| ***Day of life*** | **<100 ml/kg/d** | **7 d ± 2 d** | **14 d ± 2 d** | **21 d ± 2 d** | **28 d ± 2 d** | | | | | |
| ***Gestational week*** | | | | | | **-w34+0** | **w36+0** | **-w44+0** | | |
| Informed consent | X | | | | | | | | | |
| Incl.-/exclusion criteria | X | | | | | | | | | |
| Background history | X | | | | | | | | | |
| Randomisation | X | | | | | | | | | |
| Intervention | \|\|========================================\|\| | | | | | | | | | |
| Feeding intolerance | \|\|========================================\|\| | | | | | | | | | |
| NEC, sepsis, mortality | | \|\|=================================================\|\| | | | | | | | | |
| Neurological impairment* | | | | | | | | | X | X |
| Antibiotics and concomitant medication | X | X | X | X | X | w34+0 | X | X | | |
| Weight** | | X | X | X | X | w34+0 | X | X | X | X |
| Height/head circumf.** | | 7d | 14d | 21d | 28d | w34+0 | X | X | X | X |
| Blood sample (if available) | | X | X | | X | w33+0-6 | | | | |
| Urine sample (if available) | | X | X | X | X | w33+0-6 | | | | |
| Stool sample (if available) | | X | X | X | X | w33+0-6 | | | | |
| Breast milk (if available) | | X | X | X | X | w33+0-6 | | | | |
| Adverse Events | X | X | X | X | X | X | X | X | | |

*=Including examination by paediatrician and psychologist (incl. Bayley III test)
**= In the table it is only notified when measurements must be made in the trial (weight is often measured every day).

# 3 Background

Although the care of premature infants has improved dramatically during the last decades, the extremely low birth weight (ELBW, birth weight <1000g) preterm infant remains a major challenge for neonatal intensive care.
About 30% of these babies die [1] and the survivors may have a complicated clinical course with major morbidity and a significant risk of impaired later outcome.
Nutrition is increasingly recognised as a key factor in the survival, clinical course and later outcome of high-risk preterm infants; and of particular relevance to proposed project is the broad evidence for beneficial roles of human milk in neonatal care, underpinning current recommendations for its use [2].

Extremely preterm infants are usually fed parenterally while enteral feed tolerance develops, though even when feeds are tolerated, these infants are too immature to be fed orally and require tube feeding. Human milk, fed by tube, is generally expressed milk from the infant's own mother. However, in Nordic countries, donor breast milk is usually available when mothers provide no or insufficient breast milk. A growing literature supports the clinical value of using human milk for feeding preterm infants, in particular for the smallest and most vulnerable premature infants. Proposed benefits of human milk for such infants are considered as follows.

*Reduction of necrotising enterocolitis (NEC)*
NEC remains a significant problem in neonatal care with an incidence in some centres in the US in ELGA infants of close to 20% [3] and around 12% in Sweden (www.snq.se), where the peak incidence is 16% for infants born at gestational week 24. NEC frequently results in death (around 20-30%) or the requirement for surgical resection of necrotic gut which may be sufficiently extensive to necessitate long term parenteral nutrition in hospital and subsequently at home. It is increasingly recognised that NEC is a major adverse factor for subsequent lower IQ, motor impairment, visual impairment and cerebral palsy [4]. The health economic cost of NEC is large [5,6].
A number of earlier observational studies including those by Lucas and Cole [7] in 1990, El-Mohandes *et al* in 1997 [8] were followed by a meta-analysis by Boyd *et al* in 2007, concluding that human milk feeding was associated with a reduced incidence of NEC [9]. More extensive recent evidence comes from two randomised trials [10,11] and 8 observational studies [3,12] [13] in the US, collectively involving over 4000 preterm infants under 1250g birth weight. These studies, show on average over 70% reduction in NEC with the use of an exclusive human milk diet achieved by feeding human-milk based products.

*Reduction in neonatal sepsis*
Neonatal sepsis remains a life-threatening and common problem in neonatal care. The incidence is as high as 35-40% in ELGA infants (www.snq.se). Growing evidence links sepsis to the mode of feeding. Thus, early observational studies by Naraynan *et al* [14-16] and Lucas *et al* [17] showed human milk was associated with less sepsis. Further evidence comes from a randomised trial of breast milk fortification in which Lucas et al showed that those who received a cow's milk-based breast milk fortifier had a significant increase in the combined outcome of NEC or proven sepsis. Each 10% increase in feed containing cow´s milk was associated with an 18% increase in sepsis [18]. Abrams *et al* also showed a dose related reduction in proven sepsis with increasing human milk usage [19]. Hair *et al* showed in a four centre, 1600 preterm infant observational study that introduction of an exclusive human milk diet was followed by a highly significant reduction in sepsis from 30% to 19% [3].

*Feed tolerance and reduced need for parenteral nutrition*
Poor feed tolerance is a significant and pervasive problem in the management of ELGA infants that poses the risk of under-nutrition, which not only undesirably prolongs he use of parental nutrition, but also may compromise optimal neurodevelopment [20]. Several studies have shown that use of human milk is associated with better feed tolerance, reduced time to full enteral feeds and reduced need for parenteral nutrition [11,12]. Parenteral nutrition in

ELGA infants, though necessary from a nutritional perspective, is associated with potential clinical hazards, including systemic sepsis related to long lines required.

*Long-term impact of human milk in neonatal care*

As preterm infants are being followed increasingly throughout childhood and into adult life, long-term effects of breast milk in neonatal care are emerging. A beneficial effect of prior randomisation to an exclusive human milk diet has been shown in preterm-born 16-year old children. They had favourably reduced LDL/HDL cholesterol ratio, diastolic blood pressure, insulin resistance and metabolic tendency to fatness - key risk factors for cardiovascular disease [21-24]. Larger bone mass in adolescence [25], higher cognitive performance in childhood [26,27] and increased brain size and white matter in adolescence [28] have also been reported in those fed human milk as neonates. A recent observational study showed a reduction in all classes of retinopathy of prematurity in ELBW infants from 9% with cow's milk products in the diet, down to 5% on an exclusive human milk diet [3]. Moreover, preterm-born adults aged 25-28 years who were fed exclusive human milk in infancy have more normal sized cardiac ventricles and stroke volumes, whereas these were markedly reduced in those fed exclusively formula in the neonatal period [29].

*Nutritional inadequacy of breast milk for preterms: implications*

It is well recognised that despite the health benefits of human milk considered above, it does not meet the nutritional needs of ELBW infants whose rate of somatic and brain growth far exceeds that in full term neonates. In order to meet the nutritional needs of enterally fed preterm infants additional products are therefore required and these are generally cows-milk based. For infants whose mothers provide no or inadequate volumes of expressed breast milk and where there is no access to milk bank facilities, these infants require a cow's milk based preterm formula. But even in Nordic countries where a 100% human milk base diet is achievable through the use of donor milk alongside mother's milk, all the breast milk used needs to be fortified – again with a cows-milk based fortifier.

Whilst cow's milk products are used in modern neonatal care to provide adequate nutrition – for instance to fuel brain growth – current evidence, outlined above, indicates that cow's milk products may have detrimental effects when compared to a human milk diet.

*The development of lacto-engineering*

The possibility of meeting nutritional needs of preterm infants whilst avoiding use of cow's milk products was first explored in 1980 by Lucas *et al* [30], who showed it was possible to develop a prototype formula for premature infants made only from human milk components. This was achieved through adding freeze-dried human milk powder and human milk cream (as an energy source) to whole human milk.

More recently, Prolacta Bioscience (CA, USA) has developed a series of human milk preparations that provide neonatologists with the first commercially available lacto-engineered products – preterm formulas and fortifiers - made from 100% human milk that have carefully controlled amounts of protein, carbohydrates and fat, as well as minerals and vitamins.

Pasteurized human milk products that are made from donor human milk have the theoretical potential for transmitting infectious diseases such as HIV or hepatitis. Prolacta Bioscience incorporates a number of steps to reduce the possibility of infectious disease transmission (please see appendix 17). These include careful donor screening, blood testing of the donor, and testing of the milk pre and post pasteurization by culture and PCR. Nevertheless, whilst the transmission of disease remains a remote possibility there are no reports of any disease transmission or any other adverse events to date, despite the products under study here have been used in more than 100,000 babies in around 400 centres across the United States and a few in Canada and Austria

*Clinical studies on human milk-based products*
Two randomised trials have examined the impact of these human milk-based formulations. The first, by Sullivan *et al* examined in 207 infants the impact of replacing cow's milk based preterm formulas and fortifiers with the human milk equivalents to produce an exclusively human (EHM) based diet milk [10]. Those fed the EHM diet showed a major reduction of NEC rate from 15.9% to 5.4% and a much larger reduction in NEC surgery rate from 11.6% to only 1.4%. In the second, smaller trial on the rare group of babies whose mothers provided no breast milk, randomisation was to a cow's milk based preterm formula as sole enteral feed versus exclusive human milk based products [11]. In the EHM group NEC surgery rate was zero compared to 17% in the preterm formula group. A study combining these two trials further showed a favourable impact of an exclusive human milk diet on sepsis risk and importantly a significant drop in death rate from 8% in those exposed to cow's milk products to only 2% in those on an EHM diet [19].

Since those trials, 8 investigator-led studies have examined during the period 2006-2014 the clinical impact of introducing an EHM diet compared to baseline morbidity [3,12 13]. Collectively these studies on around 4000 subjects principally under 1250g birth weight showed a 75% reduction in NEC. The studies provided further evidence for a major adverse impact of cow's milk based products on sepsis, mortality and retinopathy of prematurity, the latter now shown in 3 independent studies [3,31,32].

*In conclusion*
A growing literature supports the use of human milk-based products in clinical practise. The previous trials in the US, however, do not distinguish between the effects of replacing a cow's milk based preterm formula with a human milk-based equivalent and those replacing a cow's milk-based human milk fortifier with a human milk based one – in the main trial done in US [10] both were replaced. Thus, there is still no trial in ELBW infants comparing human milk-based nutrient fortifier with bovine protein-based one in which both study groups receive exclusive human breast milk. Moreover, due to the introduction of active interventions for infants also born before gestational week 25 during the last decade, a trial run in Nordic centres will have a predominance of infants born in gestational week 23-25, a patient population that could be expected to gain the most of a diet free from bovine protein. A significant positive result of this trial could easily be implemented in the NICUs, since the human milk-based nutrient fortifiers already exist as a product on the market and have been used and tested extensively.

# 4. Objectives

The primary objective is to evaluate if the supplementation of a human milk-based nutrient fortifier H²MF® reduces the severe complications of NEC, sepsis and mortality as compared with bovine protein-based nutrient fortifier in extremely preterm infants receiving exclusive human breast milk (own mother´s milk and/or donor milk). Secondary objectives are to evaluate if H²MF® supplementation improves enteral feeding tolerance and reduces other severe complications such as BPD, ROP and neurological impairment. Possible mechanisms underlying the effect of H²MF® will be analysed in blood, stool, urine and breast milk samples. Health economic analyses will be made to evaluate the costs and benefits of an introduction of human milk-based fortifier in NICUs in the Nordic countries.

## *4.1 Hypothesis*

The primary hypothesis is that extremely preterm infants exclusively fed with human breast milk (mother's own milk and/or donor milk) will have a lower combined incidence of severe complications - NEC, sepsis and

mortality – when supplemented with human milk-based nutrient fortifier $H^2MF^®$ as compared to supplementation with a bovine protein-based one'.

# 5. Research questions

## 5.1 Primary research questions

Does supplementation with the human milk-based nutrient fortifier $H^2MF^®$ reduce NEC, culture-proven sepsis and mortality in extremely preterm infants?

## 5.2 Secondary research questions

- Does supplementation with the human milk-based nutrient fortifier $H^2MF^®$ reduce the composite of NEC and culture-proven sepsis in extremely preterm infants?
- Does supplementation with the human milk-based nutrient fortifier reduce feeding intolerance in extremely preterm infants?
- Does supplementation with the human milk-based nutrient fortifier improve growth and the neurological development in extremely preterm infants?
- Does supplementation with the human milk-based nutrient fortifier reduce other severe complications such as BPD, ROP in extremely preterm infants?
- Does supplementation with human milk-based nutrient fortifier reduce the days of intensive care needed and the length of stay at the hospital?
- Does the use of human milk-based nutrient fortifier reduce total cost of neonatal care according to health economic analyses?
- What are the immunological, microbiological and metabolic mechanisms underlying the effect of a human milk-based nutrient fortifier in extremely preterm infants?
- What are the immunological, microbiological and metabolic mechanisms underlying feeding intolerance, growth restriction, neurological impairment, NEC, sepsis and bronchopulmonary dysplasia?

## 5.3 Efficacy variables

### 5.3.1 Primary variable

The composite of NEC, culture-proven sepsis and mortality: An infant should have had any of these diagnoses to fulfil the criterion. Please see the definition of NEC and sepsis below.

.

### 5.3.2 Secondary variables

#### 5.3.2.1 Food intolerance and growth

1. Time to reach full enteral feeds: the day of life the infant has received at least 150 mL/kg enteral feeds.
2. Feeding interruption: number of days feedings held for ≥12 hours OR feeds reduced by >50% (ml/kg/d) not due to a clinical procedure or transitioning to the breast.
3. Parenteral nutrition: number of days of parental amino acid and/or lipid infusion. Only days when the enteral feed <150mL/kg/day should be included.
4. Gastric aspirates: ≥100% pre-feed volume (2 hours feeding volume if continuous feeding). Lower limit=2 ml/kg.
5. Stool frequency: number of stools per day.

6. Time to regain birth weight: first day of three success days when the infant weight is greater than birth weight. Indicate 0 days, if the infant never has a weight below birth weight.
7. Weight, height and head circumference at 7, 14, 21 and 28 days, the end of intervention (≤ 34+0), gestational week 36+0, at discharge from neonatal ward (or at gestational week 44+0, whatever comes first) and at 2 years of age (corrected) and 5.5 years of age (uncorrected). Standard deviation score (based on Niklasson [33]) will be used in the calculations.

#### 5.3.2.2 Clinical variables for morbidity

**-Neonatal period:**

1. Mortality

2. Necrotising enterocolitis (NEC): Bell´s stage II-III [34]. Stage II requires clinical signs of NEC with abdominal symptoms and radiological findings such as intramural and/or portal gas. Stage III requires the same findings as stage II in addition to heavily affected clinical condition (chock, need of respirator, mortality) and/or surgery. If surgery confirms NEC, there is no need of positive radiological findings for diagnosis. Radiological assessment will be made blinded by an independent radiologist. The final decision will be confirmed by a blinded consensus panel review consisting of the investigators. The age (in days) of onset will be noted. If the NEC diagnosis is confirmed, this diagnosis will replace any eventual sepsis diagnosis during the duration of the NEC episode.

3. The composite of NEC and culture-proven sepsis: An infant should have had any of these two diagnoses to fulfil the criterion. Please see the definition of NEC above and sepsis below.

4. Spontaneous intestinal perforation (SIP: intestinal perforation without signs of intramural and/or portal gas and no signs of inflammation at surgery.

   *If there is an intestinal perforation it is important that the investigator rules out whether it is NEC or SIP, since the underlying mechanism probably differs.*

   *Although NEC criteria is not fulfilled, e.g. because the independent radiologist do not agree that the radiology findings are enough for the diagnosis, the clinical findings and laboratory results might fulfil the criteria for sepsis (please see below).*

5. Abdominal surgery

6. Culture-proven sepsis: Positive blood- and/or urine- and/or CSF culture, clinical deterioration and laboratory inflammatory response*

   *Laboratory inflammatory response (<u>one</u> of the following is needed) [35]:
   1. LPK ≤ 5 or ≥ 20 ($x10^9$ cells/L)
   2. TPK ≤ 100 ($x10^9$ cells/L)
   3. CRP ≥ 15 mg/L

   *Culture proven sepsis should further be classified into early (<72 hours postpartum) or late (≥72 hours postpartum) onset. Also, the age (in days) at onset will be noted.*

The infant shall not get a sepsis diagnosis if it has got a NEC diagnosis during the same period

Suspected sepsis, not culture-proven: clinical deterioration and laboratory inflammatory response* but negative blood culture. Age at onset will be noted.

7. Pneumonia: Pathological X-ray confirmed by an independent radiologist, need of increased respiratory support/oxygen and laboratory inflammatory response* Age at onset will be noted.

8. Bronchopulmonary dysplasia (BPD): Need of extra oxygen, high flow nasal cannula, CPAP or ventilator at gestational week 36+0 [36]
9. Retinopathy of the prematurity (ROP): diagnosed by an independent ophthalmologist according to international classification. [37] Classified into stage I-V. The diagnosis is set after gestational week 42+0.
10. Intraventricular haemorrhage (IVH): classified into grade I-IV according to Papile [38]. Assessed by independent radiologist if the onset was after the intervention started. Age at onset will be noted.
11. Periventricular leukomalacia (PVL). Criteria according to de Vries [39] assessed by an independent radiologist.
12. Number of days with intensive care: need of respirator or CPAP until discharge (not later than gestational week 44+0).
13. Length of stay at the hospital: gestational week and day at discharge (not later than gestational week 44+0).
14. Length of need of feeding tube: gestational week and day when the infant does not need it anymore (not later than gestational week 44+0).

**2-year follow up**

1. Weight, height and head circumference.
2. Need of feeding tube after discharge from the hospital at the neonatal period (not later than gestational week 44+0)
3. Extra nutritional support after discharge from the hospital at the neonatal period (not later than gestational week 44+0)
4. Neurocognitive development: Bayleys III [40]
5. Cerebral palsy
6. Epilepsy
7. Squint and/or impaired vision
8. Impaired hearing
9. Need of extra oxygen and/or ventilatory support after discharge from the hospital at the neonatal period (not later than gestational week 44)
10. Wheeze and/or asthma
11. Severe infections after discharge from the neonatal unit (not later than gestational week 44).
12. Mortality: including cause of death.
13. Level of education of the parents
14. Family status
15. Day-care

**5.5 year follow up**

1. Weight, height and head circumference.
2. Neurocognitive development: Wechsler Preschool and Primary Scale of Intelligence IV (WPPSI-IV $^{TM}$) and Movement ABC-2
3. Cerebral palsy

4. Epilepsy
5. Squint and/or impaired vision
6. Impaired hearing
7. Wheeze and/or asthma
8. Mortality: including cause of death.

   Data from the 2 and 5.5 follow up will be obtained from the Swedish neonatal quality register (www. snq.se

### 5.3.2.3 Laboratory variables

Separate protocol for each laboratory analysis will be created. Time points of collection of samples are displayed in the Flow chart (paragraph 2).

1. Microbiology analyses: Bacterial DNA will be extracted from stool and breast milk and samples analysed with high-throughput next generation sequencing methods. Shotgun metagenomics will be performed to sequence the whole bacterial genome in order to analyse bacterial functional genes. Specific focus will be on genes for degradation of oligosaccharides, formation of short chain fatty acids (SFCA), vitamin production, intestinal barrier function and virulence factors. Because of the prospective design the analyses could be performed in samples collected before the onset of an outcome parameter- thereby providing evidence related to a cause-and-effect relationship. Gut bacteria metabolites in faeces such as SCFA will be assessed with gas chromatography and be related to the microbiology analyses and the clinical outcome.

2. Immunological analyses in blood samples: Blood samples are collected in EDTA tubes for plasma and separate pre-prepared tubes for masscytometry of blood cells (CyTOF). The plasma and cells will be stored at -70°C. The purpose of the immunological analyses is not only to compare the active and control group to assess the effect on the immune system of human milk-based and bovine-based nutrient fortifier, respectively, but also to map the immune system in this extreme group of patients and to identify immunological markers associated with specific diseases such as NEC and BPD.

    1. T helper subsets (TH1, Th2, TH17, Treg), T cells subsets associated with the intestinal mucosa (γδ-T cells, MAIT cells) and neutrophils will be assessed using masscytometry.
    2. Levels of Anti- and proinflammatory cytokines (e.g. IL-10. IL-6, TNF-α) and chemokines (e.g. CXCL11, CCL18) as well as immune modulatory enzymes such as Indoleamine 2,3-dioxygenase (IDO) are high enough in plasma to be measured even in these small infants. To use the plasma as efficiently as possible, it will be analysed using multiplex assays.

3. Growth factors and neurotransmitters in blood samples: The levels of growth factors such as IGF-1 and the associated IGFBP-3 will be analysed. Besides being important for growth, these factors are also important in the pathogenesis of several of the complications (ROP, BPD) under study in this project. Neurotransmitters, which affect gut motility and neurological development, have been associated with the gut microbiota and will also be analysed. Markers for CNS damage such as Neurofilament light protein will be analysed in plasma.

4. Lipidomic analyses will be performed in plasma samples and erythrocyte membranes to assess the effects of the different feeding regimens on lipid profiles

5. Metabolomic analyses will be performed in urine samples. Urine is collected using cotton wool (first urine after 8 am), centrifuged and divided to 2-3 aliquots of about 1 mL of urine and stored at -70°C. Low molecular weight metabolites (metabolome), intermediate or products of metabolism in human cells and gut bacteria, will be analysed with different techniques such as proton nuclear magnetic resonance spectroscopy (NMR), liquid chromatography (LC) and mass spectroscopy couple to gas chromatography (GC-MC). These methods allow us to prospectively identify potential biomarkers of malnutrition in ELGA infants and potential metabolic mechanism underlying the effect of human milk-based nutrient fortifiers.

6. Proteomic analyses in breast milk samples after fortification from the mothers but also from the donor milk that the infant receives at the day of sample collection (at 7, 14, 21 and 28 days and w33 of life). Also human milk oligosaccharides will be measured in the breast milk samples. The protein profile in breast milk samples and in $H^2$ MF and the cow milk protein-based fortifier will be related to feeding tolerance, NEC and other complications during the neonatal period in order to identify specific proteins or patterns of proteins important for the outcomes

7. Vitamins and micronutrients will be analysed in a sample of infants with standard methods to evaluate if the different fortification may lead to different levels in blood.

#### 5.3.2.4 Health economics

The intervention is costly but may prevent important complications such as NEC, sepsis and food intolerance and may thereby reduce the overall cost of neonatal care. We will use level of care to calculate health care costs.

1. The number of days at each level of care (as defined in Appendix 11) will be recorded until discharge from the hospital (not longer than gestational week 44+0).
2. The cost will be calculated by multiplying the number of days at each level of care by the average cost.

### 5.3.3 Associated predictor variables (covariates)

1. Gender
2. Caesarean section
3. Multiple pregnancies.
4. Birth weight and height.
5. Small for gestational age (SGA): Birth weight <2 SD.
6. Maternal smoking during pregnancy.
7. Preeclampsia: diagnosis by the responsible obstetrician.
8. Chorioamnionitis: diagnosis by the responsible obstetrician.
9. Preterm premature rupture of the membranes (PPROM). Rupture ≥ 1 hour before contractions started.
10. Antenatal antibiotics: pertain the period of the mother´s actual attendance at the hospital.
11. Antenatal corticosteroids: the mother should have received at least 12 mg Betametasone. The corticosteroid prophylaxis is considered completed if the mother has received 2 doses at least 24 hours before delivery.
12. Born at level 1-2 NICUs.
13. Apgar score.
14. Surfactant-administration.

15. Intubation.
16. Infant respiratory distress syndrome (IRDS): Verified by X-ray
17. Respirator duration.
18. Patent ductus arteriosus (PDA): requiring medical or surgical treatment.
19. Antibiotics: name, treatment period and number of days.
20. Probiotics: name, treatment period and number of days
21. Opioids: name, treatment period and number of days
22. Gastric acid inhibitors: name, treatment period and number of days
23. Day of life when the supplementation of the study product was started
24. The amount of enteral feeds per day that the infants received when the supplementation of the study product was started
25. Number of days the infants has not received the study product
26. Number of days with intravenous line
27. Number of days with insulin treatment (and treatment period)
28. Number of days and doses of postnatal corticosteroids
29. Number of days with inotropic drugs
30. Feeding regime: continuous or bolus.
31. Amount of nutrient protein fortifier per day
32. Amount of fat supplement per day
33. Total amount of protein, fat, carbohydrate, energy and micronutrient per day.
34. The relative amount of donor breast milk per day
35. Amount of extra enteral lipid
36. Breastfeeding: exclusive, partial.

# 6 Study design

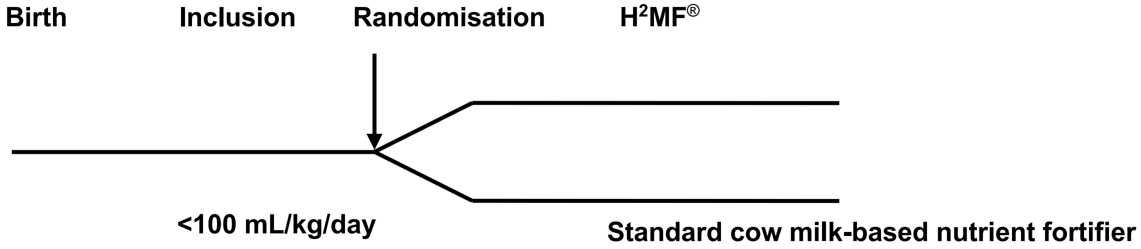


## *6.1 Design*

This is a randomised controlled multi-centre trial comparing diet supplementation with human breast milk-based nutrient fortifier H$^2$MF® and standard bovine protein-based nutrient fortifier in extremely preterm infants (born before gestational week 28+0) exclusively fed with human breast milk (own mother´s milk and/or donor milk). The infants will be randomised to receive either the human breast-milk based H$^2$MF® or the standard bovine protein-based nutrient fortifier before oral feeds have reached 100 ml/kg/day. If fortification with extra enteral lipids is needed during the intervention period, the infants receiving H$^2$MF® will be supplemented with the human milk-based Prolact CR®, while the infants receiving standard bovine protein-based fortification will be supplemented with the standard lipid products used at the unit. The study subjects will be enrolled at level III NICUs. Only infants with a home clinic with the logistics to maintain the intervention until gestational week 34+0 will be included.

The intervention will continue until gestational week 34+0, but terminates earlier if the infant is transferred to a clinic without the logistics needed for the intervention, or if the clinic terminates the supplementation with donor breast milk earlier than gestational week 34+0 and the infant is not fed completely with mother´s own milk. After the intervention has ended there should be a transition period when the fortification of the breast milk during a 5-day period gradually goes from 100% of $H^2$ MF to 100% of standard bovine protein-based fortifier, if protein fortification still is needed. The infant must not be fed with formula during the intervention period. Thus, the interventions terminate when the infant receives the first dose of formula. A specific transition protocol is required in order to gradually wean the infant off the exclusive human milk diet. Please see Appendix 10.

Randomisation will be stratified by inclusion centre, gestational week (22+0-24+6 and 25+0-27+6), gender and parity. The allocation will be concealed before inclusion, but after randomisation the study is not blinded

The enrolled infants are characterized with clinical data including growth, feeding intolerance, use of enteral and parenteral nutrition, treatment, antibiotics and complications collected daily in a study specific case report form from birth until discharge from the hospital (not longer than gestational week 44+0). A follow up focusing on neurological development, growth and feeding problems will be performed at 2 and 5.5 years of age (corrected). Stool, urine, blood and breast milk samples are also collected for microbiology, metabolomic, proteomic and immunology analysis in order to study underlying mechanisms. Biopsies from the intestine will be collected if acute surgery is performed. Health economic analyses will be done to evaluate the costs and benefits of introducing a human milk-based fortifier into Nordic countries.

## 6.2 Selection of study design

There is no trial in extremely preterm infants comparing a human milk-based breast milk fortifier with bovine protein-based breast milk fortifier in which both study groups receive exclusive human breast milk. Such a trial is needed to provide evidence that a human milk-based fortifier is superior to a bovine-based one in extremely preterm infants also in centres with 100% coverage of breast milk donor banks. Moreover, due to the introduction of active intervention for infants also born before gestational week 25 during the last decade, a trial run in Nordic centres will have a predominance of infants born in gestational week 23-25, a patient population that could be expected to gain the most of a diet free from bovine protein. The trial will be prospective randomised controlled to achieve the highest level of evidence. However, it will not be blinded. It would not be possible to prescribe the fortifier and prepare the breast milk in a blinded fashion, since the fortifiers are not exactly equal in nutrient content and also look different. Instead the assessment of several of the outcomes will be made blinded, such as the radiological assessment of NEC. The trial will also have multi-centre design in order to have an inclusion rate high enough but also to evaluate feasibility and generalisation in different NICUs. Since it is often difficult to distinguish between the diagnoses of NEC and sepsis, and the clinical consequences of NEC and sepsis are equal, our primary endpoint of the intervention is the composite variable of NEC, sepsis and mortality.

## 6.3 Selection of treatment

Preterm infants receiving human milk have better feeding tolerance and lower incidence of NEC than those fed cow milk based preterm formula [9]. Thanks to a high coverage of donor milk banks, extremely preterm infants are exclusively fed with human breast milk in the Nordic countries. Despite this, the incidence of NEC in extremely preterm infants is still as high as 12% according to the Swedish National Quality Register (www.snq.se). In extremely preterm infants, breast milk has to be supplemented with a multi-nutrient fortifier in order to provide extra protein and micronutrients needed to achieve adequate growth and body composition. However, the fortifiers used in the Nordic Countries are bovine protein-based. Thus, even when the base diet is 100% human milk, extremely preterm infants receive substantial intakes of cow's milk protein – indeed often a higher intake than from the human milk diet since breast milk contains such a low protein level for the needs of extremely

preterm infants.

H$^2$MF® is one of a range of human milk-based milk fortifier with different protein contents (but with identical contents of micronutrients) designed for preterm infants by Prolacta Bioscience, CA, USA. H$^2$MF® was selected for this trial since the protein level (at 2.7 g/100ml) was closest to that commonly provided using cow's milk-based fortifiers available in Nordic countries. Previous trials evaluating this human milk-based fortifier in extremely preterm infants receiving exclusive human milk diet, suggest that exclusive human milk diet, devoid of cow milk-containing products, is associated with lower mortality and morbidity in these infants without compromising growth [3,10,11]. Thus, such an exclusive human milk diet has been reported to reduce the incidence of NEC, sepsis, BPD and ROP. No side effects have been reported. However, there is no trial in extremely preterm infants comparing a human milk-based fortifier with bovine protein-based fortifier in which both study groups receive exclusive human milk base diet. Such a trial is needed to provide evidence that a human milk-based fortifier is superior to bovine-based one in extremely preterm infants also in centres with 100% coverage of breast milk donor banks. A significant positive result of this trial could easily be implemented in the NICUs, given the existence of the product and the extensive prior experience of its usage in well over 200 level 3 neonatal units, notably in the USA. The manufacturer recommends the use of the human milk-based Prolact CR® as the fat supplement to infants receiving H$^2$MF®. Therefore, if an infant in the H$^2$MF® group needs extra enteral fat during the intervention period, it will be supplemented with Prolact CR®. An infant receiving standard bovine protein-based fortification will be supplemented with the standard lipid products used at the unit.

# 7. Study population

## *7.1 Sample size*

The incidence of the primary outcome, the composite of NEC, culture-proven sepsis and mortality, was 47.4% (1153/2428) in extremely preterm infants in 2007-2014 according to the Swedish Neonatal Quality Register (www.snq.se). Only infants surviving more than 3 days were included in this estimation since the inclusion will take place after 3 days of life. The NEC incidence (incl. NEC cases that also had a late onset sepsis episode) was 12.6% (305/2428). The sepsis incidence (without NEC) was 29.7% (720/2428), and the mortality incidence (without sepsis and NEC) was 5.3% (128/2428). The reduction of sepsis in the exclusive human milk fed group using Prolacta human milk-based products in a previous trial was 37.2% [3]. With a similar effect on sepsis there would be a reduction to 20.9% (507/2428) in the H$^2$MF® group in our proposed trial (incl. 42 sepsis cases from the NEC with sepsis group above because as we estimate a 60% reduction of NEC, please see below). Since there is not any previous trial with 100% coverage of breast milk it is difficult to estimate the effect on NEC from previous trials. However, a conservative estimation would be a 60% reduction of the NEC incidence to 5.4 % (131/2428), as compared to US studies cited above where the fall in NEC was around 75%. Since many deaths may reflect undiagnosed NEC and sepsis episodes, mortality is estimated to be reduced by 25% to 4.0% (96/2428). Thus, the incidence of the composite outcome is estimated to be reduced to 28.0% (680/2428). With at least 101 infants in each group a reduction from 47% in the control to 28% in the active group would be detected at a 5% level of significance and with 80% power using a large sample Gaussian approximation to the comparison of two binomial proportions (using a pooled estimate of variance). Thus, the aim would be to enrol a total of 222 extremely premature infants in the trial to allow for an approximate 10% dropout rate.

However, because there are uncertainties in the effect size presupposed here, the study will be conditioned power in the following sense. An evaluation of the overall rate of the primary clinical endpoint of NEC/sepsis/death will be made prior to the formal analysis of that aspect of the study data in order to determine whether the trial sample size should be re-evaluated and increased in order to continue study enrolment. This evaluation will be based on the methodology suggested by Gould *et al.* [41]. Let $\sigma$ be the pooled rate of this study endpoint in the two arms, i.e.

$\sigma=(p_{control}+p_{human\ fortifier\ group})/2$, where p is the incidence of the primary study endpoint in the respective groups. Let $\sigma^*$ be the same value, but based on some assumed rates for these rates from the beginning of the trial. Then the following sample size multiplier is determined:

$f = [\sigma(1-\sigma)]/\sigma^*(1-\sigma^*)$

If f is greater than 1, then the new sample size, $n_1$, is determined as f x n, where n is the original sample size (per group) of 101. If f is less than one, then the study may be analysed at that point for the primary clinical endpoints NEC/sepsis/death. Note that an independent statistician not associated with the study conduct will perform this conditional analysis. An independent statistician will make a sample size re-estimation when 150 infants have been included. Thus, the definitive sample size might be increased (never decreased) based on this interim analysis.

Theoretically, if the effect is very low, the calculation according to Gould will suggest a very high number of infants needed. Thus, there is a need of a clinically relevant upper limit of the inclusion. With a conservative estimation of a 50% NEC reduction in the $H^2MF^®$ group, a reduction of the composite outcome from 47% in the control and 31% in the active would be detected with at a 5% level and 80% power with 145 infants in each group. Based on that estimation, the upper limit of included infants would be 322 to allow for an approximate 10% dropout rate.

An estimation how many infants that would be needed for the composite of culture-proven sepsis and NEC has also been made, since this secondary outcome could be of special interest, as it is not affected by mortality rates. The incidence of this outcome was 42.2% (1045/2428) and could be estimated to be reduced to 26.2% (638 /2428) based on the background data described above. With at least 142 infants in each group, a reduction from 42% in the control to 26% in the active group would be detected at a 5% level of significance and with 80% power. Thus, with a dropout rate of 10% in total 312 infants would be needed for this outcome. A similar evaluation according to Gould *et al.* as described above will also be made for this outcome after 150 infants have completed the study in order to determine whether the trial sample size should be re-evaluated and increased.

## 7.2 Inclusion criteria

- Gestational age at birth 22+0-27+6: based on prenatal ultrasonography.
- Enteral feeds < 100 mL/kg/day at the day of randomisation.
- Written informed consent from the legal guardians of the infant.
- The home clinic of the infant has the logistics of maintaining the intervention until gestational week 34+0

## 7.3 Exclusion criteria

- Lethal or complicated malformation known at the time of inclusion
- Chromosomal anomalies known at the time of inclusion
- No realistic hope for survival at the time of inclusion
- Gastrointestinal malformation known at the time of inclusion
- Abdominal surgery before the time of inclusion
- Participation in another intervention trial aiming at having an effect on growth, nutrition, feeding intolerance or severe complications such as NEC and sepsis
- Infants having nutrient fortifier or formula prior to randomisation

## *7.4 Withdrawal criteria*

Subjects may be discontinued from the study for the following reasons:

- If it is in the best interest of the subject, based on the responsible physician's discretion.
- The parents/legal guardian, cancel the informed consent agreement.

The reasons for exclusion of a subject should be stated in the Case Report Form (CRF). Discontinued subjects should always be included in the safety and tolerance analysis.

Parents have the right to discontinue participation in the study at any time. Although the infant for any reason - physician's or parents' decision - stops receiving $H^2MF^{®}$, he or she will still continue to be included in the study for clinical data collection, unless the parents oppose that. Thus, the infant will be included in the intention-to treat analyses as long as CRF protocol is completed to gestational week 36+0. That also applies to situations when the intervention ends because the infant has received formula or bovine-based nutrient fortifier. Note that parents do not have to give any reasons why to cancel the study. If parents wish to avoid sampling, they will be asked if the infant can remain in the study even if the sampling is omitted.

If the stated reason for discontinuation is an adverse event, the principal investigator is to actively participate in all actions until the problem is resolved or the situation has become stable.

The infant will not be excluded from the study if $H^2MF^{®}$ has not been supplemented due to poor study compliance. Instead, a per protocol analysis will be made based on the length of the supplementation (please see statistics in section 20). The parents will be asked if their infant is included in another trial, both at inclusion and the end of the trial. The infant can be included in another trial as long as the other trial does not influence the infants' nutrition, growth, feeding tolerance and risk of NEC.

The attending physician or investigator has the right to withdraw the infant from the trial at any time on the assessment made that continued participation would be a serious health risk.

A transfer to a clinic that does not participate in the study is not a reason for withdrawal. The situation is handled according to 9.1.12.

## *7.5 Study termination*

Please see the section 7.1 regarding study population. By ethical reasons, the study can be terminated prematurely based on a decision of the sponsor and the DSMB, if the primary outcome is significantly lower (with a significance level <0.001) in the $H^2MF^{®}$ than in the standard fortification group in the interim analysis made after 150 infants have completed the neonatal period. The study can also terminate prematurely if any severe complication is significantly more common in the $H^2MF^{®}$ than in the standard fortification group in any interim analysis included in the safety analyses in the trial, please see section 13.3 and 13.4.

# 8 Study period

In total 222 extremely preterm infants are planned to be included. 270 extremely preterm infants are born in the catchment area of Gothenburg, Linköping, Stockholm, Uppsala and Umeå every year. Since not all available infants could be expected to be enrolled, the inclusion time is supposed to be approximately two-three years.

# 9 Methods

## *9.1 Practical arrangements*

### 9.1.1 Inclusion and randomisation

The study subject will only be enrolled at level III NICUs. The infants should be randomised 1:1 to receive either the human breast-milk based $H^2MF^{®}$ or the standard bovine protein-based nutrient fortifier before oral feeds have reached 100 ml/kg/day. The written informed consent could preferably be obtained from the parents well before the oral feeds have reached 70 ml/kg/day in infants that are expected to fulfil the inclusion criteria, since they should have time enough to give a well-thought-out consent. Thus, in practice, the informed consent can be obtained already at the second and third day of life but not after the oral feeds have passed 100 ml/kg/day.

Randomisation, however, can only be done when all the inclusion criteria are fulfilled, and there are no exclusion criteria.

Procedures:

- Control that the infant fulfils all the inclusion criteria, please see section 7.2
- Control that the infant does not have any exclusion criteria, please see section 7.3
- Clear oral information to the parents, use an interpreter if needed, and give them the written information (Appendix 6).
- Clear information to the parents that the participation is optional, and that they can withdraw their infant´s participation at any time without explaining why. Also inform about the basics in EU´s General Data Protection regulation (GDPR).
- All eligible infants should be added in the Screening log anonymously. Thus, all infants at an inclusion site that were born before gestational week 28+0 and survived during the first 3 days should be included in the Screening log. That means that also infants that by accident reached enteral feeds >100 mL/kg/day before there was an opportunity to include them should be included in the Screening log. Also, eligible infants belonging to the region but born and treated outside the inclusion site should be included in the Screening log.
- The informed consent form should be signed by legal guardians (appendix 7). If there is only one legal guardian, an informed consent from this person is sufficient. The attending physician shall note this in the medical record of the infant.
- The information can be given via telephone, if one of the parents cannot attend at the information at the hospital. This parent has to sign the informed consent form and send to the hospital before inclusion.
- The informed consent form should also be signed by a physician. It does not have to be done at the same date as the parents but it must be before the randomisation.
- Randomisation is made according to section 12.5. The legal guardians are informed what study group their infant belongs to. The allocation and the study identification number (ID) should also be indicated in the Identification list and the CRF immediately.
- All infants included in the study should be added in the Identification list, which include the infants' personal number ("personnummer") and the patient ID. Inclusion sites have separate series of patient identification number: Umeå starts with 01001, Uppsala with 02001, Linköping with 03001, Göteborg

with 04001, Örebro (Uppsala´s catchment area) with 5001, Solna (Stockholm´s catchment area) with 6001 and Huddinge (Stockholm´s catchment area) with 7001. New cases are added consecutively at each site. Study group allocation is also added in the identification list. This list and the Screening log should be kept safe in the Investigator file at each study centre.

- The intervention is started as described in section 9.1.4
- A CRF (Appendix 8) is prepared and background data is filled in. The day the child was born is considered as day 0. Day 1 starts at moon (24.00/0.00).
- The study nurse at the study site should be informed.
- The tasks of the study nurse:
    - Inclusion and exclusion criteria are controlled.
    - Check that the Screening log and the Identification list are completed.
    - Note in the medical records:
      ”Patienten deltar i den kliniska prövningen N-Forte. Barnets PatientID/StudieID är:_________.
      Studiegrupp:__________ ( skriv in H2MF eller standardberikning)
      H2MF är en humanbaserad bröstmjölksberikning och effekten av denna är det som testas i studien.
      Interventionen pågår från inklusionsdag__________ till gestationsvecka 34+0.
      Ansvarig prövare på denna studieort är:_______________________.
      Ansvarig prövare för hela studien är Thomas Abrahamsson, Region Östergötland.
      Föräldrar har lämnat informerat samtycke, datum___________.”
    - Write the Patient-ID in medical files of the infant; both in the medical records and the Cardex (if there is one at the study site)
    - Label all study material of the included infant with the Study ID.
    - The informed consent is stored in investigator file.
    - Give a copy of the signed informed consent form to the parents/legal guardians.
    - Prepare a study file for the infants.

### 9.1.2 Twins

Twins and larger multiple births will be allowed into the study. Randomisation will be stratified by parity to avoid imbalanced randomisation of multiple births. Twins will be randomised together. Thus, they will get the patient identification number and also the same nutritional protocol. Twin number one will get the suffix A and number 2 the suffix B (e.g. 101 A and 101 B). There will also be stratification for gender. Because of limitations of randomisation/stratification procedure, a twin pair will be considered having the same gender when the randomisation is made. Thus, enter the gender of the first-born twin in the program (please see paragraph 12.5)

### 9.1.3 Investigator file

An investigator file will be kept locked at each study site. It should include the study protocol (including all appendices), parental information, informed consent form, CRF, copy of the ethical approval, screening log and identification list.

### 9.1.4 Start of the intervention

The intervention starts before the enteral feeds have reached 100 mL/kg/day, the parents have given their written informed consent (please see 9.1.1) and randomisation is completed (please see 12.5). Prescription and administration of the study product is described in 9.1.5.

### 9.1.5 Prescription, preparation and administration of the study product

A specific protocol of preparing H$^2$MF® is described in Appendix 17. The standard bovine protein-based milk fortifier used at the specific site will be prepared according the recommendation of manufacturer. Each centre will have a co-ordinator associated to the milk kitchen where the study product is prepared.
It is essential that the clinician and/or the dietician that prescribe the enteral feeding knows in which study group the individual patient belongs to.

1. The attending physician prescribes the enteral nutrition every day during the NICU stay, including the source of breast milk (own mother's or donor milk), total volume and the level of fortification (normal, half normal or no fortification), based on which intervention group the infant is allocated to. The study is not blinded for the attending physician.

    a. The level of fortification is prescribed stepwise according local guidelines in order to achieve appropriate protein intake~~,~~ see Appendix 9.
    b. The daily level of fortification for each infant is primarily based on protein intake and the aim is 4.0-4.5 g/kg/day, even though a lower intake is acceptable when the infant is approaching term age and is growing adequately.
    c. Macronutrient analyses of own mother's milk are performed weekly using an infrared breast milk analyser. Breast milk analyses of donor breast milk are performed once for each batch.
    d. The daily prescription is based on the analysed protein content in the breast milk which the infant receives. Charts for fortification levels for each study product and for different ranges of breast milk protein content are found in Appendix 9.
    e. Daily prescriptions are individualized, taking clinical condition, fluid balance and parenteral nutrition into consideration
    f. Daily prescriptions should be done in order to ensure that intakes of all nutrients are within recommended ranges. The Nutrium software (Nutrium AB, Umeå, Sweden) which is provided to all study sites has included the study products in its algorithms. Support from the level III sites will be given to physicians and dietitians if needed for the nutritional calculations.
    g. Additional vitamins (Multivitamin Unimedic, typical dose 7 drops/kg [0.25 mL/kg] for infants < 2 kg) always need to be prescribed to infants who receive the H$^2$MF product.
    h. When less than full dose fortification is given, additional micronutrient supplements (especially calcium and phosphorous supplements) are usually needed.
    i. Fat supplements can be considered if energy intake is low and growth is suboptimal. The infants receiving H$^2$MF® will be supplemented with the human milk-based Prolact CR®, while the infants receiving standard bovine protein-based fortification will be supplemented with the standard lipid products used at the unit.

2. The preparation of fortified milk is performed in the NICU milk kitchen by trained staff according to the manufacturer's instructions, the prescribed dose and the mixing table in Appendix 9 and the investigator file. The milk is prepared in advance for each meal during the next 24-hour period. Fortified milk bottles are marked with patient name and/or ID and date and kept refrigerated until use. The staff in the milk kitchen are not blinded to the intervention.

3. The milk is fed to each infant by the attending nurse according to local guidelines.

.

### 9.1.6 Case report form (CRF) and Nutrium

The CRF is established at randomisation. The study nurse fills in all the background factors from pregnancy and birth in collaboration with the investigators of the centre. The CRF will cover the period in the neonatal ward from birth, i.e. also before inclusion, until discharge from the hospital/neonatal home care (but not longer than GW 44+0). The day the child was born is considered as day 0. Day 1 starts at moon (0.00 am).. Regarding the transfer to another clinic, please see 9.1.11. Upon discharge home the CRF should be completed including evaluation of clinical diagnoses. The CRF should be controlled and signed by any of the investigator at the actual site. The original CRF shall be submitted to the sponsor/PI after been monitored by the study monitor. One of the copies should be submitted to the data entrance company. One copy of the CRF should always be stored at the home clinic.

All data on nutrition and other fluids will be entered daily in the Nutrium software (Nutrium AB, Umeå, Sweden) which is provided to all study sites. Support will be given to physicians and dietitians if needed for the nutritional for this entrance of data. All nutritional data will be entered from birth to gestational week 34+0, when the intervention ends.
The data of infants included in the N-forte trial will be transferred to the database after the study has been completed.

### 9.1.7 Samples at 7 ± 2 days of age

Note adverse events and possible antibiotic treatment since the previous registration in the CRF. The actual weight, length and head circumference at 7 days of age is noted in the CRF.
Stool, urine, blood and breast milk samples are collected by the nurse according to the sampling instructions (Appendices 12-15). Breast milk samples are only collected if the mother has an excess of it. It is the breast milk that infants receive at day 7 that should be collected. Blood samples are only collected when routine blood sampling is made. Note in the CRF that the samples have been collected.

Label the tubes CLEARLY with the study specific adhesive label, study identification number type of samples (stool, urine), "Day 7" and the date of the sampling. WITHOUT IDENTIFICATION NUMBER AND DATE THE SAMPLE IS USELESS!

### 9.1.8 Samples at 14 ± 2 days of age

Note adverse events and possible antibiotic treatment since the previous registration in the CRF. Actual weight, length and head circumference at 14 days of age is noted in the CRF.
Stool, urine, blood and breast milk samples are collected by the nurse according to the sampling instructions (Appendices 12-15). Breast milk samples are only collected if the mother has an excess of it. It is the breast milk that infants receive at day 14 that should be collected. Blood samples are only collected when routine blood sampling is made. Note in the CRF that the samples have been collected.

Label the tubes CLEARLY with the study specific adhesive label, study identification number, type of samples (blood, stool, breast milk, urine), "Day 14" and the date of the sampling. WITHOUT IDENTIFICATION NUMBER AND DATE THE SAMPLE IS USELESS!

### 9.1.9 Samples at 21 ± 2 days of age

Note adverse events and possible antibiotic treatment since the previous registration in the CRF. Actual weight, length and head circumference at 21 days of age is noted in the CRF.

Stool, urine and breast milk samples are collected by the nurse according to the sampling instructions (Appendices 13-15). Breast milk samples are only collected if the mother has an excess of it. It is the breast milk that infants receive at day 21 that should be collected. Note in the CRF that the samples have been collected.

Label the tubes CLEARLY with the study specific adhesive label, patient identification number, type of samples (stool, urine), "Day 21" and the date of the sampling. WITHOUT IDENTIFICATION NUMBER AND DATE THE SAMPLE IS USELESS!

### 9.1.10 Samples at 28 ± 2 days of age

Please, see sample collection at 14 ± 2 days of age (9.1.8).

Label the tubes CLEARLY with the study specific adhesive label, patient identification number, type of samples (stool, urine), "Day 28" and the date of the sampling. WITHOUT IDENTIFICATION NUMBER AND DATE THE SAMPLE IS USELESS!

### 9.1.11 The end of the study intervention, gw 34+0

The intervention ends at gestational week 34+0, but can end earlier (please see 9.1.12). Note in the CRF the actual date the intervention ends. Actual weight, length and head circumference is noted in the CRF. **Samples shall be collected before intervention ends!** Please, see 9.1.13

Infants that has completed the study to gestational week 34+0:

1. The $H^2$ MF group:
   After the intervention has ended there should be a transition period when the fortification of the breast milk during a 5-day period gradually goes from 100% of $H^2$ MF to 100% of standard bovine protein-based fortifier, if protein fortification still is needed (please see Appendix 10). Still it is the staff in the milk kitchen that prepares the breast milk by using the transition protocol.
   A transition protocol must also be applied if formula is going to be introduced directly after the intervention period ((please see Appendix 10). If the infant has received Prolact CR as fat supplement it should end after the transition period is over. The standard lipid supplement will then be used if the if the child still need extra lipids.
2. The standard bovine protein-based fortifier group:
   The protein fortification continues according the routine of the clinic.

Infants that end the study intervention before gestational week 34+0

The most common reason for early discontinuation is that the infants are going to be transferred to a study site without the logistics for maintaining the intervention.

1. The $H^2$ MF group:
A transition period is needed, when the fortification of the breast milk during a 5-day period gradually goes from 100% of $H^2$ MF to 100% of standard bovine protein-based fortifier, if protein fortification still is needed (please see Appendix 10). The staff in the milk kitchen at the new site will prepare the breast milk by using the transition protocol. It is crucial that the transition protocol (Appendix 10), the product description (Appendix 17) and $H^2$ MF much enough for the transition period, follow the infant to the new site. The investigator should ensure that the attending physician at the new site get information about the study at the day of transferal.

2. The standard bovine protein-based fortifier group:
The protein fortification continues according the routine of the clinic.

### 9.1.12 Transfer to another neonatal unit

Take copy of the CRF. Keep the original in the study folder of the infant and send a copy to the receiving clinic. Basically, only infants with a home clinic that will be able to continue with the study product will be enrolled in the trial. However, if the receiving unit does not have the logistics to continue with the products until gestational week 34+0, a transition protocol shall be applied as described in section 9.1.11. The attending nurse and physician report that the infant is included in the study, that the transition protocol has to be followed and how to fill in the CRF.

Thus, the aim is to continue with the intervention at the new clinic until gestational week 34+0. The responsible physician and nurse should report to the receiving unit in which study group the infants belong to. Also, the investigator and the study nurse and/or the staff at the milk kitchen shall confirm with the receiving unit that they have got the information. It might be a time gap of 1-3 days before this information can be forwarded (at weekends etc.). While waiting for this information, the infant must not get any bovine protein-based fortifier or formula. If the staff at the new clinic do not want to wait with the fortification, they can use $H^2$ MF. However, when the staffs have got the information about the study group, the infant should start with the right study product. The dietician at the original clinic will be a resource helping smaller neonatal units calculating the degree of fortification. In most cases the fortification does not have to change during the short time period that is left until the infant has reached gestational week 34+0.

The study nurse will contact the new clinic as soon as possible to help them with the CRF, sampling etc. In neonatal units that belong to the catchment area of Gothenburg, Linköping, Stockholm, Uppsala and Umeå, there will be one principal investigator and nurse that will co-ordinate procedures at their site. The principal investigator will be responsible for completing and signing the CRF when the neonatal follow up period has ended at discharge from the hospital. The fulfilled part of the original CRF shall be submitted to the sponsor/PI after been monitored by the study monitor. One of the copies of this part should be submitted to the data entrance company. One of these copies should follow the infant to the next study site. One copy of the CRF should always be stored at the home clinic. The non-used (empty) part of the original CRF should follow the infant to the next study site

### 9.1.13 Sample at gestational week 33+0-33+6

Please, see sample collection at 14 ± 2 days of age (9.1.8). The sample shall be collected before the interventions ends gw 34+0. Topical anaesthesia can be used before the blood sample is collected and a larger volume can be taken, 2.5 mL

Label the tubes CLEARLY with the study specific adhesive label, patient identification number, type of samples (stool, urine), "GW 36" and the date of the sampling. WITHOUT IDENTIFICATION NUMBER AND DATE THE SAMPLE IS USELESS!

### 9.1.14 Magnetic resonance imaging (MRI)

Applicable in centers that have MRI as routine examination when the infants have reached full term (gw 37+0-42+0). Data from MRI will be filled in a specific protocol and added to the study file of the infant.

### 9.1.15 The end of the neonatal period

The follow up of the neonatal period ends at discharge from the hospital and/or the neonatal home care but not later than gestational week 44+0. The CRF should be completed, controlled and signed by any of the investigator at the actual site. Note that the result of the ROP examination shall be entered in CRF after gestational week 42+0.

Also, data on weight, length and head circumference from gestational week 36+0 +/2 days and from the day of discharge (or gestational week 44+0, whatever comes first) should be entered in the CRF.

#### 9.1.16 Follow up at 2 years of age

A follow up focusing on neurological development, growth and feeding problems will be performed at 2 years (± 3 months) of age (corrected). The follow up will be identical with the national follow-up program for extremely preterm infants that have been implemented in all paediatric clinics in Sweden (www.neo.barnlakarforeningen). In Sweden, all data from the national 2-year follow-up is entered in the Swedish Neonatal Quality Register (SNQ). Data in the SNQ will be used for analyses in the study. The 2-year follow-up includes a questionnaire that covers the period from the discharge from the hospital until 2 years of age. Cognitive function is tested with Bayley's test III by a psychologist and neurological disability by an experienced paediatrician and/or physiotherapist. Nurses coordinate the follow-up and meet with the family, measures weight, height and head circumference and complete a questionnaire about respiratory diseases, allergic diseases, feeding issues and socioeconomic factors.

#### 9.1.17 Follow up at 5.5 years of age

A follow up focusing on neurological development, growth and feeding problems will be performed at 5.5 years (± 3 months) of age. The follow up will be identical with the national follow-up program for extremely preterm infants that have been implemented in all paediatric clinics in Sweden (www.neo.barnlakarforeningen). In Sweden, all data from the national 5.5-year follow-up is entered in the Swedish Neonatal Quality Register (SNQ). Data in the SNQ will be used for analyses in the study. Cognitive function is tested with WPPSI-IV by a psychologist and neurological disability by an experienced paediatrician and/or physiotherapist. A physiotherapist will also perform the ABC Movement test. Nurses coordinate the follow-up and meet with the family, measures weight, height and head circumference and complete a questionnaire about respiratory diseases, allergic diseases, feeding issues and socioeconomic factors.

#### 9.1.18 Deaths

The sponsor shall be notified, if an infant dies before discharge from the neonatal ward. The information shall include study group and the cause of death. The CRF shall be filled in as completely as possible. That also includes diagnoses that would otherwise be filled in at discharge.

## *9.2 Assessment of efficacy*

### 9.2.1 Clinical variables

Definitions, please see variables in section 5. Data entered in the CRF and Nutrium (9.1.6)

### 9.2.2 Sample collection

It is optional for each study site to collect samples. The study site must have the logistics to take care of the samples and store them. Sufficient statistical power is usually reached with fewer cases in laboratory than clinical outcomes.

**Blood samples**

Blood samples are collected in EDTA tubes for plasma and separate pre-prepared tubes for masscytometry of blood cells (CyTOF) 1, 2 and 4 weeks (± 2 days) of age and at gestational week 33+0-33+6. The plasma and cells will be stored at -70°C.

**Faecal samples**
Faecal samples are collected at 1, 2, 3 and 4 weeks (± 2 days) of age and at gestational week 33+0-33+6 i sterile tubes. The sample is taken with a special spatula from the diaper. It is stored immediately in -20º C or -70º C. As soon as possible they should be transferred frozen to a -70º C freezer. PUT AS MUCH STOOL AS POSSIBLE IN THE TUBE (aim at 1 mL/"1cm"). It is an advantage if you use 2-3 tubes.

**Urine samples**
Urine is collected 1, 2, 3 and 4 weeks (± 2 days) of age and at gestational week 33+0-33+6 using cotton wool (first urine after 8.00 am), centrifuged and divided into 2-3 aliquots of about 1 mL of urine (placed in 1.5 ml sterile Eppendorf tube). The urine sample is stored in a freezer and picked up by the lab personnel to be stored at -70 ° C.

**Breast milk samples**
Breast milk samples are only collected if the mother has an excess of it. The sample is collected from the breast milk that the infant will be fed with at the actual day (after fortification): at 1, 2, 3 and 4 weeks (± 2 days) of age and at gestational 33+0-33+6 in sterile 10 ml tubes. The sample is stored in -20ºC. As soon as possible they should be transferred frozen to a -70º C freezer.

## *9.3 Assessment of safety*

### 9.3.1 Adverse events

Adverse events will be recorded in the CRF and in some cases they will be reported on as soon as they are detected, which is described in detail in section 13.

# 10. Concomitant therapy

Besides the supplementation of the breast milk fortifier and the samples collected in the trial, the participating infants will not be treated differently compared to infants outside the trial. No drug is contraindicated during the course of the study. Antibiotics and other relevant drugs are recorded in the CRF.

# 11. Concomitant diseases

All diseases/adverse events that the infant has before inclusion shall be recorded in the CRF since they might confound the results. All diseases or symptoms that the infant has during the study shall be recorded as adverse events in the CRF. Please see section 13.

# 12. Study requirements

## *12.1 Study product*

A description of the product ($H^2MF^{®}$), how it is stored, and how it is prepared is given in Appendix 17. In addition, the safety steps incorporated into the collection of the milk and in the production of the preparation also are indicated in that appendix.

## 12.2 Dose, administration, storage

Whether the infant has received the study products (breast milk fortifier according to 12.2.1 and 12.2.2), will be registered in Nutrium every day from the start to the end of the intervention period.

### 12.2.1 Active study group

A description of the products ($H^2MF^®$ and Prolact CR®), how they are stored, and how they are prepared is given in Appendix 17. Please, also see 9.1.5.

### 12.2.2 Control study group

The standard bovine protein-based milk fortifier used at the specific site will be prepared according the recommendation of manufacturer. Please, also see 9.1.5.

## 12.3 Storage

Storage of the $H^2MF^®$ and Prolact CR® is at -20º C. The expiration of the specific batch(es) provided are indicated on the bottles.

## 12.4 Study product accountability

Prolacta Bioscience, CA, USA, manufactures the active products. Details of product manufacture, distribution, stability, storage conditions and disposal of unused material are at Appendix 17. The standard bovine protein-based milk fortifier used at the specific site will be prepared according the recommendation of manufacturer.

Each study centre will be expected to record the use of investigational and standard care product in Nutrium.

## 12.5 Randomisation

This is a randomised trial. Randomisation will be based on the following stratification variables: primary enrolment site, gestational week (22+0-24+6 or 25+0-27+6), singleton/twin and gender. Because there are 4 stratification variables and not a particularly large study, an adaptive randomisation scheme will be used based on the method of minimisation. This will include a biased-coin randomisation scheme as needed in the adaptive scheme. [42] A specific computer program will be used to accomplish this scheme and the program will be carefully validated prior to incorporation in the study. The allocation will be concealed before inclusion, but after randomisation the study is not blinded.

When the infant fulfils the requirement of inclusion (please see section 9.1.1), a web-based randomisation service centre will be used: Randomize.net (Interrand Inc., Ottawa, Ontario, Canada). The patient identification number will be the consecutive number in the identification list at the study site, while the randomisation service centre will provide to which study group the infant shall be allocated. It is the patient identification number that should be used in the CRF and on samples etc. The allocation should be indicated in the identification list and also in the CRF immediately. To confirm eligibility inclusion and exclusion criteria will be checked for, including signed parental consent, before randomisation is done.

Twins will be stratified for as indicated above. They will be randomised together. Thus, they will get the same randomisation number and also the same nutritional protocol. Twin number one will get the suffix A and number 2 the suffix B (e.g. 101 A and 101 B). There will also be stratification for gender. Because of limitations of randomisation/stratification procedure, a twin pair will be considered having the same gender when the randomisation is made. Thus, enter the gender of the first-born twin in the program.

All study material of the included infant should be labelled with the patient identification number. The patient identification number will be five-digit and will start with 01001 in Umeå 02001 in Uppsala, 03001 in Linköping, 04001 in Göteborg, 5001 in Örebro, 6001 in Solna and 7001 in Huddinge.

# 13. Adverse Events

The morbidity of premature infants with extremely low birth weight is very high. Thus, a high incidence of serious and non-serious adverse events (SAE) unrelated to the study product could be expected in the participating infants. Also, mortality rate is high; please see background figures in section 3. Typical serious conditions affecting premature infants are listed below (13.1.2). In principle, none of these are unexpected.

As a consequence, special consideration must be given as to how the SAE is reported and followed up.

The use of $H^2MF^®$ is thoroughly studied and there no suggestions that $H^2MF^®$ will increase the incidence of AE, SAE, or death. Rather, previous research supports that feeding problems, NEC, sepsis and mortality is lower among infants receiving $H^2MF^®$ (please see section 3).

## *13.1 Definitions*

### 13.1.1 Non-serious adverse events

Non-serious adverse is reported in the CRF and will be analysed after all infants have completed the neonatal period.

### 13.1.2 Serious adverse events

| **Expected severe adverse events (SAE** |
|---|

- Severe infection such as pneumonia, sepsis or meningitis
- CMV infection, upper respiratory viral infection
- NEC, SIP and /or need of abdominal surgery
- Respiratory distress syndrome and BPD
- Intracranial bleeding, periventricular leucomalacia or hydrocephalus
- Lung bleeding
- Pneumothorax, pleural effusion
- Pulmonary hypertension
- PDA
- Retinopathy of prematurity (ROP)
- Death

These events do not have to be reported immediately (please see 13.2.1).

#### 13.1.3 Suspected unexpected serious adverse reaction (SUSAR)

A high incidence of serious adverse events (SAE) unrelated to the study product could be expected in the participating infants. In principle, none of these are unexpected. However, if there is a suspected unexpected serious adverse reaction (SUSAR), it should be reporting according to 13.2.2. Whether an infant has got a SUSAR or not is decided by the investigator or the attending physician at the study site.

## *13.2 Reporting serious adverse events*

#### 13.2.1 Reporting SAE

As a consequence of what is discussed above, SAE does not have to be reported immediately to the principal investigator or sponsor. Instead, interim safety analyses will be made according to a specific schedule - see 13.3 – to detect a potential increased incidence of SAE.

Thus, all SAE will be recorded in the CRF. In principle, they will be the same as the clinical morbidity variables addressed in paragraph 5.

#### 13.2.2 Reporting lethal cases and SUSAR

Investigator or the attending physicians at the study site are required to report SUSARs to the sponsor within 24 hours. A report form must be completed (Appendix 5) and promptly sent by mail or email to the sponsor. Phone number and address to the sponsor can be found at the research protocol first page and on the report form.

The sponsor is required to report SUSARs to the manufacturer, Prolacta Bioscience, and the Data and safety monitoring board (DSMB). Deaths are expected to occur, and the sponsors are not required to report these. The DSMB will, however, be contacted if there is an unexpected high mortality among in the participants.

## *13.3 Interim analysis*

Interim analysis will be carried out for safety analyses, please see section 13.4. Also, it will be made for the final sample size calculation, please see 7.1. The interim analyses will be made by an independent statistician.

### *13.4 Data and safety monitoring board (DSMB)*

An independent DSMB (Appendix 4), consisting of at least three members with at least one specialist in Neonatology and one Biostatistician, will have regular meetings to make interim safety analyses. Such an interim analysis shall be made after 50, 100 and 150 completed CRFs until discharge from the hospital. If any of the SAE is significantly more common ($p<0.05$) in the active than the control group, the DSMB will bring in all medical data on infants affected by this specific SAE. The DSMB will then assess the causality between the use of $H^2MF^®$ and the specific SAE in the affected infants. Based on this analysis the DSMB will decide if the trial can continue or not after consultation with the co-ordinating principal investigator and the sponsor. The DSMB may decide that it is ethically correct to pursue if the positive effects of the active intervention outweigh the SAE.

In case of a very strong effect of the active treatment, the study can be terminated prematurely based on a decision of the sponsor and the DSMB, if the primary outcome is significantly lower (with a significance level <0.001) in the $H^2MF^®$ than in the standard fortification group in the interim analysis made after 150 infants have been included [43,44]. If the significant level is ≥0.001 the study enrolment will continue (please see section 20).

# 14 Data collection

This is discussed in detail in section 14.1. In principle, all data from the neonatal period in the CRF originate from medical records of the patient. All materials will be labelled with the patient identification number. To avoid mixing of the participating infants (e.g. if they taking care of in the same room), the medical file, bed etc. will also be labelled with the patient identification number. Data from completed CRFs will be entered into the database continuously. A copy is sent to the study monitor. A study database will be created by a CRO and the data will subsequently be forwarded to the sponsor and the co-ordinating principal investigator. The entrance of data to the database will also be made by a CRO. Data from the 2 and 5.5 years follow up originate from the Swedish quality register.

All data on nutrition and other fluids will be entered daily in the Nutrium software (Nutrium AB, Umeå, Sweden) which is provided to all study sites. All nutritional data will be entered from birth to gestational week 34+0, when the intervention ends.
The data of infants included in the N-forte trial will be transferred to the database after the study has been completed.

## *14.1 Case Report Form (CRF)*

The CRF should be handled in accordance with instructions from the sponsor. All CRF should be filled out completely by examining personnel or the investigator/study nurse. The CRF are reviewed, signed, and dated by the investigator.

> The CRF is established at randomisation. The study nurse fills in all the background factors from the pregnancy and birth in collaboration with the investigators of the centre. The study nurse regularly checks the CRF to the patient medical record and completes the missing data. The CRF is kept together with medical file during the neonatal period and the infant´s study file after discharge from the clinic. In principle, all data collected in the study will be added in the CRF. Regarding the transfer to another clinic, please see 9.1.12. Upon discharge the CRF should be completed including evaluation of clinical diagnoses. The original CRF shall be submitted to the sponsor/PI after been monitored by the study monitor. One of the copies should be submitted to the data entrance company. One copy of the CRF should always be stored at the home clinic. A study database will be created by a CRO and the data will subsequently be forwarded to the sponsor and the co-ordinating principal investigator.

If a question in the CRF cannot be answered, this box shall be barred. When making changes or corrections, cross out the original entry with a single line, and initial and date the change. Do not erase, overwrite or use correction fluid or tape on the original.

.

If the CRF is sent by mail, it should be sent as a certified mail ("rekommenderat brev")

### *14.2 Data source*

In principle, all data in the CRF originate from data that also can be found in the medical records. Nutritional data on each case will transferred from Nutrium (Nutrium AB, Umeå, Sweden) to the sponsor in the end of the trial and added to the database. Likewise, the data from the 2- and 5.5-years follow up in the quality register SNQ (www.snq.se) will be transferred to the sponsor and added to the study database. There is a list of the data sources in the investigator file.

## 15 Data quality assurance

### *15.1. Study monitoring*

Site visits will be conducted by an authorized representative of the sponsor to inspect study data, subjects' medical records, and CRF in accordance with current Good Clinical Practices (GCP) and the respective local and national government regulations, internal SOP and guidelines. A monitor assigned by the CRO will conduct regular investigational centre visits for the purpose of study monitoring. The investigator will facilitate monitoring of the study and permit authorized representatives of the sponsor and international or national health authorities to inspect facilities and records relevant to this study. CRF data will be monitored during each visit according to the Study Monitoring Plan. All source data, subjects file, lab sheet etc. must be kept on file in accordance with GCP guidelines, i.e. for 10 years.

### *15.2. Study monitoring*

CRO will employ appropriate quality control measures in critical processes in data management (including data handling and data transfers) to ensure that data reported from the study are true and reliable.

## 16 Ethics

Research including ELBW infants is crucial in order to achieve knowledge of possible causes underlying the development of severe complication and finding preventive strategies in this age group. A growing literature supports the use of human milk-based protein fortifier in clinical practise (please see background, section 3). Intervention studies are needed to refute or confirm the suggested effect $H^2MF^®$. We do not perceive any major health hazard with the study design. $H^2MF^®$ is considered to be safe and could conceivably be used for simple, safe and effective prevention of severe complication in ELBW infants. Safety will also be monitored by a study specific DSMB.

Autonomy is very limited for this patient population. To adhere to ethical principles is therefore very important. Parents are the deputies of their child. A written informed consent is obtained from both parents prior to enrolment. Parents have the right to discontinue participation in the study at any time.

Blood sampling can cause pain and also affect the haemoglobin levels. Therefore, sampling is co-ordinated with other blood samples and only small amounts are collected at each time. Confidentiality is important and therefore only the patient identification number will be used to identify the infant in the study protocol and the test tubes.

Infants or their family have no specific benefit of participating in the trial besides to the possible positive effect of $H^2MF^®$ in the active group. Besides the supplementation of the breast milk fortifier and the samples collected in the trial, the participating infants will not be treated differently compared to infants outside the trial. The potential benefit for future ELBW infant, however, could be substantial. Finding an intervention that reduces NEC, sepsis and mortality would have a major impact of the well-being in this patient population. It would also a beneficial effect on health economics. Only randomised clinical trials will give sufficient evidence for a general recommendation of a new treatment.

The study is made in collaboration with the company Prolacta Bioscience producing $H^2MF^®$. The company also helps with randomisation and packaging. Without the aid from the company this trial would be difficult to conduct. The study, however is investigator-initiated, and the results are entirely owned by the principle investigator. None of the investigators have a financial interest in Prolacta Bioscience. Only excess breast milk produced by the donating mother that is beyond the consumptive needs of her nursing child are accepted. The donating mother signs informed consent form stating the rights and responsibilities of the donating mother as well as the payment for the provided breast milk.

The potential benefits are considered to outweigh the discomfort to the infants and their families. The study is conducted according to ICH/GCP guidelines and was approved by the regional ethical review board at Linköping University (Dnr 2018/193-31).

## 17. Informed consent

The information letter and the informed consent form can be found in Appendix 7.

If possible, the information can be given to the parents in several steps. Brief oral information can be given to the parents even before the birth if the child is expected to meet the inclusion criteria, please see 9.1.1.

An oral and written informed consent could preferably be obtained from the parents well before the oral feeds have reached 70 ml/kg/day in infants that are expected to fulfil the inclusion criteria, since they should have time enough to give a well-thought-out consent. Thus, in practice, the informed consent can be obtained already at the second and third day of life but not after the oral feeds have passed 100 ml/kg/day.

Once all the information has been provided, both parents/legal guardians will sign the inform consent form. If there is only one legal guardian, an informed consent from this person is sufficient. The attending physician shall note this in the medical record of the infant. If twins are enrolled, the parents/legal guardians shall sign an informed consent form for each twin. The original will be stored in the investigator file A copy is given to the parents.

# 18. Approval from research ethical board and other authorities

The application to the regional ethical review board at Linköping University has been approved before the start of the trial, (Dnr. 2018/193-31). A copy of the approved ethical application will be stored in the Investigator file at each study site. Blood, stool, urine and breast milk samples are included in the biobank (516) at Children and Women centre at the University Hospital in Linköping, Region Östergötland. There is a multi-centre agreement allowing the samples to be stored in the all centre in the trial. Application to the Medical Product Agency is not applicable since $H^2MF^{®}$ and standard fortifiers in this trial are considered as diet supplement and not classified as a drug but regarded as Food for Special Medical Purposes (FSMP). The regulation for FSMP is covered in SLVFS 2000:15. No application to the Swedish Food Agency is needed for clinical trials.

# 19. Study discontinuation

The sponsor may at any time terminate the study. In that case, the sponsor shall immediately inform all investigators. Furthermore, the sponsor should inform to the regional ethical review board at Linköping University and Prolacta Bioscience.

# 20. Statistical methods

With at least 101 infants in each group a reduction of the composite NEC, sepsis and mortality incidence of 47% in the control group (www.snq.se) to 28% in the active group could be detected at a two-sided 5% level of significance and with 80% power. To allow for a potential 10% dropout rate, a total of 222 patients will be enrolled (please see 7.1).

The following is an outline of the statistical approaches to be used. A more detailed evaluation will be incorporated into a Statistical Analysis Plan.

The primary basis for all analyses of the clinical outcome will use the intent-to-treat paradigm. This means that in this approach all randomised participants are included in the analysis regardless of their disposition in the study (including whether they actually adhered to the randomized nutritional protocol). In addition, a per protocol analysis will be conducted that will only include patients who appropriately complete the intervention. A further stratification in per protocol analysis will be if the onset of an outcome was before or after the start of the intervention. All analyses will be conducted at a two-sided 5% significance level. No adjustment will be made for multiple analyses for the clinical outcome.

Descriptive statistics will be computed for all quantitative outcome variables: mean ± standard deviation and median ± interquartile range. For qualitative (categorical) outcomes, proportions and/or percentages will be computed. The comparison of baseline variables such as gestational age, birth weight, gender and ethnicity/race will be based on the Wilcoxon rank-sum test for quantitative data and the chi-square test for homogeneity for categorical data.

The primary outcome variable is the occurrence of either (at least one event) NEC or sepsis and/or whether the infant dies. Since this is a binary variable, the two groups will be compared using the chi-square test for homogeneity. The CRF will be completed also in infants who end the intervention before gestational week 34+0. Decisive for the inclusion of the infant is that all episodes of the primary outcomes - NEC, sepsis and mortality- are filled in the CRF until the end of the neonatal follow-up at gestational week 36+0. The only reason that the infant is excluded from the intent-to-treat analysis is that parents deny the entry of these data into the CRF. A secondary evaluation of the primary endpoint will employ a multivariate adjustment model using logistic regression that will include appropriately selected covariates, e.g. birth weight, gestational age, gender, race and

the use of antenatal steroids. This will allow for the demonstration of the sturdiness of the primary analysis in the face of these adjustment variables.

There are a number of secondary outcome variables. Quantitative variables such as growth (weight velocity calculated as g/kg/day and change in z-score, length velocity calculated as cm/week and change in z-score and head circumference growth rate calculated as cm/week and change in z-score) will be compared between the study groups using the Wilcoxon rank-sum test. Any other non-censored quantitative variable will be evaluated in the same fashion. Right-censored time-to-event data (e.g. NICU or hospitalization time) will be evaluated by the method of Kaplan and Meier and compared between the groups using the log-rank test. In addition, secondary analyses will involve the use of various covariates that will be incorporated into the evaluation through the use of regression models (multiple linear, logistic, or Cox proportional-hazards depending on the type of data) and the covariates will include variables such as birth weight, gestational age, AGA versus SGA and other clinical variables to be decided upon prior to the initiation of data analysis. Qualitative variables such as the occurrence of BPD, ROP and IVH and the composite culture-proven sepsis and NEC will be compared between the groups using the chi-square test for homogeneity. Finally, number of days using TPN will be evaluated using the method of Ghandehari *et al.* [45]

A separate analysis of the cost data will be performed and this will use standard econometric methods to be defined. The statistical analyses of the laboratory data will also be defined in separate protocol for each analysis.

The study can be terminated prematurely because the primary outcome is significantly lower (with a significance level <0.001) in the $H^2$MF® than in the standard fortification group in the interim analysis made after 150 infants have completed the neonatal period (please see section 7.5) based on the Haybittle-Peto group sequential stopping boundary [43 44].

# 21. Financing and insurance

The study is investigator-initiated. The study, however, is made in collaboration with the company Prolacta Bioscience, CA, USA. The company will provide $H^2$MF® and Prolact CR® and also support randomisation and packaging. Prolacta Bioscience will also cover the cost for study nurses and monitoring. A financial agreement between the sponsor, each participating hospital and Prolacta Bioscience will be negotiated in accordance with national guidelines.

Participating patients will be insured via the patient injury act.

# 22. Final study report

The co-ordinating principal investigator is responsible of the final study report. The results of the primary research question will be published in international scientific peer-reviewed journals. They will also be reported at international congresses and national meetings for physicians and nurses. The public will be informed through press releases and media.

# 23. Retention of records

Records and documents pertaining to the conduct of this study, including CRF, source documents, consent forms, laboratory test results, and the investigator must retain medication inventory records, for at least 10 years according with GCP guidelines.

# 24. Confidentiality and publication

The Sponsor owns all the information obtained in the trial, and the information obtained by this study is confidential. Disclosure to third parties other than those noted below is prohibited. With the subject's permission, medical information may be given to his or her physician or other appropriate medical personnel in accordance with national regulations. Data generated by this study must be available for inspection upon request by representatives of the international, national and local health authorities, and the REB for each study site.

The data file with patient information must be encrypted and protected with a password.

The lawfulness of the processing of personal data in the study is based upon Article 6 (e) in the General Data Protection Regulation (GDPR) which states that the "processing is necessary for the performance of a task carried out in the public interest". Patient data must not be transferred to anyone without the approval by the co-ordinating principal investigator. In order to lawfully process special category data (also called sensitive data) a controller must identify a lawful basis under Article 6 and a separate condition for processing special category data under Article 9 in the General Data Protection Regulation. The separate condition that the N-Forte study uses to process special category data is article 9 (2) (j) in the General Data Protection Regulation which states that "processing is necessary for archiving purposes in the public interest, scientific or historical research purposes or statistical purposes in accordance with Article 89 (1) based on Union or Member State law which shall be proportionate to the aim pursued, respect the essence of the right to data protection and provide for suitable and specific measures to safeguard the fundamental rights and the interests of the data subject".

Before a CRF is transferred to a new hospital, the physician in charge has to make a review for the disclosure of patient data to the new hospital according to Chapter 25 Paragraph 1 of the Public access to secrecy act. It should be manifestly evident that the information may be disclosed without the patient or a person closely related to the patient being harmed. The same kind of review must be undertaken before the Nutrium data is transferred to the datafile in the end of the study by the investigator at the actual site.

The information obtained during this study may be made available to other researchers who are conducting similar studies and to international or national medical authority, with due respect to the scientific priority of the investigation and after consulting the co-ordinating Principal Investigator.
The intention is to publish the results in an international peer-reviewed journal. A copy of any manuscript (including meeting abstracts) reporting the primary outcomes in the study will be provided to Prolacta Bioscience for review and comments at least 30 days prior the expected date of submission to the intended publisher.

The trial is registered at ClinicalTrials.gov (NCT03797157)

# 25. References

1. EXPRESS. One-year survival of extremely preterm infants after active perinatal care in Sweden. *JAMA* 2009; **301**(21): 2225-33.
2. Breastfeeding AAoPSo. Breastfeeding and the use of human milk. *Pediatrics* 2012; **129**(3): e827-41.
3. Hair AB, Peluso AM, Hawthorne KM, et al. Beyond Necrotizing Enterocolitis Prevention: Improving Outcomes with an Exclusive Human Milk-Based Diet. *Breastfeed Med* 2016.
4. Roze E, Ta BD, van der Ree MH, et al. Functional impairments at school age of children with necrotizing enterocolitis or spontaneous intestinal perforation. *Pediatr Res* 2011; **70**(6): 619-25.

5. Ganapathy V, Hay JW, Kim JH. Costs of necrotizing enterocolitis and cost-effectiveness of exclusively human milk-based products in feeding extremely premature infants. *Breastfeed Med* 2012; **7**(1): 29-37.
6. Ganapathy V, Hay JW, Kim JH, Lee ML, Rechtman DJ. Long term healthcare costs of infants who survived neonatal necrotizing enterocolitis: a retrospective longitudinal study among infants enrolled in Texas Medicaid. *BMC Pediatr* 2013; **13**: 127.
7. Lucas A, Cole TJ. Breast milk and neonatal necrotising enterocolitis. *Lancet* 1990; **336**(8730): 1519-23.
8. el-Mohandes AE, Picard MB, Simmens SJ, Keiser JF. Use of human milk in the intensive care nursery decreases the incidence of nosocomial sepsis. *J Perinatol* 1997; **17**(2): 130-4.
9. Boyd CA, Quigley MA, Brocklehurst P. Donor breast milk versus infant formula for preterm infants: systematic review and meta-analysis. *Archives of disease in childhood Fetal and neonatal edition* 2007; **92**(3): F169-75.
10. Sullivan S, Schanler RJ, Kim JH, et al. An exclusively human milk-based diet is associated with a lower rate of necrotizing enterocolitis than a diet of human milk and bovine milk-based products. *J Pediatr* 2010; **156**(4): 562-7 e1.
11. Cristofalo EA, Schanler RJ, Blanco CL, et al. Randomized Trial of Exclusive Human Milk versus Preterm Formula Diets in Extremely Premature Infants. *J Pediatr* 2013.
12. Assad M, Elliott MJ, Abraham JH. Decreased cost and improved feeding tolerance in VLBW infants fed an exclusive human milk diet. *J Perinatol* 2016; **36**(3): 216-20.
13. Herrmann K, Carroll K. An exclusively human milk diet reduces necrotizing enterocolitis. *Breastfeed Med* 2014; **9**(4): 184-90.
14. Narayanan I, Prakash K, Bala S, Verma RK, Gujral VV. Partial supplementation with expressed breast-milk for prevention of infection in low-birth-weight infants. *Lancet* 1980; **2**(8194): 561-3.
15. Narayanan I, Prakash K, Gujral VV. The value of human milk in the prevention of infection in the high-risk low-birth-weight infant. *J Pediatr* 1981; **99**(3): 496-8.
16. Narayanan I, Prakash K, Murthy NS, Gujral VV. Randomised controlled trial of effect of raw and holder pasteurised human milk and of formula supplements on incidence of neonatal infection. *Lancet* 1984; **2**(8412): 1111-3.
17. Lucas A. Feeding Low Birthweight Infants. *Roberton NRC, Textbook of Neonatology 2nd edition Churchill Livingstone* 1992: 279-88
18. Lucas A, Fewtrell MS, Morley R, et al. Randomized outcome trial of human milk fortification and developmental outcome in preterm infants. *Am J Clin Nutr* 1996; **64**(2): 142-51.
19. Abrams SA, Schanler RJ, Lee ML, Rechtman DJ. Greater mortality and morbidity in extremely preterm infants fed a diet containing cow milk protein products. *Breastfeed Med* 2014; **9**(6): 281-5.
20. Lucas A, Morley R, Cole TJ. Randomised trial of early diet in preterm babies and later intelligence quotient. *BMJ* 1998; **317**(7171): 1481-7.
21. Singhal A, Cole TJ, Lucas A. Early nutrition in preterm infants and later blood pressure: two cohorts after randomised trials. *Lancet* 2001; **357**(9254): 413-9.
22. Singhal A, Farooqi IS, O'Rahilly S, Cole TJ, Fewtrell M, Lucas A. Early nutrition and leptin concentrations in later life. *Am J Clin Nutr* 2002; **75**(6): 993-9.
23. Singhal A, Fewtrell M, Cole TJ, Lucas A. Low nutrient intake and early growth for later insulin resistance in adolescents born preterm. *Lancet* 2003; **361**(9363): 1089-97.
24. Singhal A, Cole TJ, Fewtrell M, Lucas A. Breastmilk feeding and lipoprotein profile in adolescents born preterm: follow-up of a prospective randomised study. *Lancet* 2004; **363**(9421): 1571-8.
25. Fewtrell MS, Williams JE, Singhal A, Murgatroyd PR, Fuller N, Lucas A. Early diet and peak bone mass: 20 year follow-up of a randomized trial of early diet in infants born preterm. *Bone* 2009; **45**(1): 142-9.

26. Lucas A, Morley R, Cole TJ, Lister G, Leeson-Payne C. Breast milk and subsequent intelligence quotient in children born preterm. *Lancet* 1992; **339**(8788): 261-4.
27. Anderson JW, Johnstone BM, Remley DT. Breast-feeding and cognitive development: a meta-analysis. *Am J Clin Nutr* 1999; **70**(4): 525-35.
28. Isaacs EB, Fischl BR, Quinn BT, Chong WK, Gadian DG, Lucas A. Impact of breast milk on intelligence quotient, brain size, and white matter development. *Pediatr Res* 2010; **67**(4): 357-62.
29. Lewandowski AJ, Lamata P, Francis JM, et al. Breast Milk Consumption in Preterm Neonates and Cardiac Shape in Adulthood. *Pediatrics* 2016; **138**(1).
30. Lucas A, Lucas PJ, Chavin SI, Lyster RL, Baum JD. A human milk formula. *Early Hum Dev* 1980; **4**(1): 15-21.
31. Zhou J, Shukla VV, John D, Chen C. Human Milk Feeding as a Protective Factor for Retinopathy of Prematurity: A Meta-analysis. *Pediatrics* 2015; **136**(6): e1576-86.
32. Manzoni P, Stolfi I, Pedicino R, et al. Human milk feeding prevents retinopathy of prematurity (ROP) in preterm VLBW neonates. *Early Hum Dev* 2013; **89 Suppl 1**: S64-8.
33. Niklasson A, Albertsson-Wikland K. Continuous growth reference from 24th week of gestation to 24 months by gender. *BMC Pediatr* 2008; **8**: 8.
34. Bell MJ, Ternberg JL, Feigin RD, et al. Neonatal necrotizing enterocolitis. Therapeutic decisions based upon clinical staging. *Ann Surg* 1978; **187**(1): 1-7.
35. Gastmeier P, Geffers C, Schwab F, Fitzner J, Obladen M, Ruden H. Development of a surveillance system for nosocomial infections: the component for neonatal intensive care units in Germany. *J Hosp Infect* 2004; **57**(2): 126-31.
36. Jobe AH, Bancalari E. Bronchopulmonary dysplasia. *Am J Respir Crit Care Med* 2001; **163**(7): 1723-9.
37. Prematurity TICftCoRo. The International Classification of Retinopathy of Prematurity revisited. *Arch Ophthalmol* 2005; **123**(7): 991-9.
38. Papile LA, Burstein J, Burstein R, Koffler H. Incidence and evolution of subependymal and intraventricular hemorrhage: a study of infants with birth weights less than 1,500 gm. *J Pediatr* 1978; **92**(4): 529-34.
39. de Vries LS, Eken P, Dubowitz LM. The spectrum of leukomalacia using cranial ultrasound. *Behav Brain Res* 1992; **49**(1): 1-6.
40. Bayley N. Bayley Scales of Infant and Toddler Development. *3rd ed, San Antonio* 2006: TX: Harcourt Assessment Inc.
41. Gould AL. Interim analyses for monitoring clinical trials that do not materially affect the type I error rate. *Stat Med* 1992; **11**(1): 55-66.
42. Pocock SJ, Simon R. Sequential treatment assignment with balancing for prognostic factors in the controlled clinical trial. *Biometrics* 1975; **31**(1): 103-15.
43. Pantiadosi S. Clinical trials: a methodological perspective, 2nd ed, p 384-386. John Wiley & sons, New Jersey, 2005. .
44. Haybittle JL. Repeated assessment of results in clinical trials of cancer treatment. *Br J Radiol* 1971; **44**(526): 793-7.
45. Ghandehari H, Lee ML, Rechtman DJ, Group HMS. An exclusive human milk-based diet in extremely premature infants reduces the probability of remaining on total parenteral nutrition: a reanalysis of the data. *BMC Res Notes* 2012; **5**: 188.

# 25. Appendix

## *Appendix 1 –Agreement between sponsor and investigator*

**PROTOCOL SIGNATURE PAGE**

By signing below, the Investigator agrees to adhere to the protocol as outlined and agrees that any changes to the protocol must be approved by the sponsor prior to seeking approval from the Research ethical board.
This study will be conducted in accordance with current ICH regulations, the Declaration of Helsinki, and local ethical and legal requirements.

Sponsor´s Signature: ________________________________________

Printed Name: ________________________________________________

Date: _______________________________________________________

Co-ordinating principal Investigator's Signature: ________________________________________

Printed Name: ________________________________________________

Date: _______________________________________________________

Investigator's Signature: ________________________________________

Printed Name: ________________________________________________

Date: _______________________________________________________

Clinical Director Signature:_____________________________________________
(verksamhetschef)

Printed Name:

Date:_______________________________________________________

Please submit a signed original of this page to the sponsor and add copy to the investigator file at the study site.

## *Appendix 2 –List of contributing neonatal units and their contact persons*

**Neo-IVA, H.K.H Kronprinsessan Victorias Barn- och ungdomssjukhus i Linköping**

Principal investigator:
Thomas Abrahamsson, Neonatolog, Överläkare, (principal investigator)
thomas.abrahamsson@regionostergotland.se

Co-investigators
Ingela Danielsson, Neonatolog, Överläkare
Ingela.danielsson@regionostergotland.se

Georg Bach-Jensen, Barnläkare, avdelningsläkare
Georg.Bach.Jensen@regionostergotland.se

Study nurse: Samantha Karlsson (Samantha.Karlsson@regionostergotland.se), Caroline Törnqvist, dietitian (caroline.tornqvist@regionostergotland.se). Study cell phone: +46 72 2170317

Milk kitchen:, Carin Klang (carin.klang@regionostergotland.se), Kerstin Persson,

(kerstin.persson@regionostergotland.se)

Address:
H.K.H Kronprinsessan Victorias Barn- och ungdomssjukhus, plan 15
Universitetssjukhuset i Linköping
581 85 Linköping
Tel: 010-1030000 vx

**Neonatalavdelningen, Barn- och ungdomssjukhuset, Länssjukhuset Ryhov**

Local investigator:
Fredrik Ingemansson, Neonatolog, Överläkare
fredrik.ingemansson@rjl.se
Other physicians: Martin Sturm (martin.sturm@rjl.se)

Study nurse: Sara Elomri (sara.elomri@rjl.se)

Milk kitchen: Susanne Nilsson (susanne.nilsson@rjl.se), Eva Liljenberg (eva.liljenberg@rjl.se)

Address:
Länssjukhuset Ryhov
551 85 Jönköping
Tel: 036-32 10 00 vx

**Neonatalavdelningen, Barn- och ungdomskliniken, Vrinnevisjukhuset**

Local investigator:
Erik Wejryd, Neonatolog Överläkare
erik.wejryd@regionostergotland.se

Study nurse: Sara Wikström (sara.wikstrom@regionostergotland.se); Lisen Strandberg (lisen.strandberg@regionostergotland.se)

Milk kitchen: Sara Wikström (sara.wikstrom@regionostergotland.se) Lisen Strandberg (lisen.strandberg@regionostergotland.se), Matilda Klevelid (Matilda.klevelid@regionostergotland.se)

Address:
Gamla Övägen 25
603 79 Norrköping
Tel: 010-1040000 vx

**Neonatalavdelningen, Barn- och ungdomskliniken, Västerviks sjukhus**

Local investigator:
Maneck Bhiladvala, Neonatolog, Överläkare
maneck.bhiladvala@ltkalmar.se

Study nurse: Pia Winnes Jonsson pia.jonsson@regionkalmar.se

Milk kitchen: Kicki Kotanidou (kicki.kotanidou@ltkalmar.se)

Address:
Östra Kyrkogatan 48
593 33 Västervik
Tel: 0490-86000 vx

**Neonatalavdelningen, Barn- och ungdomskliniken, Länssjukhuset i Kalmar**

Local investigator:
Henrik Pettersson, Barnläkare, Överläkare
henrik.petersson3@regionkalmar.se

Study nurse: Anette Sjöström (anette.sjostrom@regionkalmar.se), Madeleine Törnqvist (madeleine.tornqvist@regionkalmar.se) Jessica Brorsson (jessica.brorson@regionkalmar.se)

Milk kitchen: Evy Gustafsson (evy.gustafsson@ltkalmar.se)

Address:
Lasarettsvägen
39185 Kalmar
Tel: 0480-81000 vx

**Neonatalavdelning 95F, Akademiska Barnsjukhuset Uppsala**

Principal investigator:
Fredrik Ahlsson, Neonatolog, Överläkare,
Fredrik.ahlsson@kbh.uu.se

Study nurse: Emilia Biskop (emilia.biskop@akademiska.se); Therese Paulsdotter (therese.paulsdotter@akademiska.se)

Milk kitchen: Carina Engström (carina.engstrom@akademiska.se)

Address:
Avd 95F
Akademiska Barnsjukhuset
751 85 Uppsala
Tel: 018-6110000 vx

**Avdelning 11 Neonatologi, Centralsjukhuset i Karlstad**

Local investigator:
Eva Albinsson, Barnläkare, Överläkare
Eva.Albinsson@liv.se

Study nurse: Lovisa Pettersson, Lovisa.Pettersson@regionvarmland.se Lina Karlsson, Lina.M.Karlsson@regionvarmland.se

Milk kitchen: : Katarina Orre, Katarina.orre@regionvarmland.se
Renee Magnusson, renee.smedman@regionvarmland.se
Maria Jarl maria.jarl@regionvarmland.se

Address:
Centralsjukhuset
Rosenborgsgatan
651 85 Karlstad
Tel: 054-615264

**Avdelning 69, Barn- och ungdomskliniken, Västerås sjukhus**

Local investigator:
Urban Rosenqvist, Barnläkare, Överläkare
urban.rosenqvist@regionvastmanland.se

Study nurse: Melina Edren, melina.edren@regionvastmanland.se

Milk kitchen: Annsofi Rosenqvist, ann-sofie.rosenqvist@regionvastmanland.se

Address:
Avdelning 69
Västmanlands Sjukhus Västerås
721 89 Västerås
Tel: 021-173000 vx

**Neonatalavdelningen, Barn- och ungdomskliniken, Universitetssjukhuset Örebro läns landsting**

Local investigator:
Eleni Evangelidou, Neonatolog, Specialist läkare
eleni.evangelidou@regionorebrolan.se; tel work : 01960-21723, tell mobil: 0768867267

Study nurse: Martina Carlsen, martina.carlsen@regionorebrolan.se Tel. 070-4213862

Milk kitchen: Petra Eriksson, petra.eriksson@regionorebrolan.se Tel. 070-3054851
Karin Löfving, karin.lofving@regionorebrolan.se Tel. 070-7702121

Address:
Neonatalavdelningen
Universitetssjukhuset Örebro
701 85 Örebro
Tel: 019-602 17 00

**Neonatalavdelningen, Barn- & Ungdomsmedicinska kliniken, Falu lasarett**

Local investigator:
Maria Degerman. Barnläkare
maria.degerman@ltdalarna.se

Study nurse:
Anna Söderlund Anna.b.Soderlund@ltdalarna.se
Carina Högberg carina.hogberg@regiondalarna.se

Milk kitchen:

Dietician: Caroline Hjort, Caroline.hjort@ltdalarna.se, Ulla Cederholm, Ulla.cederholm@ltdalarna.se

Address:
Neonatalavdelningen
Falu lasarett
79182 Falun
Tel: 023-492000 vx

**Neonatalavdelningen, Barn- och ungdomskliniken, Mälarsjukhuset, Eskilstuna**

Local investigator:
Ferial Mohamad, Överläkare
Ferial.mohamad@regionsormland.se
Study nurse:
Cecilia Persson, Cecilia.Persson@regionsormland.se .Ellinor Sundman, Ellinor.sundman.liberg@regionsormland.se. Victoria Larsson, victoria.larsson@regionsormland.se

Milk kitchen

Anne Christenssen, anne.christensen@regionsormland.se. Josefine Sandström, josefine.sandstrom@regionsormland.se. Ida Lundin, ida.lundin@regionsormland.se

Address:
Barn- och ungdomskliniken, Mälarsjukhuset
63188 Eskilstuna
Tel 016-105464

**Neonatalavdelningen 107A, Barn- och Ungdomssjukvården Region Gävleborg**

Local investigator:
Sofia Arwehed, Barnläkare, Neonatolog, Överläkare,
sofia.arwehed@regiongavleborg.se

Study nurse: Anna Wielbass, anna.wielbass@regiongavleborg.se
Kerstin Widahl, kerstin.widahl@regiongavleborg.se
Milk kitchen: no one specific so far
Stina Svensson, stina.svensson@regiongavleborg.se

Address:
Barn- och ungdomssjukvården Region Gävleborg,
Avd 107A

Region Gävleborg
801 87 Gävle
Tel: 026-15 40 00 vx

**Neonatal-IVA, Barn- och Ungdomscentrum, Norrlands Universitetssjukhus, Umeå**

Principal investigator:
Magnus Domellöf, Professor, Överläkare ,
magnus.domellof@umu.se

Study nurse: Marie Lindvall (Marie.Lindvall@regionvasterbotten.se)
Gunilla Strinnholm (gunilla.strinnholm@regionvasterbotten.se)

Dietician: Inger Öhlund (Inger.ohlund@umu.se)

Milk kitchen:
Susanne Jacobsson
Elin Bergman (Elin.Bergman.Sundkvist@regionvasterbotten.se)

Address:
Neonatal-IVA
Barn- och Ungdomscentrum, Norrlands Universitetssjukhus
901 85 Umeå
Tel: 090-785 2128

**Avdelning 56 Nyfödda, Sunderby Sjukhus, Luleå**

Local investigator:
Ingela Heimdahl
ingela.heimdahl@norrbotten.se

Study nurse:

Lena carina Sandström lena.tiger-sandstrom@norrbotten.se
Margit Eriksson margit.eriksson@norrbotten.se

Milk kitchen: Annika Pettersson, annika.pettersson@norrbotten.se

Address:
Sunderby sjukhus
Avdelning 56 Nyfödda
971 80 Luleå
Tel:

**Neonatalavdelningen, Barn- och Ungdomskliniken, Östersunds sjukhus**

Local investigator:
Kari Arimaa, barnläkare
Kari.arimaa@regionjh.se

**Study nurse:**)

**Milk kitchen: Monica Landén(monica.landen@regionjh.se)**

Address:
Neonatalavdelningen
Barn- och Ungdomskliniken
831 83 Östersund
Tel: 063 153000

**Neonatalavdelning 45, Sundsvalls sjukhus**

Principal investigator:
Jens Bäckström
jens.backstrom@rvn.se,

Study nurse: Teres Viltlycke teres.viltlycke@rvn.se

Milk kitchen: Maria Thelberg maria.thelberg@rvn.se
Josefine Sätterström josefine.satterstrom@rvn.se

Address:
Neonatalavdelning 45
Sundsvalls sjukhus
851 86 Sundsvall
Tel: 070-4950701 and 060-155115.

**Neonatalverksamheten, Drottning Silvias barn- och ungdomssjukhus, SU/Östra, 416 85 Göteborg, Sweden**

Principal investigator:
Anders Elfvin Neonatolog, Överläkare,
anders.elfvin@vgregion.se

Study nurse: Anna-Lena Fransson
anna-lena.fransson@vgregion.se

Milk kitchen: Avd 316: Kicki Bergström christina.s.bergstrom@vgregion.se, Avd 309: Linnea Löfström linnea.lofstrom@vgregion.se

Dietician: Karina Eftring (karina.eftring@vgregion.se)

Address:
Drottning Silvias barn- och ungdomssjukhus
SU/Östra
416 85 Göteborg
Phone vx: 031-342 10 00

**Neonatalavdelningen, Barn- och ungdomskliniken, Södra Älvsborgs sjukhus, Borås**

Local investigator:
Mirjana Janes- Nakic, Neonatolog, Specialistläkare mirjana.janes-nakic@vgregion.se

Study nurse: Carola Edvardsson carola.edvardsson@vgregion.se

Milk kitchen: Felicia Johansson felicia.johansson@vgregion.se

Address:
Södra Älvsborgs sjukhus
Brämhultsvägen 53
501 82 Borås
Phone vx: 033- 616 10 00

**Neonatalavdelningen, Barn- och ungdomskliniken, Norra Älvsborgs Länssjukhus, Trollhättan**

Local investigator:
Peder Helmerson, Neonatolog, Överläkare peder.helmersson@vgregion.se

Study nurse: Marita Karlsson marita.karlsson@vgregion.se

Milk kitchen: Emma Johansson

Address:
Norra Älvsborgs Länssjukhus
416 85 Trollhättan
Phone vx: 010 – 435 00 00

**Neonatalavdelningen, Barn- och ungdomskliniken, Skaraborgs sjukhus, Skövde**

Local investigator:
Ivo Kaltchev , barnläkare
ivo.krassimirov.kaltchev@vgregion.se

Study nurse:
Veronica Norlin veronica.norlin@vgregion.se
Jenna Karppinen jenna.karppinen@vgregion.se

Ninni Ståhl kristina.x.stahl@vgregion.se

Milk kitchen.
Elisabeth Westberg elisabeth.westberg@vgregion.se
Yvonne Landqvist yvonne.landqvist-junestal@vgregion.se
Mjölkkök skas.neonatal.nutritionsgruppen@vgregion.se.
Malin Alexandersson malin.alexandersson@vgregion.se

Address:
Skaraborgs sjukhus Skövde
541 85 Skövde
Phone vx: 0500- 43 10 00

**Neonatalavdelningen, Astrid Lindgrens Barnsjukhus, Solna, Stockholm**

Principal investigator at Region Stockholm:
Lars Navér, Docent, Överläkare
lars.naver@sll.se

Local investigator: Elena Palleri elena.palleri@sll.se, Anna Gudmundsdottir anna.gudmundsdottir@sll.se, Agnes Linnér agnes.linner@sll.se, Ewa Henckel ewa.henckel@sll.se
Study nurse: Amie Lindberg amie.lindberg@sll.se
Emma Eriksson emma.bi.eriksson@sll.se

Milk kitchen: Sofia Söderquist-Kruth (dietist)
Ami Martinez amerinel.martinez@sll.se

Adress:
Karolinska Universitetssjukhuset Solna
F8:85. Nutritionsrummet.
Eugeniavägen 23
171 76 Stockholm
Phone: Koordinator tel: 08-517 74092

**Neonatalavdelningen, Astrid Lindgrens Barnsjukhus, Huddinge, Stockholm**

Local investigator: Ewa Henckel ewa.henckel@sll.se, Anna Gudmundsdottir anna.gudmundsdottir@sll.se
Study nurse: Amie Lindberg amie.lindberg@sll.se, 08-585 815 43, 0725-955769
Milk kitchen: Karin Larsson, Anki Jonsson-Sjöberg

Adress:

Karolinska Sjukhuset Huddinge

PO sjuka nyfödda barn, K76-K78.

Hälsovägen

141 52 Huddinge

**Neonatalavdelningen, Astrid Lindgrens Barnsjukhus, Danderyd, Stockholm**

Local investigator: Elena Palleri elena.palleri@sll.se,
Study nurse: Gordana Printz tel:070-4439405, Amie Lindberg amie.lindberg@sll.se, 08-585 815 43, 0725-955769,
Milk kitchen: Malin Tilja malin.tilja@sll.se
Address:
Armbågsvägen 3
Hus 12, plan 5, Nutritionsrummet
182 88 Stockholm
Phone vx: 0812355513

**Neonatalavdelningen, Sachsska Barnsjukhuset, Södersjukhuset, Stockholm**

Local investigator: Josefin Lundström josefin.lundstrom@sll.se
Study nurse: Emylie kålerid, emylie.karelid@sll.se
Milk kitchen:

| | |
|---|---|
| Ingela Sandström | ingela.sandstrom@sll.se |
| Charlotte Martinsson | lottiemartinsson@hotmail.com |
| Kerstin Larsdotter | kerstin.larsdotter@sll.se |

Adress:
Sachsska barn och ungdomssjukhuset/Södersjukhuset
Neonatalavdelningen Avd 71/ Nutritionsrummet.
Sjukhusbacken 10,
118 83 Stockholm
Phone vx: 08-6161271, 08-616 1261, 070-4439405

## *Appendix 3 –Agreement with the head of the clinic (this was included in the ethical application)*

# Intyg om att erforderliga resurser finns för att garantera forskningspersonernas säkerhet.

Härmed intygas att erforderliga ekonomiska, strukturella och personella resurser finns tillgängliga på denna klinik för att garantera forskningspersonernas säkerhet vid genomförandet av projektet ”Nordic study on human milk fortification in extremely preterm infants (N-Forte)”

__________________________________________________

Verksamhetschefs signatur Datum

__________________________________________________

Namnförtydligande

__________________________________________________

Center/klinik

## *Appendix 4 – Data and safety monitoring board (DSMB)*

The member of the DSMB

Baldvin Jonsson, Docent, Överläkare, Astrid Lindgrens Barnsjukhus, Stockholm (chairman).
Baldvin.jonsson@karolinska.se

Mats Hammar, Professor, Överläkare, Kvinnokliniken, Universitetssjukhuset i Linköping.
mats.hammar@liu.se

Mats Fredrikson, biostatistician, Forum Östergötland, Universitetssjukhuset i Linköping.
mats.fredrikson@liu.se

## *Appendix 5 –Reporting of SUSAR*

Investigator or the attending physicians at the study site are required to report SUSARs to the Co-ordinating Principal investigator within 24 hours. This report form must also be completed and promptly sent by mail or email to the principal investigator.

| Name of the patient | Patient personal number | Reserve number (if appl) | Patient identification number |
|---|---|---|---|

**SUSAR:**

| Date | Time | Where? |
|---|---|---|

Send to:

Co-ordinating Principal investigator:
Thomas Abrahamsson,
H.K.H Kronprinsessan Victorias Barn- och ungdomssjukhus, plan 15
Universitetssjukhuset
581 85 LINKÖPING
010 – 103 00 00 (vx)
mob: +46-70956 68 15
email: thomas.abrahamson@regionostergotland.se

## *Appendix 6 –Study information to the parents*

# Vill ni delta i N-Forte studien, där vi jämför två olika proteinberikningar av bröstmjölk?

**Bästa föräldrar!**
Hur kan tillmatningen av för tidigt födda barn förbättras? Detta vill vi undersöka i en forskningsstudie där vi jämför två olika näringsberikningar av bröstmjölken. Den ena är baserad på komjölk och den andra på human bröstmjölk. Syftet med studien är att se om risken för att få blodförgiftning och inflammation i tarmen kan minska om det nödvändiga proteintillskottet som för tidigt födda barn behöver kommer från donerad bröstmjölk istället för komjölk.

**Varför vill vi göra den här studien?**
Ett barn som föds extremt för tidigt är mycket känsligt och kan drabbas av allvarliga komplikationer under vårdtiden. Den medicinska utvecklingen har gått framåt och gjort att de flesta av dessa barn ändå överlever och klarar sig bra.

Barnens tillmatning och tillväxt är en viktig utmaning. Ju bättre vi lyckas med detta, desto bättre blir barnets hälsa och utveckling – både på kort och på lång sikt. Målet är att ge dessa små barn förutsättningar att växa lika bra som de skulle ha gjort inuti livmodern. Vi vet att bröstmjölk är den bästa födan, även för prematura barn. Därför ger vi alltid någon milliliter mjölk redan första dygnet och ökar sedan mängden successivt. Efter ett antal veckor kan barnet helt och hållet matas med bröstmjölk och behöver inte dropp. I första hand ges mammans egna utpumpade mjölk. Om den inte räcker till ger vi bröstmjölk som har donerats till en bröstmjölksbank, så att barnet inte behöver få vanlig bröstmjölksersättning.

Barn som föds extremt för tidigt har ett större näringsbehov än barn som föds efter vanlig graviditetslängd. Därför tillsätts rutinmässigt extra berikning (som innehåller protein, mineraler och vitaminer) till bröstmjölken för att de skall växa så bra som möjligt. Den berikning som används på sjukhusen idag är gjorda av komjölk eftersom det inte har funnits några alternativ.

Det har nu utvecklats en berikning om kommer från human bröstmjölk, d.v.s. bröstmjölk som donerats av andra mammor och det finns en del forskningsresultat som tyder på att denna ”humana” bröstmjölksberikning kan ha positiva hälsoeffekter. Vi vill nu undersöka om risken för infektion och svår tarminflammation (NEC) minskar om de får denna berikning istället för den traditionella berikningen som gjorts av komjölk.

**Vilka kan vara med i studien?**
Alla barn som är födda i graviditetsvecka 22+0 till 27+6 som inte har någon allvarlig bukåkomma, medfödd sjukdom eller missbildning. Barnet skall inte fått något proteintillskott i bröstmjölken innan det går med i studien.

**Hur går studien till?**
Barnet skall inkluderas i studien innan vi börjar ge extra berikning i bröstmjölken, vilket oftast brukar ske vid 4-21 dagars ålder, och det följs sedan hela vårdtiden under nyföddhetsperioden. Vi kommer även att göra en uppföljning när ert barn är två år gammalt samt vid 5,5 års ålder. Dessa uppföljningar görs i samband med den ordinarie uppföljningen som görs på alla för tidigt födda barn.

De barn som deltar i studien delas in i två grupper. I ena gruppen kommer barnen att få en berikning som baseras på donerad bröstmjölk ($H^2MF^®$) fram till graviditetsvecka 34+0. Om barnet behöver extra energi kommer ett fettillskott (ProlactCR®) som också är baserat på bröstmjölk att ges till barnen i den gruppen. I den andra gruppen kommer barnen få det berikning baserat på komjölk som är standard på avdelningen. Vid behov av extra fett kommer de få det fettillskott som är standard på avdelningen. Barnen kommer att lottas slumpmässigt till en av de två grupperna så att hälften av barnen får traditionell berikning och hälften får $H^2MF^®$. På så sätt kan vi jämföra om den nya berikningen är bättre än den nuvarande standardbehandlingen. Barnen i de båda grupperna kommer i övrigt att behandlas på precis samma sätt.

Under vårdtiden kommer uppgifter om barnets tillmatning, tillväxt, behandlingar och symptom samt uppgifter om mamman som är viktiga för barnets hälsa samlas in från journalen och nutritionsprogram för att kunna tolka resultaten i studien rätt. Prover kommer att tas för att undersöka vilka mekanismer som kan ligga bakom förbättrad tillväxt, tarmfunktion och infektionsförsvar. Blodprov kommer att tas på barnet vid fyra tillfällen under vårdtiden i samband med att blodprov ändå tas av andra skäl. Dessutom kommer prov att tas på utpumpad bröstmjölk från mamma samt prov på avföring och urin från barnet. Dessa prover kommer att frysas in för analyser i framtiden. De kommer först ingå i vårdgivarens biobank och sedan flyttas till Barn- och kvinnocentrums biobank 516 i Region Östergötland, Linköping. Ni kan när som helst begära att dessa prover förstörs utan att ange orsak.

Förutom provtagningen och studieprodukten kommer barnen inte att behandlas annorlunda än barn som inte deltar i studien.

**Är det farligt?**
Det nya kosttillskottet ($H^2MF^®$) och fettillskottet (ProlactCR®) har testats i flera studier på extremt för tidigt födda barn tidigare. Det finns inga rapporter om förekomst av ett ökat antal infektioner eller andra allvarliga biverkningar. För säkerhets skull kommer vi ändå kontrollera barnen noga.

**Kan man lämna studien?**
Ett deltagande i studien är helt frivilligt och om ni samtycker till deltagande men sedan ändrar er kan ni när som helst ta ert barn ur studien. Ni behöver inte berätta varför. Vi kommer i så fall att fråga er om vi även efter avbrytandet får registrera uppgifter om barnets tillväxt och hälsotillstånd. Om ni väljer att avbryta ert barns studiedeltagande kommer det inte att påverka ert barns fortsatta vård. Ingen ersättning utgår för deltagande i studien. Patientförsäkringen gäller för ert barn precis som för all vård.

**Vilka frågor vill vi få svar på?**

- Kan $H^2MF^®$ minska förekomsten av allvarliga sjukdomar såsom blodförgiftning och inflammation i tarmen jämfört med komjölksbaserad proteinberikning?
- Hur lång tid tar det innan barnet klarar av att få fulla mål med bröstmjölk?
- Hur påverkas barnets tillväxt och utveckling?
- Vilka faktorer kan ligga bakom effekten av $H^2MF^®$?
- Vilka andra mekanismer ligger bakom allvarliga komplikationer, dålig tillväxt och mag- och tarmfunktion

**Vad gör vi med resultatet?**
Studiens resultat kommer att redovisas i internationella vetenskapliga tidskrifter. Om det visar sig att $H^2MF^®$ minskar risken för komplikationer för det för tidigt födda barnet, är avsikten att kunna erbjuda denna berikning till alla för tidigt födda barn. Ni kommer att få information om resultaten via brev.

**Behandling av personuppgifter**
Uppgifterna tas ifrån patientjournalen och används bara för att besvara de frågor som vi har beskrivit ovan. De hanteras enligt EU´s dataskyddsförordning. Den lagliga grunden för hanteringen är dataskyddförordningens stöd för behandling av uppgifter för forskningsändamål. All data behandlas gruppvis och inga obehöriga kan ta del av dem. De måste sparas i en databas i minst 10 år och måste sparas längre om de vetenskapliga analyserna fortfarande pågår. Region Östergötland, Region Uppsala, Region Västerbotten, Västra Götalandsregionen och Stockholms läns landsting har ett gemensamt personuppgiftsansvar för all data i den sparade datafilen. Vid behov kan ni kontakta dataskyddsombudet i Region Östergötland på adressen Region Östergötland, Dataskyddsombud, 581 91 Linköping, eller via e-post dataskyddsombud@regionostergotland.se. På studieprotokoll, provrör och i databasen anges inte namn eller personnummer utan bara en särskild kod som är unik för ert barn. För att vara säker på att alla uppgifter rapporteras in på ett korrekt sätt kommer en kvalitetsgranskning att göras. En oberoende person kan då komma att jämföra de formulär som fylls i med barnets journal. Röntgenbilder kan även komma att skickas för en extra bedömning till röntgenspecialister i annat landsting. I forskningssyfte kan forskare från andra universitet i Sverige och utomlands ta del av data i databasen. Det gäller även det USA-baserade företaget Prolacta som producerar kosttillskottet H$^2$MF®. Ni har rätt att få ta del av det särskilda avtal som krävs för att data skall överföras till ett företag i USA. Det kanadensiska företaget Interrand får även enstaka uppgifter som behövs när de hjälper oss med lottningen till vilken grupp barnet skall höra (födelseort, om barnet föddes före graviditetsvecka 25+0, könstillhörighet, och om det är en tvilling eller inte). Inte heller till Interrand skickas namn eller personnummer. Alla personer som tar del av data har tystnadsplikt. Alla resultat som publiceras i vetenskapliga tidskrifter kommer presenteras på gruppnivå så inget enstaka barn kan identifieras. Ni har rätt att kostnadsfritt få ta del av de uppgifter om er som hanteras i studien och vid behov få eventuella fel rättade. För att säkerställa studieresultatets kvalitet kan, enligt gällande lagar och förordningar, data insamlade i forskningsstudier inte alltid raderas på er begäran. Eventuella klagomål på hanteringen av personuppgifterna kan ges till Datainspektionen

Om Ni har några ytterligare frågor eller vill ha mer information, tag då gärna kontakt med någon av oss.

För projektet,

| | |
|---|---|
| [ Namn och titel ansvarig läkare] | [Namn och titel ansvarig ssk ] |
| [Arbetsplats/klinik ]<br>[ Tel nr ]<br>[E-mail ] | [Arbetsplats/klinik ]<br>[ Tel nr ]<br>[E-mail ] |

## *Appendix 7 –Informed consent form*

# Samtycke till deltagande

Undertecknande målsmän samtycker härmed till att vårt barn deltar i N-Forte studien, som undersöker om det bröstmjölksbaserade proteintillskottet $H^2MF^®$ minskar risken för svåra komplikationer och förbättrar uppfödning och tillväxt hos extremt för tidigt födda barn jämfört med komjölksbaserat proteintillskott. Vårt barn kommer att antingen få $H^2MF^®$ eller det komjölksbaserade proteintillskott som är standard på avdelningen.

Vi har tagit del av den skriftliga informationen. Vi har haft möjlighet att ställa frågor och vi har fått tillfredställande svar.
Vi godkänner att en utomstående person kan komma att granska studiehandlingarna och jämföra med uppgifter i patientjournalen.

**Angående lagring av biologiska prover**
Vi har informerats och samtycker till att biologiska prover (blod, bröstmjölk, urin och avföring) får sparas nedfrysta i biobank för framtida vetenskapligt bruk inom det område som detta projekt avser. Vi kan när som helst begära att dessa prover förstörs utan att ange orsak.

Vi är medvetna om att deltagandet är helt **frivilligt** och att vi har möjlighet att när som helst avbryta studien utan att ange något skäl.

| | |
|---|---|
| ……………………………………………<br>Barnets personnummer | ……………………………………………<br>Barnets efternamn och ev förnamn |
| ……………………………………………<br>Namnteckning vårdnadshavare 1 | ……………………………………………<br>Namnteckning vårdnadshavare 2 |
| ……………………………………………<br>Ort och datum | ……………………………………………<br>Ort och datum |
| ……………………………………………<br>Textat namn | ……………………………………………<br>Textat namn |
| ……………………………………………<br>Telefon hem mobil | …………………………………………….<br>Telefon hem mobil |
| …………………………………………….<br>…………………………………………….<br>Ansvarig läkare (namnteckning och namnförtydligande) | ……………………………………………<br>Ort och datum |

## *Appendix 8 –Case report form*

In a separate file

## *Appendix 9 –Prescription and preparation of the study product*

**Dose of fortification in order to achieve protein intake 4.0-4.5 g/kg/d:**
**Nutriprem**

| Breast milk | Feed | Nutriprem |
|---|---|---|
| protein content | volume | Dose |
| g/100 mL | ml/kg/d | |
| 0.9-1.0 | 200 | Full |
| 1.1-1.3 | 180 | Full |
| 1.4-1.7 | 160 | Full |
| 1.8-2.0 | 160 | 75% |
| 2.1-2.2 | 160 | Half |

**PreNAN HMF**

| Breast milk | Feed | PreNAN HMF |
|---|---|---|
| protein content | volume | Dose |
| g/100 mL | ml/kg/d | |
| 0.9-1.0 | 180 | Full |
| 1.1-1.4 | 160 | Full |
| 1.5-1.7 | 160 | 75% |
| 1.8-2.1 | 160 | Half |

**$H^2$MF**

| Breast milk | Feed | H2MF |
|---|---|---|
| protein content | volume | Dose |
| g/100 mL | ml/kg/d | |
| 0.9-1.0 | 170 | Full |
| 1.1-1.6 | 160 | Full |
| 1.7-2.0 | 160 | 75% |
| 2.1-2.3 | 160 | Half |

Daily prescriptions should be done in order to ensure that intakes of all nutrients are within recommended ranges. The table above only give a overview how to prescribe the different fortifiers. The Nutrium software (Nutrium AB, Umeå, Sweden) which is provided to all study sites has included the study products in its algorithms. Support will be given to physicians and dietitians if needed for the nutritional calculations. It is not mandatory to use Nutrium for the prescription of the fortifier. Thus, some study sites will use locally developed tables instead of Nutrium to achieve the wanted protein level. *For protein content in milk, true protein concentrations measured by the breast milk analyser are used.*

Total daily volume of enteral feeding should not exceed 170 ml/kg/d when the breast milk has been fortified with a full dose of $H^2$MF, since the intakes of Calcium and Phosphorous otherwise will exceed recommendations.

Observe that additional vitamins (Multivitamin Unimedic) always needs to be prescribed to infants who receive $H^2$MF®. However, in most cased there is no need of extra Calcium and Phosphorous if the milk is fortified with $H^2$MF®, please see investigator file for instructions. $H^2$MF® contain little iron. Thus, extra iron supplement is often needed in this study group.

**How to prepare the different doses:**

**Four levels of fortification**
This is the standard protocol for most sites in the study*/

Full dose fortification
$H^2MF$ : 30 ml + 70 ml breast milk (42.9 ml added to 100 ml breast milk)
Prenan HMF ("FM85"): 4 g per 100 ml breast milk
Nutriprem: 2 sachets per 100 ml breast milk

75% fortification
$H^2MF$: 22.5 ml + 77.5 ml breast milk (29 ml added to 100 ml breast milk)
Prenan HMF: 3 g per 100 ml breast milk
Nutriprem 1½ sachet per 100 ml breast milk

Half dose fortification
$H^2MF$: 15 ml + 85 ml breast milk (17.6 ml added to 100 ml breast milk)
Prenan HMF: 2 g per 100 ml breast milk
Nutriprem: 1 sachet per 100 ml breast milk

Quarter dose fortification (rarely used)
$H^2MF$: 7.5 ml + 92.5 ml breast milk (8.1 ml added to 100 ml breast milk)
Prenan HMF: 1 g per 100 ml breast milk
Nutriprem: ½ sachet per 100 ml breast milk

*/ The NICU in Uppsala fortifies with H2MF according to this protocol, but they have chosen to continue with their previous guidelines of standard fortification with 15 levels of Prenan HMF, from full dose to 7% fortification: 4.2g, 3.9g, 3.6g, 3.3g, 3.1g, 2.8g, 2.5g, 2.2g, 1.9g, 1.7g, 1.4g, 1.1g, 0.8g, 0.6g and 0.3g.

**Eight levels of fortification**
The protocol used in the Stockholm region

Full dose fortification
H2MF : 30 ml + 70 ml breast milk (42.9 ml added to 100 ml breast milk)
Prenan HMF ("FM85"): 4 g per 100 ml breast milk

87,5% fortification
H2MF: 26.3 ml + 73.7 ml (35.6 ml added to 100 ml breast milk)
Prenan HMF: 3,5 g per 100 ml breast milk

75% fortification
H2MF: 22.5 ml + 77.5 ml breast milk (29 ml added to 100 ml breast milk)
Prenan HMF: 3 g per 100 ml bröstmjölk

62,5% fortification
H2MF: 18.8 ml + 81.2 ml breast milk (23.1 ml added to 100 ml breast milk)
Prenan HMF: 2,5 g per 100 ml breast milk

Half dose fortification
H2MF: 15 ml + 85 ml breast milk (17.6 ml added to 100 ml breast milk)
Prenan HMF: 2 g per 100 ml breast milk

37,5% fortification
H2MF: 11,3 ml + 88,7 ml breast milk (12,7 ml added to 100 ml breast milk)
Prenan HMF: 1,5 g per 100 ml breast milk

25% fortification (unusual)
H2MF: 7.5 ml + 92.5 ml breast milk (8.1 ml added to 100 ml breast milk)
Prenan HMF: 1 g per 100 ml breast milk

12,5% fortification (unusual)
H2MF: 3,8 ml + 96,2 ml breast milk 3,9 ml added to 100 ml b breast milk)
Prenan HMF: 0,5 g per 100 ml breast milk

**How to start**
The dose should be based on individual analysis of the breast milk (mother´s own milk or donor milk) when such analysis has been done. If the supplementation of the breast milk fortification starts before the first analysis has been done, a half dose fortification is recommended during the first 1-3 days in both study groups.

To assist in calculating the individual nutritional needs the computer-aided nutrition calculation program Nutrium will be used. In Nutrium the $H^2$MF fortification should be added as indicated in the above. I.e. for full dose fortification add 42,9 ml $H^2$MF to 100 ml breast milk, for 75% fortification add 29 ml $H^2$MF to 100 ml breastmilk etc.

**Fat supplement**
Fat supplements can be considered if energy intake is low. The supplement should be based on the nutritional calculations with Nutrium. The $H^2$MF group should only get human milk-based fat supplement, ProlactCR®. This product will be supplied by Prolacta Bioscience during the whole intervention period for free. After the intervention has ended*, i.e.* after gestational week 34+0, Prolact CR shall be exchanged to standard fat supplement. A specific transition period is not needed for this. The standard group will get the fat supplement that is standard at the unit.

## *Appendix 10 – Transition to discharge feeding regimen*

Needed for infants in the $H^2$MF arm when weaning starts at gestational week 34+0. Also, needed for infants in both arms if weaning starts before gestational week 34+0 at a centre without the logistics for maintaining intervention.

1. **Transition protocol in infants that will continue with 100% breast milk after the transition from $H^2$ MF. The amount of the fortification (none, half or full supplementation) will be calculated as described in section 9.1.5 and Appendix 9.**

| **$H^2$ MF transition protocol: in infants that will continue with 100% breast milk** | | | | | |
|---|---|---|---|---|---|
| **Feeding Schedule**<br><br>**8 times/day Feedings:** | **Last day of all Human milk** | **Transition Day 1**<br><br>BMd/BMe+ **Standard fortifier/** HUM fortifier | **Transition Day 2**<br><br>BMd/BMe+ **Standard fortifier/** HUM fortifier | **Transition Day 3**<br><br>BMd/BMe+ **Standard fortifier/** HUM fortifier | **Transition Day 4**<br><br>BMd/BMe+ **100% Standard fortifier** |
| **1** | BMd/BMe+ HUM fortifier | BMd/BMe+ **Standard fortifier** | BMd/BMe+ **Standard fortifier** | BMd/BMe+ **Standard fortifier** | BMd/BMe+ **Standard fortifier** |
| **2** | BMd/BMe+ HUM fortifier | BMd/BMe+ HUM fortifier | DBM/EBM+ HUM Fortifier | BMd/BMe+ **Standard fortifier** | BMd/BMe+ **Standard fortifier** |
| **3** | BMd/BMe+ HUM fortifier | BMd/BMe+ HUM fortifier | BMd/BMe+ **Standard fortifier** | BMd/BMe+ **Standard fortifier** | BMd/BMe+ **Standard fortifier** |
| **4** | BMd/BMe+ HUM fortifier | BMd/BMe+ HUM fortifier | DBM/EBM+ HUM Fortifier | DBM/EBM+ HUM Fortifier | BMd/BMe+ **Standard fortifier** |
| **5** | BMd/BMe+ HUM fortifier | BMd/BMe+ **Standard fortifier** | BMd/BMe+ **Standard fortifier** | BMd/BMe+ **Standard fortifier** | BMd/BMe+ **Standard fortifier** |
| **6** | BMd/BMe+ HUM fortifier | BMd/BMe+ HUM fortifier | DBM/EBM+ HUM Fortifier | BMd/BMe+ **Standard fortifier** | BMd/BMe+ **Standard fortifier** |
| **7** | BMd/BMe+ HUM fortifier | BMd/BMe+ HUM fortifier | BMd/BMe+ **Standard fortifier** | BMd/BMe+ **Standard fortifier** | BMd/BMe+ **Standard fortifier** |
| **8** | BMd/BMe+ HUM fortifier | BMd/BMe+ HUM fortifier | DBM/EBM+ HUM Fortifier | DBM/EBM+ HUM Fortifier | BMd/BMe+ **Standard fortifier** |

Footnote: BMd=donor breast milk; BMe=mother´s own expressed breast milk: HUM fortifier= $H^2$ MF; Standard fortifier= bovine protein-based fortifier (Nutriprem, FM85 etc.)

2. **Transition protocol is not required in infants that will continue with 100% breast milk in bovine protein-based fortifier group.**

In practice, the staff in breast milk kitchen will not change anything but only provide the fortified milk

3. **Transition protocol in infants that will continue with formula (exclusively or partially) after the transition from $H^2$ MF.**

| $H^2$ MF <u>transition Protocol:</u> in infants that will continue with formula (partial or exclusive) | | | | | |
|---|---|---|---|---|---|
| <u>Feeding Schedule</u><br><br><u>8 times/day Feedings:</u> | <u>Last Day of all Human milk</u> | <u>Transition Day 1</u><br><br>BMd/BMe/ Formula+ HUM fortifier | <u>Transition Day 2</u><br><br>BMd/BMe/ Formula+ HUM fortifier | <u>Transition Day 3</u><br><br>BMd/BMe/ Formula+ HUM fortifier | <u>Transition Day 4</u><br><br>Formula **100% Standard fortifier** |
| <u>1</u> | BMd/BMe+ HUM fortifier | **Formula** | **Formula** | **Formula** | ***Formula/BMe** |
| <u>2</u> | BMd/BMe+ HUM fortifier | BMd/BMe+ HUM fortifier | BMd/BMe+ HUM fortifier | **Formula** | ***Formula/BMe** |
| <u>3</u> | BMd/BMe+ HUM fortifier | BMd/BMe+ HUM fortifier | **Formula** | **Formula** | ***Formula/BMe** |
| <u>4</u> | BMd/BMe+ HUM fortifier | BMd/BMe+ HUM fortifier | BMd/BMe+ HUM fortifier | BMd/BMe+ HUM fortifier | ***Formula/BMe** |
| <u>5</u> | BMd/BMe+ HUM fortifier | **Formula** | **Formula** | **<u>Formula</u>** | ***Formula/BMe** |
| <u>6</u> | BMd/BMe+ HUM fortifier | BMd/BMe+ HUM fortifier | BMd/BMe+ HUM fortifier | **<u>Formula</u>** | ***Formula/BMe** |
| <u>7</u> | BMd/BMe+ HUM fortifier | BMd/BMe+ HUM fortifier | **Formula** | **Formula** | ***Formula/BMe** |
| <u>8</u> | BMd/BMe+ HUM fortifier | BMd/BMe+ HUM fortifier | BMd/BMe+ HUM fortifier | BMd/BMe+ HUM fortifier | ***Formula/BMe** |

Footnote: BMd=donor breast milk; BMe=mother´s own expressed breast milk: HUM fortifier= $H^2$ MF; Standard fortifier= bovine protein-based fortifier (Nutriprem, FM85 etc.). Formula=the standard formula at the actual site.

*If the infant is partially fed with breast milk and the breast milk still are going to be fortified with protein fortifier, one or feeding with formula will be replaced with breast milk+ standard fortification.

4. **Transition protocol is not required in infants that will continue formula in bovine protein-based fortifier group.**

   The staff in the milk kitchen will follow the clinical guidelines at the neonatal unit.

## *Appendix 11 –Classification of level of care*

| | |
|---|---|
| **0** | **Infants who are not discharged but spend the whole day at home (cared for by home care team or only phone contact with neonatal unit) are cared for by home care team or who are on ”leave”** |
| **1** | **Infants who do not require continuous monitoring by health care staff.**<br><br>Example:<br>Infant stays with parents in family room without continuous monitoring. |
| **2** | **Infants who require continuous monitoring by staff and/or monitoring equipment**<br><br>Example:<br>Treatment with low flow oxygen cannula<br>Peripheral venous line |
| **3** | **Low level intensive care: Infants who require continuous monitoring and CPAP treatment or central venous access.**<br><br>Example:<br>Treatment with CPAP<br>Central venous lines for nutrition<br>Post-operative monitoring without need for respirator |
| **4** | **Medium level intensive care: Infants who require continuous monitoring by intensive care staff**<br><br>Example:<br>Stable on respirator treatment |
| **5** | **High level intensive care: Infants who require continuous monitoring by more than one intensive care staff**<br><br>Example:<br>Unstable infant on respirator treatment (respirator and need of NO and/or inotropic support) |

## *Appendix 12 –Blood sample: instructions*

## Provrör och märkning:

Provrör finns i särskild låda ipå neonatalavdelningen (*valfritt att lägga till mer exakt beskrivning på studieorten*). Det har särskild klisteretiketter för varje enskilt prov uppdelat på blod- avförings- urin- respektive bröstmjölksprov samt barnets ålder vid provtagningen.

## Blodprov:

**Dag 7, 14, och dag 28 provet**

Dessa prover skall tas vid 7±2, 14±2, respektive 28±2 dagars ålder. Använd rör avsedda för dag 7, 14, och 28.

**Blodprov i vecka 33**

Detta prov skall tas i gestationsvecka 33+0-33+6
Ta **två** EDTA-rör i denna vecka så att vi får mer provmaterial. Ta dock fortfarande bara ett CyTOF-rör.

**Hantering av blodprover:**

1. Välj rör och klisteretikett med studienamn och ”Blod/CyTOF Dag 7”, ” Blod/CyTOF Dag 14”, ” Blod/CyTOF Dag 28” respektive ”B Blod/CyTOF gv 33”, respektive ”Blod/EDTA Dag 7”, ” Blod/EDTA Dag 14”, ” Blod/EDTA Dag 28” respektive ”B Blod/EDTA gv 33”. Särskilda etiketter finns även för frysrören efter centrifugeringen och separationen plasma och celler från EDTA-röret, vg se nedan. Man behöver endast fylla i barnets studie-ID nummer och provtagningsdatum.
2. Prov tas i två olika rör:
    a. CyTOF/masscytometri: Ca 100 mikroliter (0.1 ml) blod i rör med fixeringslösning för masscytometri.
    b. EDTA-rör: Ca 500 mikroliter (0.5 ml) blod i EDTA-rör (kapillärrör)

3. Provet för masscytometri blandas genom att vändas 5 – 6 ggr, låt stå i rumstemperatur 10 min och fryses sedan i -70°C, men kan korttidsförvaras i -20°C.
4. Provet i EDTA-röret blandas genom att vändas 10 ggr. Röret får stå i rumstemperatur högst 2 timmar och sedan måste det ha tagits om hand och frysts in enligt nedan.
5. EDTA-röret centrifugeras 15 minuter, 1500g
6. Från EDTA röret sugs plasma ut med pipett och sätts i ett 0.5 ml mikroör och detta centrifugeras en gång till i 15 minuter i 1500g. Plasman pipetteras över till nya mikrorör, gärna 2-3 rör om det går (minst 100 mikroliter/rör). Märk frysröret med klisteretikett ”Plasma/EDTA dag X” och skriv studie-ID nummer och datum.
7. EDTA-röret med kvarvarande celler skall också sparas i frys. Pipettera över cellerna till 0,5 ml mikrorör. Märk frysröret med klisteretikett ”Celler/EDTA dag X” och skriv studie-ID nummer och datum.
8. Proverna kan korttidsförvaras i -20 °C innan långtidsförvaring i -70 °
9. Skriv in i CRF att prov har tagits och om något avvek från ordinarie rutiner.

*Detta är grundinstruktionen men enskilda studieorter kan justera instruktionen beroende på hur man har kommit överens med sitt lokala lab. Exempelvis kommer lab och hämtar provet före centrifugering på vissa studieorter.*

## *Appendix 13 –Stool sample: instructions*

Provrör finns i särskild låda på neonatalavdelningen (*valfritt att lägga till mer exakt beskrivning på studieorten)*. Det finns särskilda klisteretiketter för varje enskilt prov uppdelat på blod- avförings- urin- respektive bröstmjölksprov samt barnets ålder vid provtagningen.

## Avföringsprov:

**Avföringsprovet tas med spatel som sitter i locket i provröret. Det går bra att ta provet från blöjan. Ta så mycket som möjligt, d.v.s. hela rör. Det går bra att ta flera prov under dygnet, särskilt om det blir lite prov i de första rören. Sträva efter att ta 2-3 provrör.**

1. Avföringsprover skall tas vid 7±2, 14±2, 21±2 respektive 28±2 dagars ålder samt i gestationsvecka 33 (33+0-33+6).
2. Ta 2 rör per tillfälle. Sätt provet i frysfack, -20°C, i ”sköljen” i väntan på att de hämtas av labpersonal.
3. Välj rör och klisteretikett med studienamn ”Feces **dag 7**”, ”Feces **dag 14**”, ”Feces **dag 21**”,”Feces **dag 28**” respektive ”Feces **gv 33**”. Ni behöver endast fylla i barnets studie-ID nummer och provtagningsdatum.
4. Om föräldrarna tagit v33 provet hemma tar de med sig detta fryst (”iskubpåsar” i plastpåse). Provet sätts i frysfack i ”sköljen” i väntan på att de hämtas av labpersonal.
5. Ange i CRF att provet har tagits och hur många rör totalt som sparats.

*Detta är grundinstruktionen men enskilda studieorter kan justera instruktionen beroende på hur man har kommit överens med sitt lokala lab.*

## *Appendix 14 –Urine sample: instructions*

## Provrör och märkning:

Provrör finns i särskild låda på neonatalavdelningen (*valfritt att lägga till mer exakt beskrivning på studieorten*). Det finns särskilda klisteretiketter för varje enskilt prov uppdelat på blod- avförings- urin- respektive bröstmjölksprov samt barnets ålder vid provtagningen.

## Urinprov:

Ett särskilt sterilt rör för urinprov skall användas (salivette). Detta rör har en övre kammare med ett litet hål i botten. I denna övre kammare finns en torr bomullsrulle (eller steril kompress som klippts till) som man sätter i barnets blöja. Den suger upp urinen. När den är blöt sätter man tillbaka den i röret och centrifugerar ut urinen som kommer ner i botten på röret genom hålet. Helst skall det första provet efter kl 8.00 timmar som inte kontaminerats av avföring användas. Det skall in i frys så snabbt som möjligt. Det är ok att ta flera rör (vid olika tillfällen), särskilt om man fick liten urinmängd i första provet.

Som alternativ till bomullsrulle kan steril kompress användas. Den rullas ihop så att den passar i övre facket i röret.

**Hantering av urinprover:**

1. Urinprover skall tas vid 7±2, 14±2, 21±2 respektive 28±2 dagars ålder samt i gestationsvecka 33 (33+0-33+6).
2. Välj rör och klisteretikett med studienamn ” Urin dag 7”, ”Urin Dag 14”, Urin Dag 21”, ”Urin Dag 28” respektive ”Urin gv 33”. Ni behöver endast fylla i barnets studie-ID nummer och provtagningsdatum.
3. Ta ur bomullsrulle och sätt i blöja efter 8.00. Om provet kontamineras med avföring får man göra om det med ny bomullsrulle/steril kompress. Som alternativ till bomullsrulle kan steril kompress användas. Den rullas ihop så att den passar i övre facket i röret.
4. Ta hand om bomullsrullen så fort som möjligt när det blivit blöt så att den inte ligger länge i blöjan.
5. Sätt bomullsrulle i det specifika röret. Centrifugera i minst 1500g i 2-5 min. Det skall centrifugeras inom 1 timme för att urinprovet skall komma i frys så fort som möjligt. Urinen hälls/pipetteras över i frysrör.
6. Provröret sätts i kyl omgående och efter centrifugeringen sätts provet i frys, -20°C eller ´-70°C
7. Skriv in i CRF att provets tagits och hur många rör som sparats.

*Detta är grundinstruktionen men enskilda studieorter kan justera instruktionen beroende på hur man har kommit överens med sitt lokala lab.*

## *Appendix 15 –Breast milk sample: instructions*

### Provrör och märkning:

Provrör finns i särskild låda på neonatalavdelningen (*valfritt att lägga till mer exakt beskrivning på studieorten*). Det finns särskilda klisteretiketter för varje enskilt prov uppdelat på blod- avförings- urin- respektive bröstmjölksprov samt barnets ålder vid provtagningen.

### Bröstmjölksprov:

Bröstmjölksprovet tas av den bröstmjölk som barnet skall få den aktuella dagen. Om det handlar om dag 14 provet så tas alltså provet från den bröstmjölk som gjorts i ordning för dag 14. Provet skall tas **efter** berikning gjorts (eller om berikning ej påbörjad ännu tas provet av den bröstmjölk som barnet ska få). Ta så mycket som möjligt, 10 ml om möjligt, d.v.s. hela rör (men högst till 1 cm från rörkanten eftersom vätskan expanderar vid frysning).

OBS, bröstmjölksprov vid 7, 14, 21 och 28 dagar skall bara tas om det finns ett överskott av bröstmjölk från mamma, d.v.s. om mammans sparade mjölk räcker till barnets aktuella dygnsbehov. Vid gestationsvecka 33-0-36+6 tas provet även om mammans mjölk inte räcker till full tillmatning.

Om barnet vid den aktuella tidpunkten får helt eller delvis donatorsmjölk så tas provet från denna mjölk istället (efter berikning). Markera i CRF om det var donators eller mammas egna mjölk som provet togs från.

Eftersom vi skall ta berikad bröstmjölk måste mjölkköket bereda 10 ml mer bröstmjölk än barnet behöver den aktuella dagen. Meddela mjölkköket detta senast dagen före provtagning.

1. Bröstmjölksprover skall tas dag vid 7±2, 14±2, 21±2 respektive 28±2 dagars ålder samt i gestationsvecka 33+0-33+6
2. Bröstmjölksprovet ska tas **efter** ev. tillsatt berikning! Skaka flaskan med mjölken direkt innan provet tas.
3. Ta 1 rör per tillfälle. Sätt provet i frysfack, -20°C, i ”sköljen” i väntan på att de hämtas av labpersonal.
4. Välj rör och klisteretikett med studienamn BM **dag 7**”, ”BM **dag 14**”, ”BM **dag 21**”, ”BM **dag 28**” respektive ”BM **gv 33**. Ni behöver endast fylla i barnets studie-ID nummer och provtagningsdatum.
5. Skriv in i CRF att provet har tagits.

*Detta är grundinstruktionen men enskilda studieorter kan justera instruktionen beroende på hur man har kommit överens med sitt lokala lab.*

## *Appendix 16 –Nutritional content of $H^2$ MF and Prolact CR*

| Nutrient | Units | Prolact $H^2$MF | Prolact CR (Cream) |
|---|---|---|---|
| Energy | kcal/100mL | 143.82 | 262.22 |
| Fats | g/100 mL | 9.38 | 25.59 |
| Proteins | g/100 mL | 5.92 | 0.82 |
| Carbohydrates | g/100 mL | 8.91 | 6.92 |
| Saturated Fats | g/100 mL | 3.93 | 10.15 |
| Sugar | g/100 mL | 6.12 | 4.53 |
| Vitamin A Retinol | µg/100 mL | 46.21 | 114.65 |
| Vitamin D3 | µg/100mL | 0.21 | 0.09 |
| Vitamin K | µg/100 mL | <5.10 | <4.85 |
| Vitamin C | mg/100 mL | <1.02 | <0.97 |
| Thiamine | µg/100 mL | 5.92 | <4.85 |
| Riboflavin | µg/100 mL | 19.18 | 11.14 |
| Vitamin B6 | µg/100 mL | <5.10 | <4.85 |
| Niacin | mg/100 mL | 0.08 | 0.06 |
| Folate (Folic Acid) | µg/100 mL | 13.06 | <2.43 |
| Vitamin B12 (Pyridoxine) | µg/100 mL | <0.14 | <0.14 |
| Pantothenic acid | mg/100 mL | 0.17 | 0.15 |
| Biotin | µg/100 mL | <2.55 | <3.17 |
| Vitamin E (α-Tocopherol) | mg/100 mL | 0.37 | 1.54 |
| Salts[1] | mg/100 mL | 326.10 | 17.27 |
| Sodium[1] | mg/100 mL | 130.69 | 6.77 |
| Chloride[1] | mg/100 mL | 101.79 | N/A |
| Potassium[1] | mg/100 mL | 187.17 | 64.83 |
| Calcium[1] | mg/100 mL | 339.86 | 19.82 |
| Phosphorus[1] | mg/100 mL | 189.20 | 2.83 |
| Magnesium[1] | mg/100 mL | 21.48 | 0.02 |
| Iron[1] | mg/100 mL | 0.13 | 0.33 |
| Zinc[1] | mg/100 mL | 2.28 | 0.03 |
| Copper[1] | µg/100 mL | 237.5 | <0.03 |
| Iodine | µg/100 mL | 23.36 | 19.89 |
| Selenium | µg/100 mL | 9.65 | 2.07 |
| Manganese | µg/100 mL | 5.11 | <4.03 |
| Chromium | µg/100 mL | 1.57 | <0.41 |
| Molybdenum | µg/100 mL | 2.55 | <1.94 |
| Fluoride | mg/100 mL | <0.02 | <0.02 |

## *Appendix 17 –Product description and safety information*

# Prolact+6 $H^2MF$® 30mL

**Human Milk Fortifier** (Human, Pasteurized)

## PRODUCT DESCRIPTION

**Prolact+6 $H^2MF$** Concentrated Human Milk Fortifier (Human, Pasteurized)

- Each mL of **Prolact+6 $H^2MF$** contains 1.41 kcal and 0.06 grams of protein.
- Store at: -20°C or colder until ready to thaw for preparation and use.
- Available frozen in 125mL bottles containing 30mL of product.

## DIRECTIONS FOR THAWING

***Under no circumstances should Prolact+6 H2MF be defrosted or warmed in a microwave.***

Remove bottle from the freezer and **label with date and time.** Thaw product using any of the following methods:

1) **Refrigeration:** (2°C to 8°C) Place unopened bottle in refrigerator. Once thawed, must be administered within 48 hours. Do not refreeze, keep refrigerated.
2) **Rapid Thawing:** Place bottle under lukewarm running water, or place in a water bath. Do not submerge top of bottle. Warm only until product is thawed. Continued warming, or exposure to high temperatures, could result in undesirable changes to the product. Wipe outside of bottle with appropriate disinfectant to reduce the risk of contamination. Once thawed, keep refrigerated, do not refreeze. Product must be administered within 48 hours of thawing.

## PREPARATION INSTRUCTIONS

***Always maintain aseptic technique when preparing and handling human milk. **DO NOT ADD WATER*****

Each bottle of **Prolact+6 $H^2MF$** contains 30mL of fortifier and must be mixed with human milk according to Appendix 9. Once **Prolact+6 $H^2MF$** has been properly thawed (see above), remove cap from bottle. Add human milk (expressed breast milk or banked milk) into the **Prolact+6 $H^2MF$** bottle. Swirl the bottle gently to complete the mixing process.

Once completed, the product is ready for use. Refrigerate (2°C to 8°C) until needed. Product must be administered according to the Prolacta thawing guidelines (see above), or according to hospital policy regarding human milk - whichever timeframe is shorter.

## SAFETY INFORMATION

- Prolact+6 $H^2MF$ is 100% human milk. Abrupt transitioning from this product to non-human derived nutrition in the infant's diet could result in feeding intolerance or GI complications.*
- All donors are screened for HIV 1 & 2, HTLV I & II, HBV, HCV and syphilis.
- All donor milk undergoes drug screening for amphetamine, benzodiazepine, cocaine, marijuana (THC), methamphetamine, opiates, nicotine, oxycodone, oxymorphone and their principle metabolites.
- Donor identity matching is performed on donated breast milk using DNA fingerprinting.
- Pasteurization has repeatedly demonstrated a $10^5$ reduction of pathogenic viruses such as HIV, HBV and HCV. It has been shown to kill pathogenic bacteria such as *E. coli, S. aureus* and *Klebsiella* species at levels of $10^{15}$ or higher, the precise amount varying by strain and technique.
- HIV, also a marker virus for CMV, has been repeatedly demonstrated to inactivate during pasteurization.
- Pooled human breast milk is tested by PCR for the presence of HIV-1, HBV and HCV.

## INGREDIENTS

Human milk, and contains less than 2% of one or more of the following: calcium glycerophosphate, calcium gluconate, sodium citrate, calcium chloride, calcium carbonate, potassium citrate, magnesium phosphate, zinc sulfate and cupric sulfate.

## FOR MORE INFORMATION

Visit us at www.prolacta.com or call 1(888) PROLACT

#1 for Customer Service

Manufactured by: Prolacta Bioscience, Inc. City of Industry, CA 91746

# Prolact CR™

**10mL**

## Human Milk Caloric Fortifier (Human, Pasteurized)

### PRODUCT DESCRIPTION

- **Prolact CR** is pasteurized human milk cream derived from human milk. It is composed of 25% fat and provides 2.5 Cal/mL. It contains no added minerals.
- **Store at:** -20° C or colder until ready to thaw for preparation and use.
- Available frozen in 30mL bottles containing 10mL of product (4 bottles per package).

### INTENDED USE

- **Prolact CR** is intended for use with mom's own breast milk or donor human milk to help achieve a 20 Cal/fl. oz. feeding solution.

### DIRECTIONS FOR THAWING

***Under no circumstances should the product be defrosted or warmed in a microwave.***

Remove bottle from the freezer and **label with date and time**. Thaw product using any of the following methods:

1) **Refrigeration:** (2°C to 8°C) Place unopened bottle in refrigerator. Once thawed, must be administered within 24 hours. Do not refreeze, keep refrigerated.
2) **Rapid Thawing:** Place bottle under lukewarm running water, or place in a water bath. Do not submerge top of bottle. Warm only until product is thawed. Continued warming, or exposure to high temperatures, could result in damage to the product. Wipe outside of bottle with appropriate disinfectant to reduce the risk of contamination. Once thawed, must be administered within 24 hours. Do not refreeze, keep refrigerated.

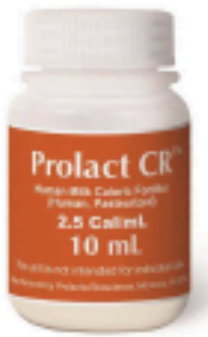


### PREPARATION INSTRUCTIONS

**Always maintain aseptic technique when preparing and handling human milk products. **DO NOT ADD WATER****

1) Thaw mom's own or donor milk according to hospital policy.
2) Measure caloric content of mom's own or donor milk.
   a. If using a commercial human milk analyzer, follow the manufacturer's instructions.
   b. If using a creamatocrit, ensure the milk is room temperature and follow the manufacturer's instructions.
3) Thaw Prolact CR according to "Directions for Thawing". Swirl gently prior to each aliquot.
4) Based on the measured caloric content of mom's own or donor milk, follow the instructions in Table 1 to formulate 100 mL of human milk plus Prolact CR.

**Table 1: Prolact CR Mixing Ratios**

| Cal/Ounce (equivalent to) | Cal/100mL | Mom's own or donor milk volume | + Add to milk |
|---|---|---|---|
| 19 - 20 | 64 - 67.9 | 98 mL milk | 2 mL Prolact CR |
| 18 - 18.9 | 61 - 63.9 | 96 mL milk | 4 mL Prolact CR |
| 17 - 17.9 | 57 - 60.9 | 94 mL milk | 6 mL Prolact CR |
| 16 - 16.9 | 54 - 56.9 | 93 mL milk | 7 mL Prolact CR |
| 15 - 15.9 | 51 - 53.9 | 91 mL milk | 9 mL Prolact CR |
| 14 - 14.9 | 47 - 50.9 | 90 mL milk | 10 mL Prolact CR |

5) Swirl gently to mix.
6) Once completed, the product is ready for use, OR
7) Store bottle in refrigerator (2°C to 8°C). Use within 24 hours after thawing Prolact CR.

### INGREDIENTS

Human milk cream and human milk ultrafiltration permeate.

### FOR MORE INFORMATION

Visit us at www.prolacta.com or call 1(888) PROLACT or 1(888) 776-5228

Manufactured by:
Prolacta Bioscience, Inc.
Monrovia, CA 91016

Prolacta Bioscience
Advancing the Science of Human Milk


© [illegible] Prolacta Bioscience
